# Characterization of Five Lytic Bacteriophages as New Members of the Genus *Mosigvirus*, Infecting Multidrug-Resistant Shiga Toxin-Producing *Escherichia coli* and Their Antibiofilm Activity

**DOI:** 10.3390/v17111501

**Published:** 2025-11-13

**Authors:** Jong Beom Na, Seungki Lee, Eun Jeong Park, Soojin Lim, Keeman Lee, Ye Bin Kim, Tae Seon Cha, Seon Young Park, Ji Hyung Kim

**Affiliations:** 1 Department of Food Science and Biotechnology, College of Bionano Technology, Gachon University, Seongnam 13120, Republic of Korea; qwer789@gachon.ac.kr (J.B.N.); pog0227@gachon.ac.kr (E.J.P.); sjlim001103@gachon.ac.kr (S.L.); 99angello@gachon.ac.kr (K.L.); zxc5620@gachon.ac.kr (Y.B.K.); walnut99@gachon.ac.kr (T.S.C.); 2 Biological and Genetic Resources Assessment Division, National Institute of Biological Resources, Incheon 22689, Republic of Korea; metany@korea.kr; 3 Veterinary Drugs and Biologics Division, Animal and Plant Quarantine Agency, Gimcheon 39660, Republic of Korea

**Keywords:** phage depolymerase, biofilm inhibition, antimicrobial resistance, control, biocontrol agents

## Abstract

The emergence of multidrug-resistant Shiga toxin-producing *Escherichia coli* (STEC) poses a major challenge to public health and necessitates the development of alternative antimicrobial strategies. This study aimed to isolate and characterize five lytic bacteriophages belonging to the genus *Mosigvirus* and evaluate their potential as biocontrol against MDR STEC strains and their biofilms. The five bacteriophages, designated vB_EcoM-pJBB (ΦB), vB_EcoM-pJBC (ΦC), vB_EcoM-pJBJ (ΦJ), vB_EcoM-pJBK (ΦK), and vB_EcoM-pJBL (ΦL), were isolated from sewage treatment plant samples using STEC ATCC 43895 as host. Biological characterization included host range determination against 19 MDR STEC strains, one-step growth analysis, environmental stability assays, bacteriolytic activity assessment, and antibiofilm efficacy testing. Whole-genome sequencing and phylogenetic analyses were performed to determine genomic features and taxonomic classification. The phages demonstrated varying infectious capacities, lysing between six and 12 strains, with ΦL exhibiting the broadest spectrum of activity. All phages showed MOI-independent antibiofilm activity, preventing biofilm formation by approximately 70% and disrupting pre-formed biofilms by up to 80.3%. Genomic analysis revealed the absence of lysogeny markers, virulence factors, and antimicrobial resistance genes, while identifying putative depolymerase genes associated with tail fiber proteins. Phylogenetic analysis confirmed the taxonomic position of these phages within the *Mosigvirus* genus in the *Straboviridae* family. Our findings indicate that the newly identified *Mosigvirus* phages are promising candidates for phage-based biocontrol applications.

## 1. Introduction

Shiga toxin-producing *Escherichia coli* (STEC) is a major foodborne pathogen that poses a considerable threat to public health worldwide. STEC strains, particularly those belonging to the O157:H7 serotype, are associated with severe gastrointestinal illnesses such as hemorrhagic colitis and hemolytic-uremic syndrome [1]. STEC produces two distinct types of Shiga toxins (Stxs): stx1 and stx2 leading to the severe symptoms associated with STEC infections [2,3]. The transmission of STEC to humans occurs through diverse environmental routes, with ruminants serving as asymptomatic carriers and their feces contaminating food products [4]. Recent studies have reported the transmission of STEC into wild animals such as boars, birds, and aquatic animals [5].

Antibiotics are commonly used to treat STEC infection; however, the increasing prevalence of antimicrobial resistance (AMR) among STEC strains has become a major concern [6]. The acquisition of extended-spectrum beta-lactamase (ESBL) genes by STEC strains detected in food sources [7] is a clinically significant AMR indicator [8,9]. Moreover, certain antibiotics can induce a stress response in STEC, thereby activating lysogenic phages and increasing toxin production [10]. The *stx* genes, located in Shiga toxin-converting prophages, can be induced and excised under antibiotic or environmental stress [11,12]. Furthermore, prophages can harbor ESBL genes, thereby facilitating the transfer and spread of AMR [13]. In addition to AMR, STEC can form biofilms that enhance resistance to disinfectants and antibiotics [14], leading to cross-contamination in food-processing environments [15]. Given the increasing prevalence of STEC pathogenicity, alternative approaches are urgently required to effectively control STEC infections.

Bacteriophages (phages) are potential biocontrol agents because their lytic mechanisms are distinct from those of antibiotics, enabling them to effectively control AMR bacteria [16]. Their host specificity prevents dysbiosis, which is a significant benefit compared with the side effects of antibiotic treatment [17]. Antibiotic sensitivity is restored, or virulence is reduced in some strains that develop resistance to phages [18,19]. Lytic phage treatment of STEC encoding stx-prophages can also interfere with prophage induction and cell lysis [20,21]. Moreover, phage-derived depolymerases naturally degrade biofilm matrices, disrupt pre-formed biofilms, and control both the biofilm and the bacteria within [22,23].

T4-like phages, including RB69, RB49, and JS98, are classified within the representative formerly known as the *Myoviridae* family and share biological and genomic characteristics [24]. These phages have a strict lytic lifecycle, infect several strains of *E. coli* and other enterobacteria, and have host specificity [25]. Genomic analysis of these phages revealed that they share conserved genes for essential functions, with specificity for proteins such as endolysin and tail fiber depolymerases [26]. RB69, a well-characterized T4-like phage, exhibits approximately 81% DNA sequence similarity, with 80% of its genes identified as orthologs of phage T4 [24]. However, RB69 shows unique variations in its host-specificity determinants, particularly in tail fiber proteins that are responsible for host recognition and binding. This feature contributes to the ability of RB69 to infect a specific range of bacterial hosts without disrupting the balance of the gut microbiota, making it a promising alternative to antibiotics.

Recent advancements in genomic analysis have enabled a more definitive classification of T4-like phages [27,28]. The International Committee on Taxonomy of Viruses (ICTV) has reclassified RB69, RB49, JS98, and related phages into the *Mosigvirus* genus within the newly established *Straboviridae* family based on their genomic and biological properties [29]. This has further refined our understanding of diversity within the T4-like phage group.

In this study, we isolated and characterized five new phages belonging to the *Mosigvirus* genus that effectively controlled both planktonic and biofilm-associated STEC. Biological and genomic characterization of these phages revealed their lytic potential against STEC, whereas phylogenetic analysis demonstrated their relationship with well-studied T4-like phages.

## 2. Materials and Methods

### 2.1. Bacterial Strains and Culture Conditions

In total, 29 bacterial strains were used in this study, including 19 STEC strains (ATCC 43895, 16 STEC isolates, NCCP 13720, and NCCP 13721), eight non-STEC strains (two isolates, BL21, ATCC 13706, ATCC 11775, ATCC 31616, ATCC 31618, and ATCC 23545), and two *Escherichia* species (*E. fergusonii* ATCC 35469 and *E*. *hermanii* ATCC 33650). The 16 STEC isolates were environmental strains obtained from the Korean Culture Center of Microorganisms (KCCM), which were originally isolated from environmental sources as previously characterized [30]. All bacterial strains were incubated on tryptic soy agar (TSA; Difco Laboratories, Detroit, MI, USA) at 37 °C for 24 h and stored in tryptic soy broth (TSB; Difco Laboratories, Detroit, MI, USA) with 10% glycerol (*v*/*v*) at −80 °C until further use.

### 2.2. Antimicrobial Susceptibility Testing

The antimicrobial susceptibility profiles of the STEC strains used in this study were determined by the disc diffusion method, as previously described [31]. The bacterial strains were inoculated onto Müller-Hinton agar plates (Oxoid Ltd., Basingstoke, Hampshire, UK), and antimicrobial discs (Oxoid Ltd., UK) were placed on the agar surface. The following antimicrobials were tested: ampicillin (10 µg), piperacillin (100 µg), amoxicillin-clavulanate (10/10 µg), ampicillin-sulbactam (10/10 µg), piperacillin-tazobactam (100/10 µg), cefepime (30 µg), cefotaxime (30 µg), ceftriaxone (30 µg), ceftazidime (30 µg), cefuroxime (30 µg), cefixime (5 µg), aztreonam (30 µg), imipenem (10 µg), meropenem (10 µg), amikacin (30 µg), streptomycin (10 µg), azithromycin (15 µg), tetracycline (30 µg), doxycycline (30 µg), ciprofloxacin (5 µg), levofloxacin (5 µg), norfloxacin (10 µg), and chloramphenicol (30 µg). The diameters of the inhibition zones were measured after incubation, and the results were interpreted according to the CLSI guidelines [32]. The resistance rate for each antibiotic was determined as the percentage of resistant strains among all tested strains, calculated as (number of resistant strains/total number of tested strains) × 100.

### 2.3. Phage Isolation and Purification

Five STEC phages were isolated from water samples collected from a sewage treatment plant in Daejeon, Republic of Korea, using STEC ATCC 43895 as the host bacterial strain, as previously described [23]. Exponential-phase STEC culture (400 µL) was inoculated into pre-heated TSB, and an equal volume of the collected samples was added. After incubation at 37 °C for 24 h, the culture was centrifuged at 10,000× *g* for 10 min, and the supernatant was filtered using a 0.45 µm membrane filter (JetBiofil, Guangzhou, China) to remove bacterial cells and large particles. Phages were isolated using the double-layer agar method. 1 mL of the phage filtrate was mixed with 1 mL of the overnight STEC culture and 7 mL of TSB soft agar (0.7% agar), gently mixed, and poured onto a TSA-bottom agar plate. The plates were incubated at 37 °C for 24 h until plaques formed. For phage purification, lysates were filtered through a 0.22 µm sterilizing-grade membrane filter (JetBiofil, Guangzhou, China) to ensure complete removal of bacterial cells. Single-plaque isolation was performed at least three times to obtain pure phage stocks. The purified phage lysates were then stored at either 4 °C or −80 °C for further use.

### 2.4. Transmission Electron Microscopy

The morphology of the isolated STEC phages was determined by TEM (JEM-1011; JEOL, Tokyo, Japan) according to the manufacturer’s instructions. 10 µL of each high-titer phage lysate (ca. 10^8^ PFU/mL) was spotted onto a carbon-coated copper grid (200 mesh; Ted Pella, Inc., Redding, CA, USA) and allowed to adsorb for 5 min at 25 °C. Excess liquid was carefully removed by blotting the edge of the grid with filter paper. The phage-adsorbed grids were negatively stained with 5% (*w*/*v*) uranyl acetate solution (Sigma-Aldrich, St. Louis, MO, USA) for 2 min at 25 °C. Excess stain was removed by blotting, and the grids were allowed to air-dry completely. Negatively stained phage samples were examined using TEM at an accelerating voltage of 80 kV. Phage dimensions, including head diameter and tail length, were measured using the ImageJ software v.1.53 [33].

### 2.5. Host Range Determination

The host range of the five isolated STEC phages was determined using a spot assay as previously described [23]. Exponential-phase bacterial cultures (100 µL) were mixed with 7 mL of 0.7% soft agar and overlaid onto TSA plates. Phage lysates were serially diluted in distilled water to obtain concentrations ranging from 10^4^ to 10^9^ PFU/mL. A 10 µL aliquot of each dilution was spotted onto the bacterial lawns and allowed to absorb for 20 min at 25 °C. The plates were then incubated at 37 °C for 24 h. The lytic activity of phages was assessed by observing the formation of clear plaques on the bacterial lawn. All host range tests were performed in triplicate.

### 2.6. One-Step Growth Curve

The one-step growth curves of the five isolated phages were determined to evaluate their burst size and latent period as previously described [23]. Exponential-phase cultures of the host strain, STEC ATCC 43895, were mixed with each phage at a low MOI of 0.001 and incubated at 37 °C for 10 min to allow phage adsorption. The mixture was then centrifuged at 10,000× *g* for 5 min to remove unabsorbed phages. The pellet containing infected bacterial cells was resuspended in 10 mL of pre-heated TSB and incubated at 37 °C with shaking. 1 mL of sample was collected at 10 min intervals for 60 min. Each sample was immediately utilized for phage titration using the double-layer agar method. All one-step growth curve experiments were performed in triplicate, and the results are presented as the mean ± standard deviation.

### 2.7. pH and Thermal Stability

The stability of the five isolated STEC phages under various pH and thermal conditions was evaluated as previously described [23]. For the pH stability assay, phage lysates (ca. 10^7^ PFU/mL) were mixed with an equal volume of 0.1 M phosphate-buffered saline and subjected to different pH levels (2–10) using 1 M HCl (Samchun Chemical, Pyeongtaek, Gyeonggi-do, Republic of Korea) or 1 M NaOH (Samchun Chemical, Pyeongtaek, Gyeonggi-do, Republic of Korea). The phage–buffer mixtures were incubated at 4 °C for 3 h to minimize the effect of temperature on phage stability. For the thermal stability assay, phage lysates (ca. 10^7^ PFU/mL) were incubated at various temperatures (0, 4, 10, 30, 40, 50, and 60 °C) for 3 h. After the respective treatments, the phage titers were determined using the double-layer agar method. All experiments were performed in triplicate, and the results are presented as the mean ± standard deviation.

### 2.8. In Vitro Bactericidal Activity

The bacterial cell lysis activity of the five isolated STEC phages was evaluated using a 96-well plate assay as previously described [34]. An overnight culture of the host strain STEC ATCC 43895 was diluted in fresh TSB to reach an early log phase suspension with a concentration of approximately 10^7^ CFU/mL. A 100 µL aliquot of this bacterial suspension was transferred to each well of a 96-well microplate (Life Sciences, Pocheon, Gyeonggi-do, Republic of Korea). Subsequently, 100 µL of phage suspensions at different MOIs (0.00001, 0.0001, 0.001, 0.01, 0.1, and 1) were added to the corresponding wells containing the bacterial suspension. TSB without phages was used as a positive control for bacterial growth. The 96-well plate was then incubated at 37 °C, and the OD_600_ was measured using a SpectraMax ABS microplate reader (Molecular Devices, San Jose, CA, USA) at seven time points: 0, 30, 60, 90, 120, 150, and 180 min. All experiments were performed in triplicate, and the results are presented as the mean ± standard deviation.

### 2.9. Biofilm Inhibition Assay

The biofilm inhibition ability of the five isolated STEC phages was evaluated using a 96-well plate assay as previously described [34]. An overnight culture of the host STEC strain (ATCC 43895) was diluted in TSB to a final concentration of approximately 10^7^ CFU/mL. Aliquots (100 µL) of diluted bacterial culture were inoculated into the wells of a 96-well microplate. Phage lysates were diluted in TSB to obtain three different MOIs (0.01, 0.1, and 1). A 100 µL aliquot of each phage dilution was added to the corresponding wells containing the bacterial suspension. TSB without phages was used as a positive control for STEC biofilm formation. The microplate was then incubated at 37 °C for 24 h under static conditions to allow biofilm formation. After incubation, planktonic cells were carefully removed by pipetting, and the wells were washed twice with distilled water to remove any remaining unattached cells. The biofilms were then stained with 200 µL of 0.1% CV solution (Sigma-Aldrich, Dorset, UK) for 20 min at 25 °C. The stain solution was removed, and the wells were washed two times to remove excess stain. The stained biofilms were solubilized by adding 200 µL of 99% ethanol to each well, and the microplate was incubated at 25 °C for 10 min. OD_595_ values were measured using a microplate reader to determine the relative cell density of the biofilms. All experiments were performed in triplicate, and the results are expressed as the mean ± standard deviation.

### 2.10. Biofilm Eradication Assay

The biofilm eradication ability of the five isolated STEC phages was assessed using a 96-well plate assay, as described in Section 2.9. An overnight culture of the host STEC strain ATCC 43895 was diluted in TSB to a final concentration of approximately 10^7^ CFU/mL. Aliquots (100 µL) of the diluted bacterial culture were inoculated into the wells of a 96-well microplate and incubated at 37 °C for 24 h to allow biofilm formation. After incubation, planktonic cells were carefully removed by pipetting, and the wells were washed twice with sterile distilled water to remove any remaining unattached cells. Phage lysates were diluted in TSB to obtain three different MOIs (0.01, 0.1, and 1). A 100 µL aliquot of each phage dilution was added to the corresponding wells containing the pre-formed biofilms and incubated at 37 °C for an additional 24 h. TSB without phage was used as a positive control for biofilm growth. After the second incubation, the wells were washed twice with distilled water to remove any remaining phage particles and planktonic cells. The biofilms were subsequently stained, solubilized, and measured as described in Section 2.9.

### 2.11. Confocal Laser Scanning Microscopy

Live cells in the phage-treated biofilms were visualized using CLSM and SYBR Gold staining as previously described [35]. An overnight culture of the host STEC strain ATCC 43895 was diluted in TSB to reach an early log phase suspension at a concentration of approximately 10^7^ CFU/mL. The bacterial suspension was then mixed with phage lysates at an MOI of 0.01. Sterile glass coverslips (Marienfeld-Superior, Lauda-Königshofen, Germany) were placed in a non-surface-treated 6-well plate (SPL Life Sciences, Pocheon, Gyeonggi-do, Republic of Korea), and 2 mL of the bacteria-phage mixture was added to each well. The plate was incubated at 37 °C for 24 h to allow biofilm formation on the coverslips. The inoculum was carefully removed after incubation, and the biofilms on the coverslips were gently washed with distilled water to remove planktonic cells. Biofilms were stained with 1 μL of 100× SYBR Gold nucleic acid stain (Invitrogen, Carlsbad, CA, USA) for 30 min at 25 °C in the dark. Following staining, the coverslips were gently washed with distilled water to remove excess stain and mounted on slide glass (Marienfeld-Superior, Lauda-Königshofen, Germany). Stained biofilms were observed under a CLSM (Ti-E; Nikon Corporation, Tokyo, Japan) using a 60× immersion objective lens. Live cells within the biofilms were distinguished based on their fluorescence emission properties. Cells with intact membranes exhibited green fluorescence upon uptake of the SYBR Gold stain, which was detected at excitation and emission wavelengths of 488 nm and 539 nm, respectively.

### 2.12. Genome Sequencing and In Silico Analysis

Genomic DNA (gDNA) of the five isolated STEC phages was extracted from high-titer phage lysates (10^8^ PFU/mL) as previously described [23]. The extracted gDNA was sequenced using the Illumina HiSeq X-10 platform (Illumina, Inc., San Diego, CA, USA) at Macrogen, Inc., Seoul, Republic of Korea. Library preparation was performed using a TruSeq Nano DNA Library Prep Kit (Illumina, Inc., San Diego, CA, USA), followed by paired-end sequencing (2 × 151 bp). Raw sequencing data were subjected to quality control using Trimmomatic v.0.36 (http://www.usadellab.org/cms/index.php?page=trimmomatic, accessed on 28 November 2023). Subsequently, the filtered reads (ΦB: 14,961,642; ΦC: 14,894,484; ΦJ: 15,788,242; ΦK: 16,127,900; ΦL: 16,858,110) were assembled de novo using SPAdes Genome Assembler v.3.13.0 (http://bioinf.spbau.ru/spades, accessed on 10 December 2023) with default settings. This resulted in assemblies with the average coverage of the sequences (ΦB: 178×; ΦC: 169×; ΦJ: 12,170×; ΦK: 12,610×; ΦL: 13,135×). Next, annotation was performed using the Rapid Annotation using Subsystem Technology server v.2.0 (https://rast.nmpdr.org, accessed on 31 December 2023). The identified ORFs were functionally predicted to amino acid sequence-based homology searches using BlastP (https://www.ncbi.nlm.nih.gov/, accessed on 15 March 2024), Pfam (https://www.ebi.ac.uk/interpro/, accessed on 18 March 2024), and HHpred databases (https://toolkit.tuebingen.mpg.de/tools/hhpred, accessed on 18 March 2024). Transmembrane domains and signal peptides in the phage genomes were detected and quantified using TMHMM 2.0 (https://services.healthtech.dtu.dk/services/TMHMM-2.0/, accessed on 21 April 2024) [36] and Signal P v.6.0 (https://services.healthtech.dtu.dk/services/SignalP-6.0/, accessed on 21 April 2024) [37]. Genome termini and packaging mechanisms were predicted using PhageTerm (https://sourceforge.net/projects/phageterm, accessed on 22 April 2024) [38]. Putative tRNA and phage depolymerase-encoding genes in the genome were screened using tRNAscan-SE 2.0 (https://lowelab.ucsc.edu/tRNAscan-SE/, accessed on 24 April 2024) [39] and DePP (https://timskvortsov.github.io/WebDePP/, accessed on 3 July 2024) [40]. AMR- and potential virulence factor-associated genes were examined using the Comprehensive Antibiotic Resistance Database server (https://card.mcmaster.ca/, accessed on 1 May 2024) [41] and the Virulence Factor Database (http://www.mgc.ac.cn/VFs/, accessed on 1 May 2024) [42]. Whole-genome maps of the phages were visualized using the Proksee server (https://proksee.ca/, accessed on 15 May 2024) [43].

### 2.13. Comparative Genomic Analysis

Genome-based phylogenies of the five isolated STEC phages and related phage genomes obtained from the GenBank database were constructed using the Genome-BLAST Distance Phylogeny method version as of 2013 on the VICTOR (https://ggdc.dsmz.de/home.php, accessed on 20 March 2024) server [44]. Gene phylogenies of the phages were generated using the maximum likelihood method with 1000 bootstrap replicates in MEGA 11 (https://www.megasoftware.net/, accessed on 20 March 2024) [45]. This analysis was based on the amino acid sequences of the terminase large subunit and major capsid protein, which were aligned using Clustal X v.2.1 (http://www.clustal.org/clustal2/, accessed on 22 March 2024) [46] and refined with the BioEdit v.7.2.5 software (https://www.softpedia.com/get/Science-CAD/BioEdit.shtml, accessed on 22 March 2024). Nucleotide-based intergenomic similarities between the isolated phages and their closest relative phage genomes were calculated using VIRIDC (https://rhea.icbm.uni-oldenburg.de/viridic/, accessed on 1 August 2024) [47], and the resulting similarity matrix was visualized as a heatmap heat map. Genomes of the isolated phages were compared using tBLASTx (https://blast.ncbi.nlm.nih.gov/Blast.cgi?PROGRAM=tblastx, accessed on 10 April 2024) and visualized using EasyFig (https://mjsull.github.io/Easyfig/, accessed on 10 April 2024) [48] to identify conserved and divergent regions across the phage genomes. Comparative analyses between the phages and their closest relatives were performed using VipTree (https://www.genome.jp/viptree/, accessed on 15 August 2024) [49] based on the resulting genomic phylogenetic tree, which revealed two distinct branches of the phages.

### 2.14. Statistical Analysis

All statistical analyses were conducted using either an independent samples t-test or one-way analysis of variance using XLSTAT software v.2021.3.1 (Addinsoft, Paris, France) in Microsoft Excel (Office 365; Microsoft Corp., Redmond, WA, USA). *p* < 0.01 was considered statistically significant.

## 3. Results

### 3.1. Antimicrobial Susceptibility Testing of STEC Strains

The antimicrobial susceptibility of STEC strains to various classes of antimicrobial agents was evaluated using the standard disc diffusion method according to the Clinical Laboratory Standards Institute (CLSI) guidelines [32] (Table 1). In this study, all STEC strains were classified as MDR as they exhibited non-susceptibility to at least one agent in three or more antimicrobial classes. High levels of resistance were observed against three antimicrobial classes: penicillin (ampicillin, resistance rate 78.9%), β-lactam agents (amoxicillin-clavulanate, resistance rate 89.5%; ampicillin-sulbactam, resistance rate 94.7%), and cephalosporins (cefotaxime, resistance rate 84.2%; ceftazidime, resistance rate 78.9%). Moreover, moderate resistance levels were observed for four antimicrobial classes, including monobactam (aztreonam, resistance rate 42.1%) and aminoglycosides (amikacin, resistance rate 31.6%; streptomycin, resistance rate 57.9%). Conversely, low resistance levels were observed for carbapenem (imipenem, resistance rate 11%), macrolides (azithromycin, resistance rate 5.3%), tetracyclines (tetracycline, resistance rate 15.8%; doxycycline, resistance rate 26.3%), fluoroquinolones (levofloxacin, resistance rate 11%; norfloxacin, resistance rate 5.3%), and phenicol (chloramphenicol, resistance rate 15.8%).

### 3.2. Isolation and Morphological Characteristics of Five STEC Phages

**Figure 1:**
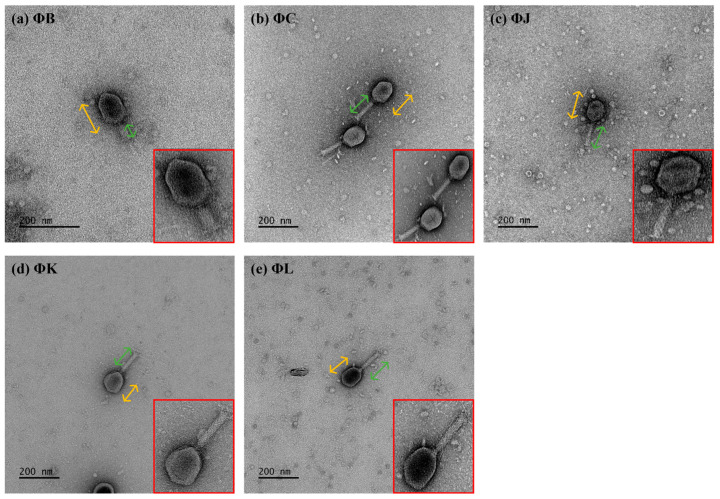
Morphological characterization of the five newly isolated Shiga toxin-producing *Escherichia coli* (STEC) phages. All phages exhibited an icosahedral head (yellow arrows) and a contractile tail (green arrows), characteristic of *Myoviridae* morphology (shown at higher magnification in red boxes). Scale bars represent 200 nm.

Five phages, designated vB_EcoM-pJBB (ΦB), vB_EcoM-pJBC (ΦC), vB_EcoM-pJBJ (ΦJ), vB_EcoM-pJBK (ΦK), and vB_EcoM-pJBL (ΦL), were isolated from water samples collected at a sewage treatment plant in Daejeon, Republic of Korea, using STEC strain ATCC 43895 as the host. Transmission electron microscopy (TEM) revealed that all five phages had similar morphological features, such as an icosahedral head and contractile tail. The head diameter of the phages was relatively consistent among four phages: 107.5 ± 2.1 nm (ΦB), 113.3 ± 3.4 nm (ΦC), 112.7 ± 1.5 nm (ΦJ), 103.9 ± 2.6 nm (ΦK), and 108.28 ± 1.0 nm (ΦL), as determined from measurements of 10 particles per phage (Figure 1). The tail lengths of the phages were 112.1 ± 2.2 nm (ΦC), 109.1 ± 3.1 nm (ΦJ), 123.7 ± 3.2 nm (ΦK), and 112.9 ± 6.1 nm (ΦL), whereas ΦB exhibited a significantly shorter tail at 57.1 ± 2.2 nm.

### 3.3. Host Ranges of Five Isolated STEC Phages

The five isolated STEC phages exhibited varying lytic spectra, with four phages (ΦB, ΦC, ΦJ, ΦK, and ΦL) demonstrating a broad host range, effectively infecting at least six STEC strains out of the 19 host bacteria tested (Table 2). ΦB exhibited lytic activity against 10 strains, whereas both ΦC and ΦJ lysed nine strains each., and ΦL showed the broadest host range, infecting 12 strains. In contrast, ΦK demonstrated the lowest host susceptibility, lysing only six out of the 19 STEC strains tested. Approximately half of the non-STEC strains were susceptible to phages, resulting in a diverse host spectrum. All the phages lysed *E. fergusonii* ATCC 35469, whereas none lysed *E*. *hermanii* ATCC 33650.

### 3.4. One-Step Growth Curves of Five Isolated STEC Phages

**Figure 2:**
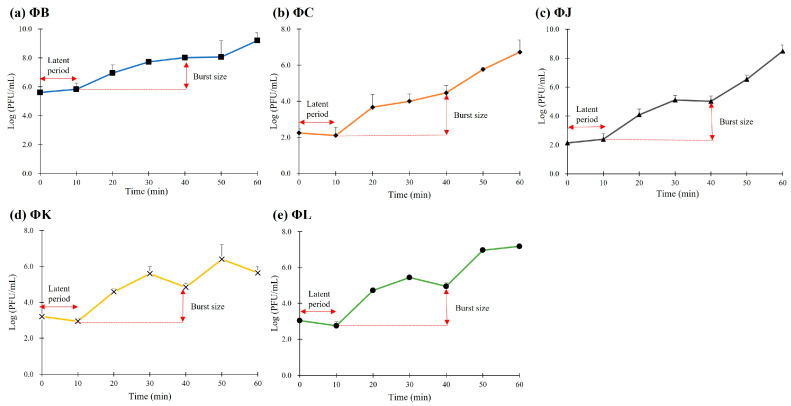
One-step growth curve analysis of the five newly isolated STEC phages. All phages exhibited a latent period of 10 min and burst sized ranging from 78 to 427 PFU/mL. Data points represent the mean values, and error bars indicate the standard deviation of three independent experiments.

One-step growth curve analyses were performed at a multiplicity of infection (MOI) of 0.001 to determine the latent period and burst size of the five isolated STEC phages (Figure 2). All phages demonstrated consistent latent times of approximately 10 min and burst periods of approximately 30 min. However, the phages showed notable differences in burst size. ΦB, ΦC, and ΦL exhibited similar numbers of reproductive virion particles, with 154.8, 230, and 157.5 plaque-forming units (PFU) per infected cell, respectively. ΦJ showed the highest burst size at 427.4 PFU per infected cell, whereas ΦK exhibited the lowest burst size at only 78.7 PFU per infected cell.

### 3.5. Thermal and pH Stabilities of the Five Isolated STEC Phages

**Figure 3:**
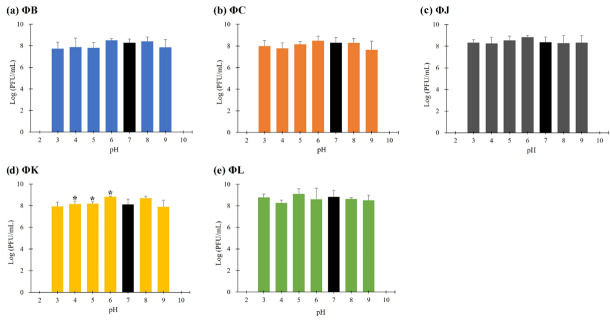
pH stability of the five newly isolated STEC phages. Data points represent the mean values, and error bars indicate the standard deviations of three independent experiments. Asterisks (*) indicate a statistically significant difference compared with the control (pH 7, black bars) (*p* < 0.05).

**Figure 4:**
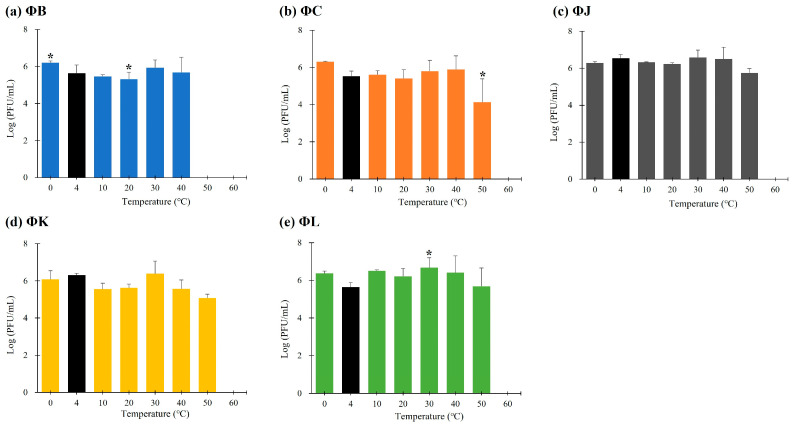
Thermal stability of the five newly isolated STEC phages. Data points represent the mean values, and error bars indicate the standard deviations of three independent experiments. Asterisks (*) indicate a statistically significant difference compared with the control (4 °C, black bars) (*p* < 0.05).

The stability of the five isolated STEC phages was investigated at pH values ranging from 2 to 10 after 3 h of incubation (Figure 3). All phages were stable within the pH range of 3–9; however, their viability was significantly reduced at pH levels < 2 and >10. The thermal stability of the five isolated STEC phages was evaluated at temperatures ranging from 0 to 60 °C following 3 h incubation (Figure 4). Each phage maintained its viability up to 40 °C; however, only three phages (ΦJ, ΦK, and ΦL) remained viable at 50 °C. ΦB completely lost its infectivity, whereas ΦC showed a reduction in phage titer of approximately 10^2^ PFU per infected cell under these thermal conditions. Exposure to temperatures above 60 °C completely inactivated all phage particles.

### 3.6. Bacteriolytic Activities of the Five Isolated STEC Phages

**Figure 5:**
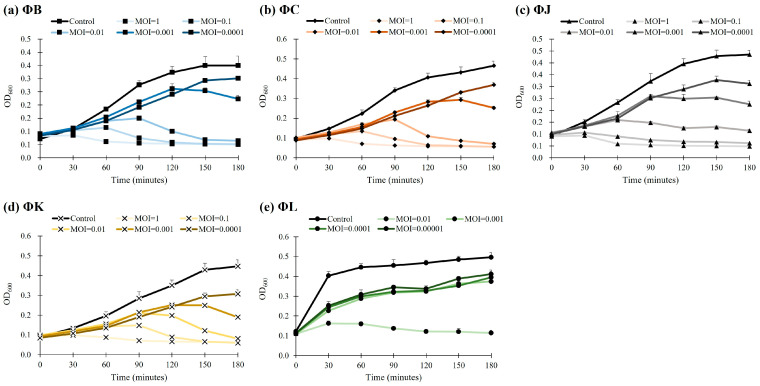
Bacteriolytic activity of the five newly isolated STEC phages. The control group represents STEC ATCC 43895 without phage treatment. Data points represent the mean values of three independent experiments, and error bars indicate the standard deviations.

The bacteriolytic activity of the five isolated STEC phages was evaluated by measuring the reduction in bacterial cell density using optical density at 600 nm (OD_600_). The analyses revealed a concentration-dependent inhibition of bacterial growth across all phages (Figure 5). At an MOI of 0.01, each phage effectively inhibited the growth of host bacteria for up to 90 min, resulting in a reduction in cell density by approximately 82.7% (ΦB), 87.2% (ΦC), 75.3% (ΦJ), 83.2% (ΦK), and 74.5% (ΦL). At a lower MOI of 0.001, the OD_600_ values of all phages rapidly increased during the first two hours; thereafter, they steadily reached a growth plateau compared to the control group. Moreover, ΦL demonstrated enhanced efficacy; it inhibited bacterial growth even at the lowest phage concentration tested, with an MOI of 0.00001.

### 3.7. Effects of the Five Isolated STEC Phages on the Biofilm Prevention

**Figure 6:**
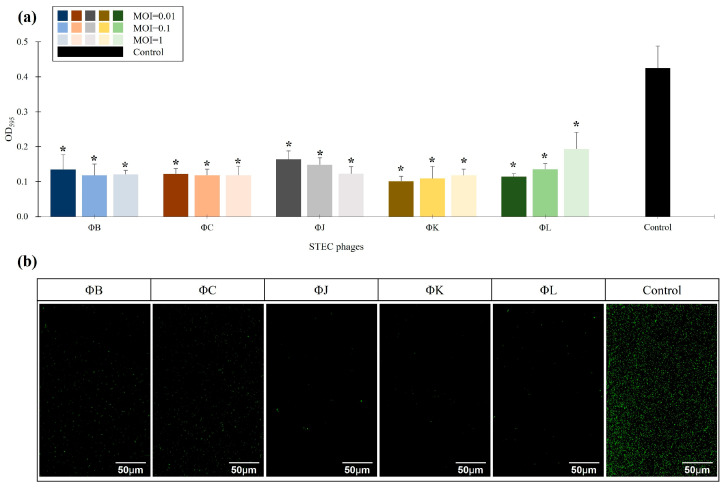
Effects of the five newly isolated STEC phages on biofilm inhibition. (**a**) Quantification of biofilm formation using crystal violet (CV) staining by measuring OD_595_. The control represents STEC ATCC 43895 without phage treatment. Data points represent the mean values of three independent experiments, and error bars indicate the standard deviations. Asterisks (*) indicate a statistically significant difference between the control and phage-treated groups (*p* < 0.05). (**b**) Confocal laser scanning microscopy (CLSM) images of biofilm inhibition. The biofilms were stained with SYTO 9 (green) to visualize live cells. Scale bars represent 50 µm.

The prevention effects of the five isolated STEC phages on STEC biofilms were evaluated using the strong biofilm-forming strain ATCC 43895 as a control (Figure 6). The biofilm mass in the phage-treated groups at MOIs of 0.01, 0.1, and 1 was quantified relative to the control using crystal violet (CV) staining and confocal laser scanning microscopy (CLSM) to determine the phage efficacy in biofilm inhibition. The relative biofilm mass was MOI-independent for all phages, with a reduction in biofilm prevention of approximately 70% across all phage concentrations, indicating that the phage concentrations were not significantly affected by MOI (Figure 6a). Among all phages, ΦK at an MOI of 0.01 was the most effective in preventing STEC biofilm formation, reaching a 78.2% reduction in biofilm mass compared to the control group. CLSM analysis revealed that all phages significantly inhibited biofilm formation at an MOI of 0.01, consistent with the reduction in biofilm observed in the CV staining results (Figure 6b).

### 3.8. Effects of the Five Isolated STEC Phages on the Pre-Formed Biofilm Eradication

**Figure 7:**
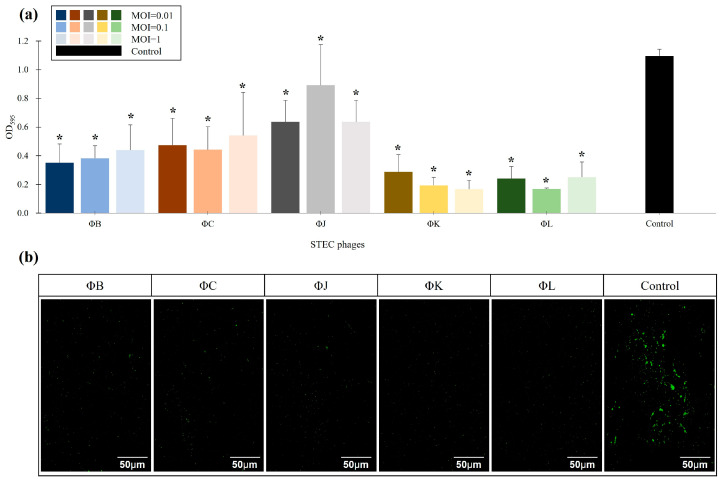
Effects of the five newly isolated STEC phages on biofilm eradication. (**a**) Quantification of biofilm formation using CV staining by measuring OD_595_. The control represents STEC ATCC 43895 without phage treatment. Data points represent the mean values of three independent experiments, and error bars indicate standard deviations. Asterisks (*) indicate a statistically significant difference between the control and phage-treated groups (*p* < 0.05). (**b**) CLSM images of biofilm eradication. The biofilms were stained with SYTO 9 (green) to visualize live cells. Scale bars represent 50 µm.

The eradication effects of the five isolated STEC phages on mature STEC biofilms were assessed using pre-formed biofilms of ATCC 43895 treated with phages at MOIs of 0.01, 0.1, and 1 (Figure 7). The biofilm mass in the phage-treated groups was quantified relative to the control using CV staining and CLSM analysis to determine phage efficacy in pre-formed biofilm inhibition. Relative biofilm mass was MOI-independent for all phages, with a reduction in pre-formed biofilms of approximately 62% across all phage concentrations. ΦK and ΦL exhibited the highest efficiency in biofilm eradication, reaching 80.3% and 73.9% reduction in biofilm mass compared to the control group, respectively (Figure 7a). CLSM analysis revealed that all phages at an MOI of 0.01 significantly inhibited mature biofilms, consistent with the reduction in biofilm mass observed in the CV staining results (Figure 7b).

### 3.9. General Characteristics of the Five Isolated STEC Phage Genomes

The whole genomes of the five isolated STEC phages comprised double-stranded DNA with a G+C content ranging from 37.6 to 37.7%. The genome sizes of the phages were 168,078 bp (ΦB), 168,095 bp (ΦC), 168,715 bp (ΦJ), 169,226 bp (ΦK), and 169,381 bp (ΦL). Functional annotation using a combination of BLASTp and InterPro databases predicted 265 (ΦB), 266 (ΦC, ΦJ, and ΦK), and 267 (ΦL) open reading frames (ORFs) in each genome (Figure S1). The predicted ORFs were classified into seven functional categories: lysis-related proteins, structural proteins, packaging proteins, DNA metabolism- and transcription-related proteins, putative depolymerase proteins, hypothetical proteins, and tRNAs (Table 3).

Comparative genomic analysis revealed that all five STEC phages belonged to the *Mosigvirus* genus based on the ICTV, exhibiting >79% nucleotide sequence identity with *Escherichia* phage T4 (NC_00866.4). Each phage genome contained 120–138 functionally annotated ORFs and two–three lysis-related proteins, including holins, lysozymes, and endolysins (Tables S1–S5). Putative depolymerase proteins were predicted using the DePP database. ΦB, ΦC, and ΦK each possessed two predicted depolymerases, whereas ΦJ and ΦL harbored one depolymerase each. All the predicted depolymerases were associated with tail fiber proteins, suggesting their potential for bacterial host recognition and infection. Further genomic analysis revealed two tRNAs in four phages (ΦB, ΦC, ΦJ, and ΦK). In contrast, ΦL contained 10 tRNAs. No host virulence factors or antimicrobial proteins were detected in the phage genomes. TMHMM-2.0 analysis identified 26–28 transmembrane proteins in all phages, primarily associated with baseplate wedge-related proteins, except for ΦK. Phage baseplate wedge proteins play major roles in bacterial host recognition, initial infection, and DNA injection. The absence of transmembrane domains in the baseplate wedge protein of ΦK suggests distinctiveness in its host determination, consistent with its lower host susceptibility observed in the host range analysis. Genomic similarity analysis based on the GenBank database revealed that each phage was most similar to specific members of the *Mosigvirus* genus. ΦB exhibited 96.2% identity with vB_EcoM_mar005P1 (LR027390); ΦC showed 96.6% identity with *Escherichia* phage FP43 (MN648445); and the remaining phages (ΦJ, ΦK, and ΦL) were most similar to *Escherichia* phage JN02 (MT782071), with identity ranging from 97.5% to 97.8%.

### 3.10. Phylogenetic and Comparative Genomic Analysis of the Five Isolated STEC Phages

**Figure 8:**
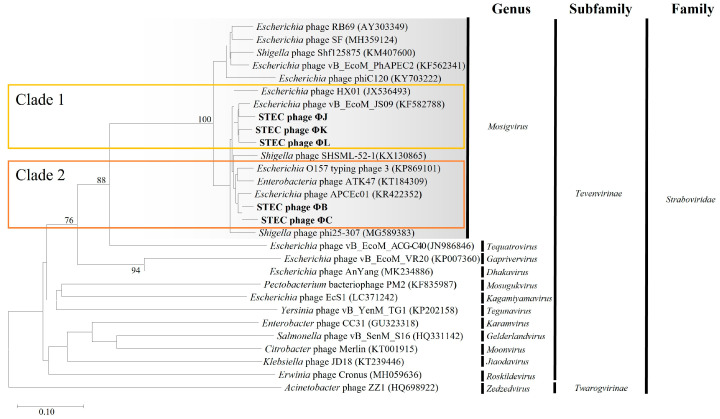
Phylogenetic analysis of the five newly isolated STEC phages based on whole-genome sequences. The Genome-BLAST distance phylogeny tree was constructed using the Virus Classification and Tree Building Online Resource (VICTOR) tool, comparing the genomes of the isolated phages (ΦB, ΦC, ΦJ, ΦK, and ΦL) with those of *Tevenvirinae* phages. The tree was generated using the D6 distance formula, with branch support values (%) calculated from 1000 bootstrap replicates. The average branch support across the entire tree was 22%.

**Figure 9:**
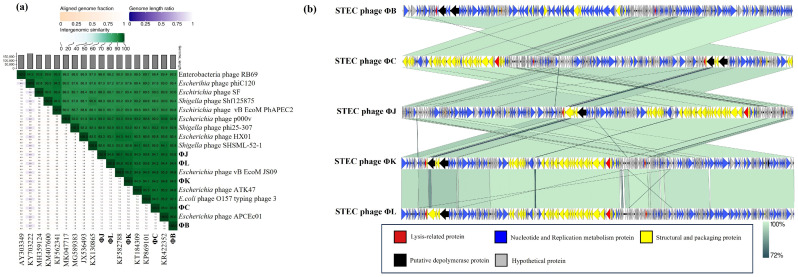
Comparative genomic analysis of the five newly isolated STEC phages and *Mosigvirus* phages available in the GenBank database. (**a**) A VIRIDIC-generated heatmap indicating the intergenomic similarities of the isolated phages (ΦB, ΦC, ΦJ, ΦK, and ΦL) with the representative *Mosigvirus*. The right side shows the intergenomic similarity between the genomes (gradient green), and the left side represents the aligned genomic fraction (gradient orange) and genome length ratio (gradient purple). (**b**) Genome alignment of the isolated phages generated using Easyfig. Different colored arrows indicate ORFs based on their putative predicted function. The grey bar shows similarities (%) in the phage homologous regions.

Comprehensive phylogenetic and comparative genomic analyses were performed to elucidate the genomic similarities among the five isolated STEC phages. A phylogenetic tree was constructed using the whole-genome sequences of the five isolated phages and 24 *Straboviridae* phages. The analysis included 12 members of the *Mosigvirus* genus (Table S6) and 11 phages from different genera within the *Tevenvirinae* subfamily. The resulting tree revealed that all five isolated phages clustered closely with the members of the *Mosigvirus* genus (Figure 8). To further support the phylogenetic analysis, the major capsid protein and terminase large subunit sequences were used to construct additional phylogenetic trees. These trees consistently showed that all the isolated phages clustered within the *Mosigvirus* genus (Figure S2). The Virus Intergenomic Distance Calculator (VIRIDIC)-generated heat map demonstrated high intergenomic similarities (>95%) between the isolated phages and representative *Mosigvirus* genomes (Figure 9). ΦB and ΦC exhibited an intergenomic similarity > 95% with the phage APCEc01, whereas ΦJ, ΦK, and ΦL displayed the highest similarity (>95%) with phage vB_EcoM_JS09. Genome structure comparisons revealed that the five phages shared high overall similarity but displayed significant differences in tail fiber proteins, which potentially encode depolymerase functions.

**Figure 10:**
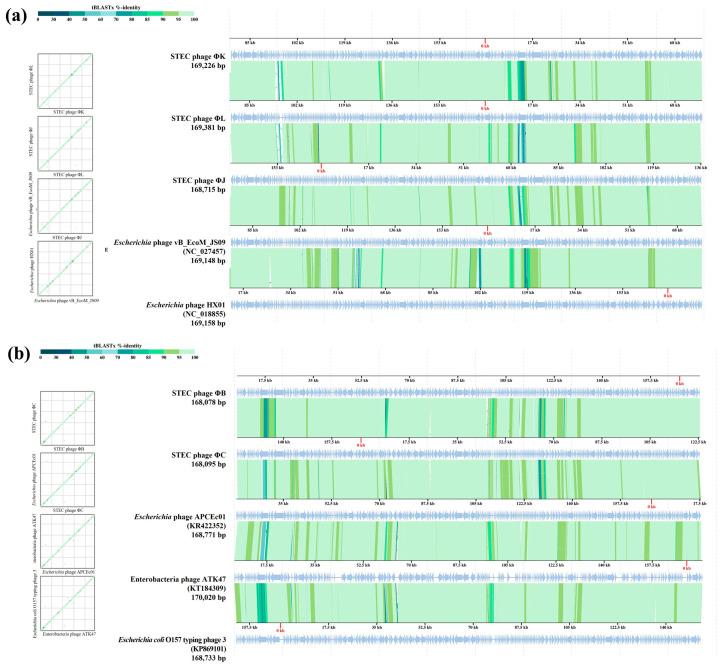
Comparative genome analysis of the five newly isolated STEC phages using VipTree. Two syntenic alignments of STEC phages ΦK, ΦL, and ΦJ, *Escherichia* phage HX01 (JX536493), and *Escherichia* phage vB_EcoM_JS09 (KF582788), belonging to clade 1 (**a**), and STEC phages ΦB and ΦC, *Escherichia* O157 typing phage 3 (KP869101), *Enterobacteria* phage ATK47 (KT184309), and *Escherichia* phage APCEc01 (KR422352), belonging to clade 2 (**b**), based on whole-genome phylogeny, respectively. The sequence positions are automatically optimized to represent collinearity between the genomes. Green-colored lines interconnecting the genomes indicate tBLASTx hits (e-value < 0.01) between protein-coding genes.

VipTree analysis was performed on two groups of the five phages, each based on their closest phylogenetic distances (Figure 10). Group 1, consisting of ΦB, ΦC, APCEc01, ATK47, and *E. coli* O157 typing phage 3, shared only approximately 70% similarity in the tail fiber protein (ORF28 in ΦB and ORF219 in ΦC). Other dissimilarities were also observed in DNA metabolism-related or hypothetical proteins. The phages APCEc01, ATK47, and *E. coli* O157 typing phage 3 showed identical ORF differences in the distal subunit of the long tail fiber. ΦB and ΦC exhibited a greater difference in the tail fiber protein sequence than the other three phages in the cluster, with the sequence difference exceeding 50% in certain regions, whereas the difference among the other phages was at least 78%. Group 2, comprising ΦK, ΦJ, ΦL, HX01, and JS09, showed consistent contrasting regions in the tail fiber protein. Similarities ranged from 79% to 89%, with ΦJ, ΦK, and ΦL displaying distinctive differences compared to the tail fiber differences between phages HX01 and JS09 (<70%). The observed differences in tail fiber proteins among the isolated phages and their closest relatives may contribute to their specific host ranges and antibiofilm activities.

## 4. Discussion

The emergence of MDR STEC strains presents a major public health challenge that necessitates the development of alternative therapeutic strategies. One of the main challenges in developing phage-based therapies against pathogenic bacteria is obtaining phages with sufficient host range for practical applications. The lytic activity of a single phage against bacterial strains generally varies considerably among species. In this study, we isolated and characterized five new phages belonging to the *Mosigvirus* genus. These phages exhibited potent lytic activity against MDR STEC strains and their biofilms. Our findings indicate that these phages are promising biocontrol agents with distinctive genomic features and broad-spectrum antimicrobial capabilities.

The AMR profiles revealed that all tested STEC strains were MDR, exhibiting resistance to at least one agent in three or more antimicrobial classes. High resistance rates were observed against β-lactam antibiotics (78.9–94.7%) and third-generation cephalosporins (78.9–84.2%), whereas moderate resistance to aminoglycosides (31.6–57.9%) was observed. These results indicate that clinically important antibiotics are becoming increasingly ineffective against STEC. Therefore, the development of alternative antimicrobial strategies, such as phage therapy, is urgently required. Carbapenems retained the highest efficacy, with only 11% resistance observed for imipenem. However, the emergence of meropenem-resistant strains (KCCM 90577, 90578, 90579, and 90580) indicates an emerging threat to this antibiotic class. Our isolated *Mosigvirus* phages demonstrated lytic activity independent of the bacterial antibiotic resistance profiles. All five phages successfully lysed STEC strains exhibiting high resistance to multiple antibiotic classes, including those resistant to clinically critical agents such as carbapenems and fluoroquinolones. This finding is particularly relevant, given that certain antibiotics can induce prophage activation and increase Stx production in STEC [10].

The morphological features of the five isolated phages revealed typical *Myoviridae* with T4-like morphology. In particular, ΦB exhibited a significantly smaller tail lengths (57.1 ± 2.2 nm) than those of the other four phages (109.1–123.7 nm), suggesting potential differences in genome packaging efficiency or structural organization. This morphological variation within the same genus has been previously observed in other T4-like phages, where tail length variations correlated with differences in DNA packaging mechanisms and genome size flexibility [26]. Classification analysis confirmed that all five phages belong to the *Mosigvirus* genus of the *Straboviridae* family, supporting the recent ICTV taxonomy for T4-like phages [29]. The broad host range exhibited by the isolated phages, particularly ΦL, which lysed 12 out of 19 STEC strains, demonstrates their potential as biocontrol agents against diverse STEC infections. The ability of all five phages to lyse *E. fergusonii* but not *E*. *hermanii* suggests specific receptor recognition patterns. Furthermore, the complete resistance observed in several STEC strains to all five phages, despite its STEC classification, suggests strain-specific variations in phage receptors or the presence of active phage defense mechanisms. The one-step growth curves revealed remarkably high replication efficiency, with all five phages exhibiting short latent periods of approximately 10 min and large burst sizes ranging from 78.7 (ΦK) to 427.4 PFU/cell (ΦJ), exceeding those reported for other characterized *Mosigvirus* members [51,52,53]. ΦK, which exhibited the narrowest host range, also had the smallest burst size, indicating that phages with broader host ranges demonstrate enhanced reproductive efficiency. The environmental stability of the five isolated phages demonstrated a pH range of 4–10 and complete viability at 40 °C. Contrastingly, ΦJ, ΦK, and ΦL had partial activity at 50 °C, exceeding that reported for many T4-like phages [54]. These stable conditions indicate their potential applications in certain food matrices and mildly acidic environments, where STEC proliferates [55]. All five isolated phages represented antibiofilm efficacy independent of MOI, achieving approximately 70% inhibition of biofilm formation and up to 80% disruption of established biofilms (ΦK). This MOI-independent antibiofilm activity, even at low phage concentrations, can effectively disrupt biofilm structures, which controls STEC for practical applications, where maintaining high phage titers may be challenging.

Genomic analysis of the five isolated phages revealed the absence of lysogeny-associated genes, virulence factors, or AMR determinants, confirming their suitability as safe biocontrol agents [56]. Several lysis-related proteins, including holins, lysozymes, and endolysins, were identified in each genome, indicating strict bacterial lysis mechanisms. Furthermore, tRNA gene content varied among the phage genomes: ΦB, ΦC, ΦJ, and ΦK each encode two tRNAs (methionine and arginine), whereas ΦL harbors 10 tRNAs. This enhanced tRNA complement likely contributes to a broader host range (ΦL, 12/19 strains infectivity), as phage-encoded tRNAs compensate for codon usage differences between phage and host genomes, thereby optimizing translational efficiency across phylogenetically diverse bacterial hosts [57,58,59]. Furthermore, the identification of 26–28 transmembrane proteins, primarily comprising baseplate wedge subunits, highlighted the specific host recognition of these phages. The absence of transmembrane domains in the baseplate wedge protein of ΦK coincides with its narrower host range, suggesting a structural feature as a determinant of host specificity [60]. Host range variation among these closely related phages may be due to differences in their receptor-binding proteins, particularly within tail fiber proteins [61]. All five phages possessed putative depolymerase-encoding genes associated with tail fiber proteins, conferring the capacity to recognize and bind to diverse bacterial surface receptors through enzymatic degradation of surface polysaccharides. These depolymerases serve dual functions: facilitating phage adsorption and disrupting biofilms by degrading extracellular polymeric matrix components. These mechanisms of bacterial lysis and biofilm matrix degradation, previously reported in other lytic phage systems [62,63], confer distinct advantages over antibiotics, which have reduced efficacy against biofilm-associated pathogens.

Phylogenetic analyses definitively revealed that all five phages belonged to the *Mosigvirus* genus of the *Straboviridae* family, which is consistent with the recent ICTV reclassification of T4-like phages. The clustering patterns in the whole-genome, major capsid protein, and terminase large subunit phylogenies simultaneously confirmed their taxonomic classification and delineated two distinct subgroups within the isolated phages. This taxonomic assignment was further supported by the presence of a conserved T4-like genomic organization in all five phage genomes. Despite the high overall sequence similarity (>95% within phylogenetic clusters), comparative genomic analysis revealed significant divergence in tail fiber protein sequences among the isolated phages. Amino acid similarities as low as 50% were detected in certain regions, even between phages within the same phylogenetic cluster. This sequence heterogeneity in tail fiber proteins determines host specificity through the differential recognition of distinct bacterial surface receptors, as previously reported for T4-like phages [64]. Targeted modifications of tail fiber proteins, particularly gp37, can affect host range [65,66,67].

Future studies should focus on characterizing the specific bacterial receptors targeted by the diverse tail fiber proteins of these *Mosigvirus* phages and evaluating their efficacy in more complex matrices, including food systems and in vivo infection models.

## 5. Conclusions

This study establishes the characterization of five new bacteriophages belonging to the *Mosigvirus* genus that exhibit potent bactericidal activity against MDR STEC strains. The isolated phages demonstrated broad host specificity and remarkable antibiofilm efficacy, indicating the critical limitations of current STEC control strategies. MOI-independent antibiofilm activity further enhances their practical applicability, where maintaining elevated phage densities proves challenging. Distinctive genomic features, particularly variant tail fiber protein sequences harboring putative depolymerases, coincided with a broader host range and enhanced biofilm disruption activities. Owing to their distinctive characteristics and proven efficacy against AMR strains, without triggering prophage-mediated toxin amplification, these phages represent strong candidates for biocontrol applications in both clinical and food safety systems. These findings establish the known diversity of *Mosigvirus* members that are effective against MDR STEC and provide valuable insights into their potential for managing persistent bacterial infections.

## Figures and Tables

**Table 1:** Antimicrobial susceptibility profiles of the 19 STEC strains used in this study.

Strains	Antimicrobial Agents (μg)																									
	PCN		BLC			CP							M	C		AM		MA	T		FQ					P
	AMP	PIP	AMC	SAM	TZP	FEP	CTX	CRO	CAZ	FOX	CXM	CFM	ATM	IPM	MEM	AMK	STR	AZM	TET	DOX	CIP	LVX	NAL	NOR	OFX	CHL
	(10)	(100)	(10/10)	(10/10)	(100/10)	(30)	(30)	(30)	(30)	(30)	(30)	(5)	(30)	(10)	(10)	(30)	(10)	(15)	(30)	(30)	(5)	(5)	(30)	(10)	(5)	(30)
ATCC 43895																										
KCCM 90572																										
KCCM 90573																										
KCCM 90574																										
KCCM 90575																										
KCCM 90576																										
KCCM 90577																										
KCCM 90578																										
KCCM 90579																										
KCCM 90580																										
KCCM 90581																										
KCCM 90582																										
KCCM 90583																										
KCCM 90584																										
KCCM 90585																										
KCCM 90586																										
KCCM 90587																										
NCCP 13720																										
NCCP 13721																										

The category of antibiotic susceptibility is indicated as follows: Dark gray, resistant; light gray, intermediate; white, susceptible. Antimicrobial concentrations (μg per disc) are shown below each agent name. PCN, Penicillin; AMP, Ampicillin; PIP, Piperacillin; BLC, β-lactam combination agents; AMC, Amoxicillin-clavulanate; SAM, Ampicillin-sulbactam; TZP, Piperacillin-tazobactam; CP, Cephalosporins; FEP, Cefepime; CTX, Cefotaxime; CRO, ceftriaxone; CAZ, Ceftazidime; FOX, Cefoxitin; CXM, Cefuroxime; CFM, Cefixime; M, Monobactam; ATM, Aztreonam; C, Carbapenem; IPM, Imipenem; MEM, Meropenem; AM, aminoglycosides; AMK, Amikacin; MA, Macrolides; STR, Streptomycin; AZM, Azithromycin; T, Tetracyclines; TET, Tetracycline; DOX, Doxycycline; FQ, Fluoroquinolones; CIP, Ciprofloxacin; LVX, Levofloxacin; NAL, Nalidixic acid; NOR, Norfloxacin; OFX, Ofloxacin; P, Phenicols; CHL, Chloramphenicol.

**Table 2:** Host ranges of the five isolated STEC phages.

Bacterial Species	Host Strain	STEC Phages					Sources
		ΦB	ΦC	ΦJ	ΦK	ΦL	
Shiga toxin-producing *E. coli* (STEC)	ATCC 43895	+	+	+	+	+	ATCC
	KCCM 90572	−	−	+	−	+	KCCM
	KCCM 90573	+	−	+	+	−	KCCM
	KCCM 90574	−	−	−	−	−	KCCM
	KCCM 90575	−	−	+	−	+	KCCM
	KCCM 90576	+	+	−	−	+	KCCM
	KCCM 90577	+	+	+	+	+	KCCM
	KCCM 90578	−	−	−	−	−	KCCM
	KCCM 90579	−	+	+	−	+	KCCM
	KCCM 90580	+	+	+	−	+	KCCM
	KCCM 90581	+	+	+	+	+	KCCM
	KCCM 90582	+	−	+	−	+	KCCM
	KCCM 90583	+	+	−	−	+	KCCM
	KCCM 90584	−	−	−	−	−	KCCM
	KCCM 90585	+	+	−	+	+	KCCM
	KCCM 90586	−	−	−	−	−	KCCM
	KCCM 90587	−	−	−	−	−	KCCM
	NCCP13720	−	−	−	−	−	NCCP
	NCCP13721	+	+	−	+	+	NCCP
Non-STEC	KCCM 90525	+	−	−	−	−	KCCM
	KCCM 90526	−	−	−	−	−	KCCM
	BL21	+	+	−	+	+	[50]
	ATCC 13706	−	+	+	+	+	ATCC
	ATCC 11775	−	−	+	+	+	ATCC
	ATCC 31616	−	−	−	−	−	ATCC
	ATCC 31618	+	+	+	−	−	ATCC
	ATCC 23545	+	+	+	+	+	ATCC
*E. fergusonii*	ATCC 35469	+	+	+	+	+	ATCC
*E. hermannii*	ATCC 33650	−	−	−	−	−	ATCC

+, phage infection (lysis observed); −, no phage infection (no lysis observed)

**Table 3:** Overview of biological and genomic features of the five isolated STEC phages.

Feature	STEC Phage				
	ΦB	ΦC	ΦJ	ΦK	ΦL
Biotype					
Morphology	*Myoviridae*	*Myoviridae*	*Myoviridae*	*Myoviridae*	*Myoviridae*
Head (nm)	107.5 ± 2.1	113.3 ± 3.4	112.7 ± 1.5	103.9 ± 2.6	108.2 ± 1.0
Tail (nm)	57.1 ± 2.2	112.1 ± 2.2	109.1 ± 3.19	123.7 ± 3.2	112.9 ± 6.1
Latent time (min)	10	10	10	10	10
Burst size (PFU/mL)	154.8	230.5	427.4	78.7	157.5
Optimal pH stability	4–10	4–10	4–10	4–10	4–10
Optimal thermal stability	0–40	0–40	0–50	0–50	0–50
Genotype					
Gnome size (bp)	168,078	168,095	168,715	169,226	169,381
G+C content (%)	37.6	37.6	37.7	37.6	37.6
Number of tRNAs	2	2	2	2	10
Number of CDS	265	266	266	267	267
Functional CDS	138	120	123	132	128
Accession number	OP114733	PQ308253	PQ308254	MZ868638	PQ308255

## Data Availability

The five STEC phages, designated as vB_EcoM-pJBB(ΦB), vB_EcoM-pJBC(ΦC), vB_EcoM-pJBJ(ΦJ), vB_EcoM-pJBK(ΦK), and vB_EcoM-pJBL(ΦL), were deposited in the Korean Collection for Type Cultures (KCTC) under KCTC 4826, KCTC 4827, KCTC 4829, KCTC 4830, and KCTC 4831, respectively. The complete genome sequences of these five STEC phages have been deposited in the GenBank database under the accession numbers OP114733, PQ308253, PQ308254, MZ868638, and PQ308255, respectively. The raw data supporting the conclusions of this article will be made available by the authors on request.

## Supplementary Materials

The following supporting information can be downloaded at: https://www.mdpi.com/article/10.3390/v17111501/s1, Figure S1: Circular genomic maps of the five newly isolated STEC phages ΦB (a), ΦC (b), ΦJ (c), ΦK (d), and ΦL (e) generated using CGView; Figure S2: Gene-based phylogenetic analysis of the five newly isolated STEC phages based on the amino acid sequences of the terminase large subunit (a) and major capsid protein (b); Table S1: Features of predicted open reading frames (ORFs) and their homology to STEC phage ΦB; Table S2: Features of predicted ORFs and their homology to STEC phage ΦC; Table S3: Features of predicted ORFs and their homology to STEC phage ΦJ; Table S4: Features of predicted ORFs and their homology to STEC phage ΦK; Table S5: Features of predicted ORFs and their homology to STEC phage ΦL; Table S6: Genomic features of the *Mosigvirus* genus, as determined based on data extracted from the GenBank database.

## Abbreviations

The following abbreviations are used in this manuscript:

STEC	Shiga toxin-producing *Escherichia coli*
Stx	Shiga toxin
AMR	antimicrobial resistance
ESBL	extended-spectrum beta-lactamase
MOI	multiplicity of infection
MDR	multidrug-resistant
Phage	Bacteriophage
CV	crystal violet
ΦB	vB_EcoM-pJBB
ΦC	vB_EcoM-pJBC
ΦJ	vB_EcoM-pJBJ
ΦK	vB_EcoM-pJBK
ΦL	vB_EcoM-pJBL
