# Supplementary material for: Characterization of Five Lytic Bacteriophages as New Members of the Genus Mosigvirus, Infecting Multidrug-Resistant Shiga Toxin-Producing Escherichia coli and Their Antibiofilm Activity
Source: Viruses. 2025 Nov 13;17(11):1501. doi: 10.3390/v17111501 (PMC12656860; doi:10.3390/v17111501)
Supplement: Supplementary file 1 [file viruses-17-01501-s001.zip › Figure S1,S2.pdf]

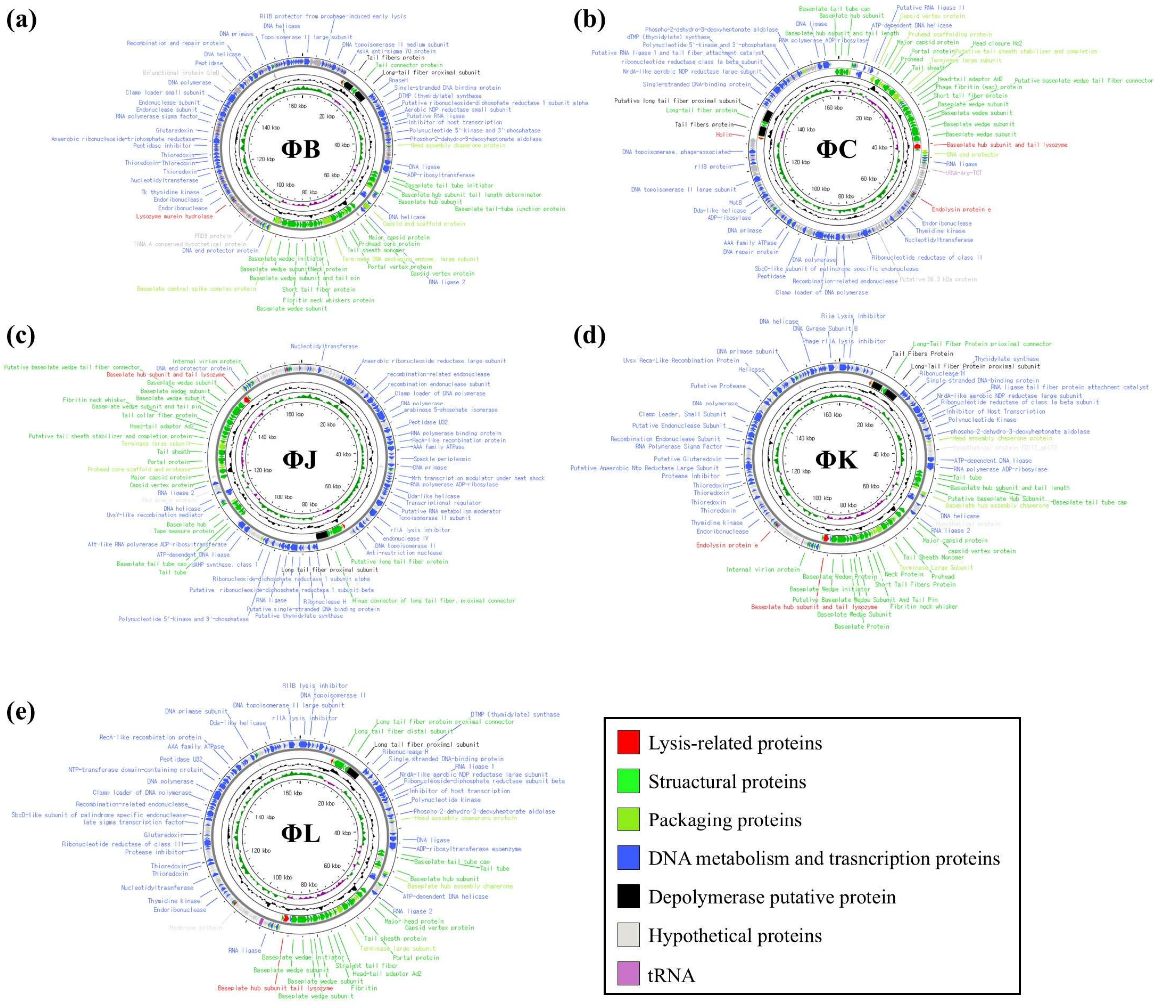

Figure S1. Circular genomic maps of the five newly isolated STEC phages ΦB (a), ΦC (b), ΦJ (c), ΦK (d), and ΦL (e) generated using CGView. The outermost ring represents the coding sequences on the forward strand, whereas the innermost ring shows those on the reverse strand. The second and third innermost rings display the GC content and GC skew, respectively. Each coding sequence is color-coded based on its predicted functional categories: lysis-related protein (red), structural-associated protein (green), virion packaging protein (light green), DNA metabolism and transcription protein (blue), putative depolymerase protein (black), and hypothetical protein (grey).

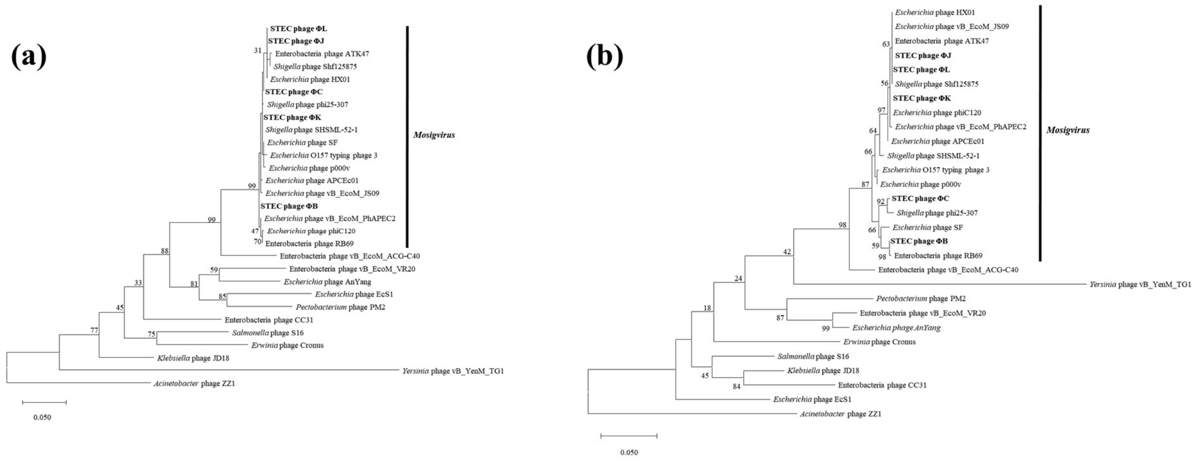

Figure S2. Gene-based phylogenetic analysis of the five newly isolated STEC phages based on the amino acid sequences of the terminase large subunit (a) and major capsid protein (b). Two phylogenies were constructed using the maximum likelihood method through MEGA 11 with 1000 bootstrap replicates. The bootstrap values are indicated at the internal nodes of the trees.
