# Supplementary material for: Characterization of Five Lytic Bacteriophages as New Members of the Genus Mosigvirus, Infecting Multidrug-Resistant Shiga Toxin-Producing Escherichia coli and Their Antibiofilm Activity
Source: Viruses. 2025 Nov 13;17(11):1501. doi: 10.3390/v17111501 (PMC12656860; doi:10.3390/v17111501)
Supplement: Supplementary file 1 [file viruses-17-01501-s001.zip › Table S1.pdf]

Table S1. Features of predicted open reading frames (ORFs) and their homology to STEC phage ΦB.

Gene product				Putative function [Conserved domain]	Best match organism (E-value)	Identity (%)	Predicted TMHHM and signal peptide	
ORF No.	Range	Strand	Length (AA)				TMHHM	SignalP
1	2-67	+	21	Hypothetical protein	*Escherichia* phage vB_EcoM_NBG1 (5e-12)	100	0	N
2	70-495	+	141	Hypothetical protein [PF03961; FapA; Flagellar Assembly Protein A]	*Escherichia* phage ST0 (8e-96)	100	0	N
3	559-2376	+	605	Topoisomerase II large subunit [PF02518; HATPase_c; Histidine kinase-, DNA gyrase B-, and HSP90-like ATPase]	*Escherichia* phage vB_EcoM_PhAPEC2 (0.0)	99.8	0	N
4	2419-3519	+	366	Hypothetical protein	*Escherichia* phage phiE142 (0.0)	98.9	0	N
5	3612-3812	+	66	Hypothetical protein	*Escherichia* phage RB69 (4e-37)	100	0	N
6	3825-6038	+	737	Hypothetical protein	Escherichia phage p000v (0.0)	99.6	0	N
7	6048-6983	+	311	RIIB protector from prophage-induced early lysis [PF13384; HTH_23; Homeodomain-like domain]	*Escherichia* phage F2 (0.0)	99.3	0	N
8	7024-7311	+	95	Hypothetical protein	*Escherichia* phage RB69 (2e-61)	98.9	0	N
9	7328-7804	+	158	DNA endonuclease IV	*Escherichia* phage JN02 (6e-113)	99.4	0	N
10	7873-8136	+	87	Hypothetical protein	*Escherichia* phage vB_EcoM_G53 (8e-57)	98.8	0	N
11	8216-8326	+	36	Hypothetical protein	*Escherichia* phage vB_EcoM_JS09 (1e-16)	100	1	N
12	8387-8587	+	66	Hypothetical protein	*Escherichia* phage vB_EcoM_JS09 (3e-41)	100	0	N
13	8664-9110	+	148	Nucleoid disruption protein [PF06591; Phage_T4_Ndd; T4-like phage nuclear disruption protein]	*Escherichia* phage ST0 (4e-104)	99.3	0	N
14	9163-9309	+	48	Hypothetical protein [PF06341; DUF1056; Protein of unknown function]	*Escherichia* phage p000y (1e-23)	100	1	N

15	9309-9449	+	46	Hypothetical protein [PF01102; Glycophorin_A; Glycophorin A]	*Escherichia* phage HP3 (4e-20)	97.8	1	N
16	9454-10779	+	441	DNA topoisomerase II medium subunit [PF00521; DNA_topoisoIV; DNA gyrase/topoisomerase IV, subunit A]	*Escherichia* phage vB_EcoM_JS09 (0.0)	99.5	0	N
17	10965-11180	+	71	Hypothetical protein [PF14574; RACo_C_ter; C-terminal domain of RACo the ASKHA domain]	*Escherichia* phage vB_EcoM_JS09 (3e-42)	100	0	N
18	11285-11917	+	210	Activator of middle period transcription [PF09114; MotA_activ; Transcription factor MotA, activation domain]	*Escherichia* phage vB_EcoM_JS09 (6e-147)	99.5	0	N
19	11928-12257	+	109	Hypothetical protein	*Escherichia* phage APCEc01 (5e-74)	99.1	0	N
20	12254-12715	+	153	Hypothetical protein	*Escherichia* phage vB_EcoM_JS09 (9e-110)	100	0	N
21	12715-12996	+	93	Anti-restriction nuclease	*Shigella* phage SHSML-52-1 (3e-60)	97.8	0	N
22	13173-13292	+	39	Hypothetical protein	*Escherichia* phage vB_EcoM_JS09 (1e-17)	100	0	N
23	13282-13581	+	99	Hypothetical protein [PF09063; Phage_coat; Phage PP7 coat protein]	*Escherichia* phage OLB35 (2e-63)	97	0	N
24	13571-13732	+	53	Hypothetical protein [PF04844; Ovate; Transcriptional repressor, ovate]	*Escherichia* phage vB_EcoM_G2469 (3e-27)	96.2	0	N
25	13779-14051	+	90	AsiA anti-sigma 70 protein [PF09010; AsiA; Anti-Sigma Factor A]	*Escherichia* phage RB69 (1e-54)	97.8	0	N
26	14052-14711	-	219	Holin [PF11031; Phage_holin_T; Bacteriophage T holin]	*Escherichia* phage p000v (3e-159)	99.1	1	N
27	14721-15272	-	183	Tail fibers protein [PF02413; Caudo_TAP; Caudovirales tail fiber assembly protein, lambda gpK]	*Escherichia* phage vB_EcoM_JS09 (2e-126)	97.3	0	N
28	15303-18491	-	1026	Tail fibers protein [PF07484; Collar; Phage Tail Collar Domain]	*Escherichia* phage mogra (0.0)	81.6	0	N
29	18500-19159	-	219	Gp36 hinge connector of long tail fiber, distal connector [PF03903; Phage_T4_gp36; Phage T4 tail fiber]	*Escherichia* phage T2 (2e-137)	91.8	0	N
30	19222-20349	-	375	Tail connector protein [PF15711; ILEI; Interleukin-like EMT inducer]	*Escherichia* phage ST0(0.0)	98.7	0	N

31	20358-24233	-	1291	Long-tail fiber proximal subunit [PF14366; DUF4410; Domain of unknown function]	*Escherichia* phage S143_2 (0.0)	97.5	0	N
32	24337-25254	+	305	RnaseH [PF09293; RNaseH_C; T4 RNase H, C terminal]	*Escherichia* phage ST0 (0.0)	100	0	N
33	25262-25531	+	89	Double-stranded DNA binding protein [PF11126; Phage_DsbA; Transcriptional regulator DsbA]	*Escherichia* phage RB69 (3e-56)	100	0	N
34	25509-25847	+	112	Late promoter transcription accessory protein [PF16805; Trans_coact; Phage late-transcription coactivator]	*Escherichia* phage vB_EcoM_PhAPEC2 (8e-75)	100	0	N
35	25844-26497	+	217	Helicase loading protein [PF08993; T4_Gp59_N; T4 gene Gp59 loader of gp41 DNA helicase]	*Shigella* phage JK45 (1e-156)	100	0	N
36	26612-27511	+	299	Single-stranded DNA binding protein [PF08804; gp32; gp32 DNA binding protein like]	*Escherichia* phage F2 (0.0)	100	0	N
37	27625-28017	+	130	FRD2 protein [PF03197; FRD2; Bacteriophage FRD2 protein]	*Escherichia* phage vB_EcoM_KAW3E185 (1e-85)	96.2	0	N
38	28079-28327	+	82	Hypothetical protein [PF17438; DUF5417; Family of unknown function]	*Escherichia* phage vB_EcoM_G53 (4e-51)	96.3	0	N
39	28330-28917	+	195	Dihydrofolate reductase [PF00186; DHFR_1; Dihydrofolate reductase]	*Escherichia* phage p000v (1e-140)	97.9	0	N
40	28914-29774	+	286	DTMP (thymidylate) synthase [PF00303; Thymidylat_synt; Thymidylate synthase]	*Escherichia* virus vB_Eco_mar005P1 (0.0)	99.7	0	N
41	29775-30029	+	84	Hypothetical protein [PF17600; DUF5496; Family of unknown function]	*Escherichia* phage vB_EcoM_G2469 (1e-52)	97.6	0	N
42	30117-32372	+	751	Putative ribonucleoside-diphosphate reductase 1 subunit alpha [PF02867; Ribonuc_red_lgC; Ribonucleotide reductase, barrel domain]	*Escherichia* phage vB_EcoM_WFbE185 (0.0)	100	0	N
43	32428-33606	+	392	Aerobic NDP reductase small subunit [PF00268; Ribonuc_red_sm; Ribonucleotide reductase, small chain]	*Escherichia* phage OLB35 (0.0)	99.7	0	N
44	33633-34043	+	136	Endonuclease II [PF01541; GIY-YIG; GIY-YIG catalytic domain]	*Escherichia* phage vB_EcoM_JS09 (5e-95)	100	0	N
45	34099-35223	+	374	Putative RNA ligase [PF09511; RNA_lig_T4_1; RNA ligase]	*Escherichia* phage vB_EcoM_MM02 (0.0)	99.5	0	N
46	35286-35786	+	166	Inhibitor of host transcription [PF17527; ALC; Phage ALC protein]	*Escherichia* phage mobillu (4e-118)	98.8	0	N

47	35774-36130	+	118	Spanin Rz [PF06810; Phage_GP20; Phage minor structural protein GP20]	*Escherichia* phage mogra (2e-75)	98.3	1	N
48	36127-36417	+	96	Hypothetical protein	*Escherichia* phage vB_EcoM_G2285 (2e-63)	97.9	0	Y
49	36414-36632	+	72	Hypothetical protein	*Escherichia* phage HP3 (5e-45)	100	0	N
50	36695-36994	+	99	Hypothetical protein [PF05714; PFam54_60; Borrelia Bbcrasp-1 domain containing protein]	*Escherichia* phage phiC120 (5e-35)	66.7	0	N
51	36994-37185	+	63	Hypothetical protein	*Escherichia* phage RB69 (8e-38)	100	0	N
52	37182-38081	+	299	Polynucleotide 5'-kinase and 3'-phosphatase [PF13671; AAA_33; AAA domain]	*Escherichia* phage mobillu (0.0)	100	0	N
53	38082-38273	+	63	Hypothetical protein	*Escherichia* phage ST0 (1e-36)	98.4	0	N
54	38263-38478	+	71	Hypothetical protein	*Escherichia* phage RB69 (2e-44)	100	0	N
55	38486-38761	+	91	Hypothetical protein	*Escherichia* phage RB69 (1e-58)	100	0	N
56	38822-39058	+	78	Hypothetical protein	*Escherichia* phage HX01 (2e-49)	100	0	N
57	39045-39170	+	41	Hypothetical protein	*Escherichia coli* O157 typing phage 3 (2e-19)	100	0	N
58	39179-40171	+	330	Phospho-2-dehydro-3-deoxyheptonate aldolase [PF00793; DAHP_synth_1; DAHP synthetase I family]	*Escherichia* phage OLB35 (0.0)	99.4	0	N
59	40171-40752	+	193	Deoxycytidylate deaminase [PF00383; dCMP_cyt_deam_1; Cytidine and deoxycytidylate deaminase zinc-binding region]	*Escherichia* phage vB_EcoM_PhAPEC2 (3e-141)	100	0	N
60	40754-41050	+	98	Hypothetical protein [PF10902; WYL_2; WYL_2, Sm-like SH3 beta-barrel fold]	*Escherichia* phage RB69 (4e-64)	98	0	N
61	41108-41440	+	110	Head assembly chaperone protein [PF00166; Cpn10; Chaperonin 10 Kd subunit]	*Escherichia* phage vB_EcoM_NBG1 (1e-71)	100	0	N
62	41565-41813	+	82	Lysis inhibition accessory protein [PF07377; DUF1493; Protein of unknown function]	*Escherichia coli* bacteriophage APCEc01 (1e-50)	98.7	0	N

63	42108-42287	+	59	Hypothetical protein	*Escherichia* phage RB69 (9e-32)	100	0	N
64	42418-42786	+	122	Hypothetical protein [PF06019; Phage_30_8; Phage GP30.8 protein]	*Escherichia* phage vB_EcoM_JS09 (7e-85)	100	0	N
65	42861-43226	+	121	Hypothetical protein [PF06919; Phage_T4_Gp30_7; Phage Gp30.7 protein]	*Escherichia* phage APCEc01 (7e-86)	100	0	N
66	43262-43876	+	204	Hypothetical protein	*Escherichia* phage APCEc01 (2e-150)	99.5	0	N
67	43931-44128	+	65	Hypothetical protein	*Escherichia* phage APCEc01 (4e-39)	100	0	N
68	44118-44336	+	72	Hypothetical protein	*Escherichia* phage APCEc01 (1e-45)	100	0	N
69	44329-44787	+	152	Hypothetical protein [PF08010; Phage_30_3; Bacteriophage protein GP30.3]	*Escherichia coli* O157 typing phage 3 (1e-108)	100	0	N
70	44784-45608	+	274	Hypothetical protein [PF13419; HAD_2; Haloacid dehalogenase-like hydrolase]	*Escherichia* phage APCEc01 (0.0)	99.2	0	N
71	45618-45887	+	89	Hypothetical protein [PF11243; DUF3045; Protein of unknown function]	*Escherichia* phage HX01 (4e-59)	100	0	N
72	45884-47377	+	497	DNA ligase [PF01068; DNA_ligase_A_M; ATP dependent DNA ligase domain]	*Escherichia* phage APCEc01 (0.0)	100	0	N
73	47377-47565	+	62	Hypothetical protein	*Escherichia* phage p000v (5e-37)	96.7	0	N
74	47621-49708	+	695	ADP-ribosyltransferase [PF03496; ADPrib_exo_Tox; ADP-ribosyltransferase exoenzyme]	*Escherichia* phage APCEc01 (0.0)	99.2	0	N
75	49767-50060	+	97	Hypothetical protein [PF17602; DUF5498; Family of unknown function]	*Escherichia* phage APCEc01 (6e-64)	100	0	N
76	50093-51055	-	320	Baseplate tail tube initiator [PF06841; Phage_T4_gp19; T4-like virus tail tube protein gp19]	*Escherichia* phage vB_EcoM_PhAPEC2 (0.0)	100	0	N
77	51055-52164	-	369	Baseplate tail-tube junction protein [PF11091; T4_tail_cap; Tail-tube assembly protein]	*Escherichia* phage RB69 (0.0)	100	0	N
78	52173-53945	-	590	Baseplate hub subunit tail length determinator [PF18567; TIR_3; Toll/interleukin-1 receptor domain]	*Escherichia* phage APCEc01 (0.0)	99.8	0	N
79	53942-54412	-	156	Baseplate hub assembly protein [PF11110; Phage_hub_GP28; Baseplate hub distal subunit]	*Escherichia* phage PTK (1e-110)	100	0	N

80	54423-55595	-	390	Baseplate hub subunit [PF09097; Phage-tail_1; Baseplate structural protein, domain 1]	*Escherichia* phage vB_EcoM_NBG1 (0.0)	100	0	N
81	55592-56344	-	250	Baseplate hub assembly protein [PF12322; T4_baseplate; T4 bacteriophage base plate protein]	*Escherichia* phage vB_EcoM_JS09 (0.0)	100	0	N
82	56392-57018	+	208	Bp26 baseplate hub subunit [PF12322; T4_baseplate; T4 bacteriophage base plate protein]	*Escherichia* phage RB69 (1e-153)	100	0	N
83	57018-57416	+	132	Baseplate wedge subunit [PF04965; GPW_gp25; Baseplate wedge protein gp25]	*Escherichia* phage RB69 (2e-90)	100	0	N
84	57496-57909	+	137	Recombination, repair and ssDNA binding protein [PF11056; UvsY; Recombination, repair and ssDNA binding protein UvsY]	*Escherichia* phage APCEc01 (2e-94)	100	0	N
85	57909-58133	+	74	Hypothetical protein [PF17834; GHD; Beta-sandwich domain in beta galactosidase]	*Escherichia* phage APCEc01 (1e-46)	100	0	N
86	58166-58333	+	55	Hypothetical protein [PF10886; DUF2685; Protein of unknown function]	*Escherichia* phage RB69 (1e-31)	100	0	N
87	58394-58624	-	76	ATP-dependent DNA helicase [PF11637; UvsW-1; UvsW.1 domain]	*Shigella* phage JK45 (3e-43)	98.7	0	N
88	58650-60164	-	504	DNA helicase [PF00271; Helicase_C; Helicase conserved C-terminal domain]	*Escherichia* phage moha (0.0)	99.6	0	N
89	60215-60883	+	222	Inhibitor of prohead protease [PF00595; PDZ; PDZ domain]	*Escherichia* phage moskry (1e-157)	99.5	0	N
90	60893-62311	+	472	Capsid and scaffold protein [PF00801; PKD; PKD domain]	*Escherichia* phage vB_EcoM_FT (0.0)	87.7	0	N
91	62414-62608	+	64	Hypothetical protein [PF11242; DUF2774; Protein of unknown function]	*Escherichia* phage RB69 (9e-37)	100	0	N
92	62605-62856	+	83	Hypothetical protein [PF09286; Pro-kuma_activ; Pro-kumamolisin, activation domain]	*Escherichia* phage vB_EcoM_JS09 (2e-55)	100	0	N
93	62976-63974	+	332	RNA ligase 2 [PF09414; RNA_ligase; RNA ligase]	*Escherichia* phage p000y (0.0)	100	0	N
94	64005-65288	-	427	Capsid vertex protein [PF07068; Gp23; Major capsid protein Gp23]	*Escherichia* phage vB_EcoM_G53 (0.0)	100	0	N
95	65390-65659	+	89	Hypothetical protein [PF16151; DUF4859; Domain of unknown function]	*Escherichia* coli O157 typing phage 3 (1e-57)	100	0	N

96	65712-67280	-	522	Major capsid protein [PF07068; Gp23; Major capsid protein Gp23]	*Escherichia* phage RB69 (0.0)	99.8	0	N
97	67298-68110	-	270	Prohead core protein [PF04344; CheZ; Chemotaxis phosphatase, CheZ]	*Escherichia* phage vB_EcoM_JS09 (0.0)	100	0	N
98	68144-68785	-	213	Prohead core scaffolding protein and protease [PF03420; Peptidase_S77; Prohead core protein serine protease]	*Escherichia* phage RB69 (1e-152)	100	0	N
99	68785-69210	-	141	Prohead core protein [PF18097; Vta1_C; Vta1 C-terminal domain]	*Escherichia* phage RB69 (1e-95)	100	0	N
100	69210-69440	-	76	Prohead core protein [PF17634; GP67; Gene product 67]	*Escherichia* phage moha (2e-40)	98.7	0	N
101	69440-71011	-	523	Portal vertex protein [PF07230; Portal_Gp20; Bacteriophage T4-like portal protein]	*Escherichia* phage APCEc01 (0.0)	100	0	N
102	71096-71587	-	163	Tail tube protein [PF06841; Phage_T4_gp19; T4-like virus tail tube protein gp19]	*Escherichia* phage RB69 (3e-116)	100	0	N
103	71700-73682	-	660	Tail sheath monomer [PF04984; Phage_sheath_1; Phage tail sheath protein subtilisin-like domain]	*Escherichia* phage mogra (0.0)	99.8	0	N
104	73713-75548	-	611	Terminase DNA packaging enzyme, large subunit [PF03237; Terminase_6N; Terminase large subunit, T4likevirus-type, N-terminal]	*Escherichia* phage SF (0.0)	99.8	0	N
105	75532-76026	-	164	Terminase DNA packaging enzyme, small subunit [PF11053; DNA_Packaging; Terminase DNA packaging enzyme]	*Escherichia* phage vB_EcoM_PhAPEC2 (9e-117)	99.3	0	N
106	76036-76857	-	273	Tail sheath stabilizer and completion protein [PF16724; T4-gp15_tss; T4-like virus Myoviridae tail sheath stabiliser]	*Escherichia* phage vB_EcoM_PhAPEC2 (0.0)	100	0	N
107	76910-77674	-	254	Neck protein [PF11649; T4_neck-protein; Virus neck protein]	*Escherichia* phage RB69 (0.0)	99.6	0	N
108	77676-78602	-	308	Neck protein [PF18148; RGS_DHEX; Regulator of G-protein signalling DHEX domain]	*Escherichia* phage vB_EcoM_JS09 (0.0)	100	0	N
109	78635-80083	-	482	Fibritin neck whiskers protein [PF07921; Fibritin_C; Fibritin C-terminal region]	*Escherichia* phage moskry (0.0)	99.1	0	N
110	80083-81666	-	527	Short tail fiber protein [PF14928; S_tail_recep_bd; Short tail fiber protein receptor-binding domain]	*Escherichia* phage S143_2 (0.0)	99	0	N
111	81663-82322	-	219	Baseplate wedge subunit and tail pin [PF08677; GP11; GP11 baseplate wedge protein]	*Escherichia* phage F2 (2e-158)	99	0	N

112	82322-84127	-	601	Baseplate wedge subunit and tail pin [PF07880; T4_gp9_10; Bacteriophage T4 gp9/10-like protein]	*Escherichia* phage vB_EcoM_G2469 (0.0)	99.8	0	N
113	84127-84999	-	290	Baseplate wedge tail fiber connector [PF07880; T4_gp9_10; Bacteriophage T4 gp9/10-like protein]	*Escherichia* phage vB_EcoM_NBG1 (0.0)	100	0	N
114	85072-86076	-	334	Baseplate wedge subunit [PF09215; Phage-Gp8; Bacteriophage T4, Gp8]	*Escherichia* phage vB_EcoM_JS09 (0.0)	100	0	N
115	86069-89167	-	1032	Baseplate wedge initiator [PF04353; Rsd_AlgQ; Regulator of RNA polymerase sigma(70) subunit, Rsd/AlgQ]	*Escherichia* phage moskry (0.0)	99.7	1	N
116	89164-91137	-	657	Baseplate wedge subunit	*Escherichia* phage mobillu (0.0)	99.7	0	N
117	91146-91439	-	97	Hypothetical protein [PF05488; PAAR_motif; PAAR motif]	*Escherichia* phage p000y (3e-64)	100	0	N
118	91442-91915	-	157	Hypothetical protein	*Escherichia* coli O157 typing phage 3 (2e-110)	98.7	0	N
119	91961-93694	-	577	Baseplate central spike complex protein	*Escherichia* phage S143_2 (0.0)	99.6	0	N
120	93694-94269	+	191	Baseplate wedge subunit [PF11246; Phage_gp53; Base plate wedge protein 53]	*Escherichia* phage APCEc01 (2e-137)	99.4	0	N
121	94331-94780	+	149	Head completion protein [PF08722; Tn7_Tnp_TnsA_N; TnsA endonuclease N terminal]	*Escherichia* phage vB_EcoM_JS09 (7e-106)	100	0	N
122	94783-95604	+	273	DNA end protector protein [PF13422; DUF4110; Domain of unknown function]	*Escherichia* phage mobillu (0.0)	99.6	0	N
123	95707-96291	+	194	Tail completion and sheath stabilizer protein [PF06841; Phage_T4_gp19; T4-like virus tail tube protein gp19]	*Escherichia* phage vB_EcoM_PhAPEC2 (1e-142)	100	0	N
124	96345-97079	+	244	Deoxynucleoside monophosphate kinase [PF04275; P-mevalo_kinase; Phosphomevalonate kinase]	*Escherichia* phage APCEc01 (4e-178)	100	0	N
125	97084-97314	+	76	Chaperone for tail fiber formation [PF17594; GP57; Phage Tail fiber assembly helper gene product 57]	*Escherichia* phage APCEc01 (4e-42)	98.6	0	N
126	97314-97769	+	151	RNA_lig [PF13563; 2_5_RNA_ligase2; 2'-5' RNA ligase superfamily]	*Escherichia* phage vB_EcoM_JS09 (5e-107)	98.6	0	N
127	97850-98161	+	103	Hypothetical protein [PF13732; DUF4162; Domain of unknown function]	*Escherichia* phage p000v (2e-67)	100	0	N

128	98223-98486	+	87	Hypothetical protein	*Escherichia* phage vB_EcoM_G2469 (6e-56)	98.8	0	N
129	98556-98741	+	61	TRNA.4 conserved hypothetical protein [PF17776; NLRC4_HD2; NLRC4 helical domain HD2]	*Escherichia* phage RB69 (3e-33)	100	2	N
130	98743-99105	+	120	Hypothetical protein	*Escherichia* phage vB_EcoM_PhAPEC2 (1e-84)	100	0	N
131	99102-99392	+	96	Hypothetical protein [PF03906; Phage_T7_tail; Phage T7 tail fiber protein]	*Escherichia* phage vB_EcoM_WFbE185 (5e-62)	96.8	0	N
132	99397-99912	+	171	Hypothetical protein	*Escherichia* phage phiC120 (3e-116)	97.6	0	N
133	100167-100511	+	114	Hypothetical protein [PF10849; DUF2654; Protein of unknown function]	*Escherichia* phage S14 (3e-77)	97.3	0	N
134	100898-101362	+	154	Hypothetical protein [PF06252; DUF1018; Protein of unknown function]	*Escherichia* phage SF (1e-105)	99.3	0	N
135	101486-101791	+	101	Hypothetical protein	*Escherichia* phage HP3 (3e-67)	99.0	0	N
136	101860-102024	+	54	Hypothetical protein	*Escherichia* phage RB69 (8e-31)	100	0	N
137	102071-102298	+	75	FRD3 protein [PF05798; Phage_FRD3; Bacteriophage FRD3 protein]	*Escherichia* phage vB_EcoM_G2285 (1e-47)	98.6	0	N
138	102369-102962	+	197	Hypothetical protein [PF08644; SPT16; FACT complex subunit (SPT16/CDC68)]	*Escherichia* phage RB69 (3e-135)	99.4	0	N
139	103012-103614	+	200	Hypothetical protein	*Escherichia* phage RB69 (5e-147)	99.5	0	N
140	103604-103978	+	124	Hypothetical protein [PF10669; Phage_Gp23; Protein gp23 (Bacteriophage A118)]	*Escherichia* phage Moha (4e-82)	95.1	2	N
141	103957-104316	+	119	Hypothetical protein [PF00584; SecE; ecE/Sec61-gamma subunits of protein translocation complex]	*Escherichia* phage vB_EcoM_MM02 (9e-83)	100	2	N
142	104313-104618	+	101	Hypothetical protein [PF14550; Peptidase_S78_2; Putative phage serine protease XkdF]	*Escherichia* phage vB_EcoM_JS09 (8e-67)	100	0	N
143	104628-104900	+	90	Hypothetical protein	*Escherichia* phage vB_EcoM_G2469 (4e-60)	100	0	N
144	104910-105107	+	65	Hypothetical protein	*Escherichia* phage vB_EcoM_PhAPEC2 (1e-38)	100	0	N

145	105170-105409	+	79	Hypothetical protein	*Escherichia* phage RB69 (8e-51)	98.7	0	N
146	105438-106394	+	318	Hypothetical protein	*Escherichia* phage APCEc01 (0.0)	98.7	0	N
147	106465-106770	+	101	Hypothetical protein	*Escherichia* phage APCEc01 (1e-67)	100	2	N
148	106772-107458	+	228	Hypothetical protein	*Escherichia* phage APCEc01 (1e-168)	99.1	0	N
149	107458-107949	+	163	Hypothetical protein [PF03616; Glt_symporter; Sodium/glutamate symporter]	*Escherichia* phage vB_EcoM_PhAPEC2 (3e-112)	98.1	2	N
150	107946-108182	+	78	Hypothetical protein [PF16152; DUF4860; Domain of unknown function]	*Escherichia* phage moha (8e-47)	98.7	0	N
151	108175-108630	+	151	Hydrolase [PF00293; NUDIX; NUDIX domain]	*Escherichia* phage ST0 (9e-109)	98.6	0	N
152	108665-109153	+	162	Lysozyme murein hydrolase [PF00959; Phage lysozyme; Phage lysozyme]	*Escherichia* phage phiE142 (2e-116)	99.3	0	N
153	109150-109431	+	93	Putative internal head protein	*Escherichia* phage moha (1e-57)	98.9	0	N
154	109490-109903	+	137	DenV endonuclease V, N-glycosylase UV repair enzyme [PF03013; Pyr_excise; Pyrimidine dimer DNA glycosylase]	*Escherichia* phage RB69 (8e-97)	99.2	0	N
155	109917-110171	+	84	Hypothetical protein	*Escherichia* phage vB_EcoM_WFbE185 (9e-50)	97.5	0	N
156	110236-110550	+	104	Hypothetical protein	*Escherichia* phage vB_EcoM_JS09 (2e-70)	100	0	N
157	110576-110905	+	109	Hypothetical protein [PF07453; NUMOD1; NUMOD1 domain]	*Escherichia* phage APCEc01 (3e-72)	100	0	N
158	111078-111617	+	179	Endoribonuclease [PF03237; Terminase_6N; Terminase large subunit, T4likevirus-type, N-terminal]	*Escherichia* phage moha (1e-130)	99.4	0	Y
159	111614-111922	+	102	Endoribonuclease	*Escherichia* coli O157 typing phage 3 (3e-70)	99	0	N
160	111929-112291	+	120	Autonomous glycyl radical cofactor [PF01228; Gly_radical; Glycine radical]	*Escherichia* phage mogra (7e-82)	99.1	0	N

161	112291-112515	+	74	Hypothetical protein	*Escherichia* phage RB69 (3e-46)	98.6	0	N
162	112505-112771	+	88	Hypothetical protein [PF04931; DNA_pol_phi; DNA polymerase phi]	*Escherichia* phage RB69 (3e-57)	98.8	0	N
163	112771-112986	+	71	Hypothetical protein	*Escherichia* phage phiE142 (5e-45)	100	0	N
164	113048-113506	+	152	Site-specific RNA endonuclease [PF10715; REGB_T4; T4-page Endoribonuclease RegB]	*Escherichia* phage vB_EcoM_JS09 (8e-108)	99.3	0	N
165	113515-114057	+	180	Endoribonuclease []PF01464; SLT; Transglycosylase SLT domain}	*Escherichia* phage mobillu (5e-128)	99.4	0	Y
166	114054-114401	+	115	Valyl-tRNA synthetase modifier	*Escherichia* phage APCEc01 (1e-78)	100	0	Y
167	114394-114861	+	155	Macro domain protein [PF01661; Macro; Macro domain]	*Escherichia* coli O157 typing phage 3 (2e-110)	100	0	N
168	114858-115070	+	70	Hypothetical protein	*Escherichia* phage vB_EcoM_IME537 (3e-45)	100	0	N
169	115067-115273	+	68	Hypothetical protein	*Escherichia* phage F2 (3e-43)	100	0	N
170	115270-115452	+	60	Hypothetical protein [PF05823; Gp-FAR-1; Nematode fatty acid retinoid binding protein]	*Escherichia* phage RB69 (5e-33)	98.3	0	N
171	115462-116043	+	193	Tk thymidine kinase [PF00265; TK; Thymidine kinase]	*Escherichia* phage RB69 (2e-141)	100	0	N
172	116071-116283	+	70	Hypothetical protein [PF08700; Vps51; Vps51/Vps67]	*Escherichia* phage vB_EcoM_JS09 (8e-42)	100	0	N
173	116296-116598	+	100	Lysis inhibition regulator [PF03126; Plus-3; Plus-3 domain]	*Escherichia* phage moskry (3e-68)	99.0	1	Y
174	116700-116879	+	59	Hypothetical protein	*Escherichia* phage phiE142 (3e-35)	100	0	N
175	116887-117093	+	68	Hypothetical protein	*Escherichia* phage SF (2e-42)	100	0	N
176	117139-117261	+	40	Hypothetical protein	*Escherichia* phage HX01 (3e-18)	100	1	N
177	117374-117904	+	176	Hypothetical protein [PF09765; FANCL_d1; FANCL UBC-like domain 1]	*Escherichia* phage APCEc01 (7e-125)	98.8	0	N

178	117914-118387	+	157	Signal-peptide domain-containing protein [PF11087; PRD1_DD; PRD1 phage membrane DNA delivery]	*Escherichia* phage moha (6e-109)	98.7	1	N
179	118387-119373	+	328	Nucleotidyltransferase [PF10127; RlaP; RNA repair pathway DNA polymerase beta family]	*Escherichia* phage OLB35 (0.0)	99.4	0	N
180	119405-119686	+	93	Hypothetical protein [PF05110; AF-4; AF-4 proto-oncoprotein N-terminal region]	*Escherichia* phage moskry (3e-58)	96.8	0	Y
181	119805-120785	+	326	Thioredoxin [PF13191; AAA_16; AAA ATPase domain]	*Escherichia* phage mogra (0.0)	99	0	N
182	120924-121211	+	95	Thioredoxin [PF09553; RE_Eco47II; Eco47II restriction endonuclease]	*Escherichia* phage mobillu (4e-61)	99	0	N
183	121270-121797	+	175	Thioredoxin [PF09228; Prok-TraM; Prokaryotic Transcriptional repressor TraM]	*Escherichia* phage FP43 (1e-121)	98.8	0	N
184	121860-122855	+	331	Thioredoxin [PF09074; Mer2; Mer2]	*Escherichia* phage mobillu (0.0)	98.4	0	N
185	122911-123846	+	311	Thioredoxin	*Escherichia* phage mogra (0.0)	98.3	0	N
186	123909-124859	+	316	Thioredoxin [PF15022; DUF4522; Protein of unknown function]	*Escherichia* phage mogra (0.0)	98.7	0	N
187	124859-125164	+	101	Hypothetical protein [PF17022; PTP2; Polar tube protein 2 from Microsporidia]	*Escherichia* phage vB_EcoM_NBG1 (4e-68)	98	0	N
188	125164-125577	+	157	Thioredoxin	*Escherichia* phage vB_EcoM-ZQ3 (1e-93)	98.5	2	N
189	125570-125833	+	87	Thioredoxin [PF00462; Glutaredoxin; Glutaredoxin]	*Escherichia* phage vB_EcoM_PhAPEC2 (8e-58)	100	0	N
190	125830-126045	+	71	Hypothetical protein [PF04452; Methyltrans_RNA; RNA methyltransferase]	*Escherichia* phage BF15 (3e-43)	98.5	0	N
191	126048-126218	+	56	Hypothetical protein	*Escherichia* phage moskry (9e-31)	98.2	0	N
192	126347-126517	+	56	Hypothetical protein	*Escherichia* phage vB_EcoM_JS09 (4e-33)	100	0	N
193	126519-126944	+	141	Peptidase inhibitor [PF10465; Inhibitor_I24; PinA peptidase inhibitor]	*Escherichia* phage vB_EcoM_KAW3E185 (6e-97)	99.2	0	N

194	126980-127453	+	157	Recombination endonuclease [PF09124; Endonuc-dimeris; T4 recombination endonuclease VII, dimerisation]	*Escherichia* phage ST0 (1e-112)	99.3	0	N
195	127450-129267	+	605	Anaerobic ribonucleoside-triphosphate reductase [PF13597; NRDD; Anaerobic ribonucleoside-triphosphate reductase]	*Escherichia* phage S143_2 (0.0)	99.6	0	N
196	129264-129734	+	156	Ribonucleotide reductase of class III (anaerobic), activating protein [PF13353; Fer4_12; 4Fe-4S single cluster domain]	*Escherichia* phage vB_EcoM_Lutter (8e-111)	98	0	N
197	129845-130060	+	71	Hypothetical protein [PF12046; CCB1; Cofactor assembly of complex C subunit B]	*Escherichia* phage SF (2e-41)	100	1	N
198	130063-130380	+	105	Hypothetical protein [PF00626; Gelsolin; Gelsolin repeat]	*Escherichia* phage vB_EcoM_PhAPEC2 (5e-71)	100	0	N
199	130346-130669	+	107	Glutaredoxin [PF00462; Glutaredoxin; Glutaredoxin]	*Escherichia* phage vB_EcoM_PhAPEC2 (2e-70)	99	0	N
200	130836-131084	+	82	Hypothetical protein	*Escherichia* phage vB_EcoM_WFL6982 (5e-51)	100	0	N
201	131092-131385	+	97	Hypothetical protein [PF17603; DUF5499; Family of unknown function]	*Escherichia* phage vB_EcoM_JS09 (5e-63)	100	0	N
202	131393-131527	+	44	Hypothetical protein [PF17583; DUF5484; Family of unknown function]	*Escherichia* phage HX01 (3e-23)	100	0	N
203	131524-131724	+	66	Hypothetical protein [PF17593; DUF5490; Family of unknown function]	*Escherichia* phage vB_EcoM_PhAPEC2 (1e-41)	100	0	N
204	131788-132027	+	79	Hypothetical protein	*Escherichia* phage ST0 (8e-50)	100	0	N
205	132094-132426	+	110	Hypothetical protein	*Escherichia* coli O157 typing phage 3 (3e-72)	99	0	N
206	132423-132650	+	75	Hypothetical protein [DUF5495; DUF5495; Family of unknown function]	*Escherichia* phage RB69 (1e-45)	97.3	0	N
207	132647-132916	+	89	Hypothetical protein [PF14630; ORC5_C; Origin recognition complex (ORC) subunit 5 C-terminus]	*Escherichia* phage HX01 (1e-58)	100	0	N
208	132989-133546	+	185	RNA polymerase sigma factor [PF17595; DUF5491; Family of unknown function]	*Escherichia* phage vB_EcoM_JS09 (9e-135)	100	0	N
209	133536-133745	+	69	Hypothetical protein [PF17595; DUF5491; Family of unknown function]	*Escherichia* phage APCEc01 (3e-42)	100	0	N

210	133747-134070	+	107	Hypothetical protein [PF10849; DUF2654; Protein of unknown function]	*Escherichia* phage RB69 (3e-69)	100	0	N
211	134291-134464	+	57	Hypothetical protein [PF17588; DUF5486; Family of unknown function]	*Escherichia* phage APCEc01 (1e-33)	100	0	N
212	134534-135553	+	339	Endonuclease subunit [PF00149; Metallophos; Calcineurin-like phosphoesterase]	*Escherichia* phage RB69 (0.0)	99.4	0	N
213	135550-135807	+	85	Hypothetical protein	*Escherichia* phage vB_EcoM_JS09 (7e-55)	100	0	N
214	135794-136033	+	79	Hypothetical protein [PF17589; DUF5487; Family of unknown function]	*Escherichia* phage vB_EcoM-ZQ3 (3e-50)	98.7	0	N
215	136030-137718	+	562	Endonuclease subunit [PF13476; AAA_23; AAA domain]	*Escherichia* phage RB69 (0.0)	100	0	N
216	137773-137961	+	62	Hypothetical protein [PF17470; Gp45_2; Phage gene product 45.2]	*Escherichia* phage RB69 (4e-38)	100	0	N
217	137974-138390	+	138	RNA polymerase binding protein [PF10789; Phage_RpbA; Phage RNA polymerase binding, RpbA]	*Escherichia* phage vB_EcoM_PhAPEC2 (2e-98)	100	0	N
218	138433-139119	+	228	Sliding clamp [PF09116; gp45-slide_C; gp45 sliding clamp, C terminal]	*Escherichia* phage RB69 (6e-165)	100	0	N
219	139195-140157	+	320	Clamp loader small subunit [PF00004; AAA; ATPase family associated with various cellular activities]	*Escherichia* phage APCEc01 (0.0)	100	0	N
220	140159-140722	+	187	Clamp loader small subunit [PF16790; Phage_clamp_A; Bacteriophage clamp loader A subunit]	*Escherichia* phage RB69 (3e-133)	99.4	0	N
221	140725-141093	+	122	Translation repressor protein [PF01818; Translat_reg; Bacteriophage translational regulator]	*Escherichia* phage RB69 (3e-84)	100	0	N
222	141175-143886	+	903	DNA polymerase [PF00136; DNA_pol_B; DNA polymerase family B]	*Escherichia* phage RB69 (0.0)	99.8	0	N
223	143927-144562	+	211	Sarabinose 5-phosphate isomerase [PF01380; SIS; SIS domain]	*Escherichia* phage moha (3e-152)	98.5	0	N
224	144559-144702	+	47	Hypothetical protein	*Escherichia* phage F2 (2e-23)	97.8	1	N
225	144744-146429	+	561	Bifunctional protein GlmU [PF00483; NTP_transferase; Nucleotidyl transferase]	*Escherichia* phage moha (0.0)	99.8	0	N
226	146429-146794	+	121	Hypothetical protein	*Escherichia* phage RB69 (3e-81)	93.3	0	N

227	146850-148010	+	386	Peptidase [PF01136; Peptidase_U32; Peptidase family U32]	*Escherichia* phage mogra (0.0)	99.4	0	N
228	148007-148246	+	79	Hypothetical protein	*Escherichia* phage phiE142 (8e-49)	98.7	0	N
229	148289-149005	+	238	Deoxycytidylate 5-hydroxymethyltransferase [PF00303; Thymidylat_synt; Thymidylate synthase]	*Escherichia* phage p000v (3e-179)	99.5	0	N
230	149005-149904	+	299	Hypothetical protein	*Escherichia* phage APCEc01 (0.0)	100	0	N
231	149906-150454	+	182	Hypothetical protein [PF13173; AAA_14; AAA domain]	*Escherichia* phage vB_EcoM_KAW3E185 (5e-130)	97.8	0	N
232	150553-151725	+	390	Recombination and repair protein [PF00154; RecA; recA bacterial DNA recombination protein]	*Escherichia* phage p000v (0.0)	99.4	0	N
233	151718-152059	+	113	Head vertex assembly initiator [PF11113; Phage_head_chap; Head assembly gene product]	*Escherichia* phage vB_EcoM-ZQ3 (2e-75)	98.2	0	N
234	152069-153511	+	480	DNA helicase [PF03796; DnaB_C; DnaB-like helicase C terminal domain]	*Escherichia* phage APCEc01 (0.0)	99.7	0	N
235	153600-153974	+	124	Hypothetical protein [PF06825; HSBP1; Heat shock factor binding protein 1]	*Escherichia* phage RB69 (4e-86)	100	0	N
236	154030-154347	+	105	Hypothetical protein	*Escherichia* phage vB_EcoM_PhAPEC2 (4e-73)	100	0	N
237	154344-154532	+	62	Discriminator of mRNA degradation [PF17587; Dmd; Discriminator of mRNA degradation]	*Escherichia* phage vB_EcoM_PhAPEC2 (5e-35)	98.3	0	N
238	154604-154972	+	122	Immunity protein [PF17578; DUF5481; Family of unknown function]	*Escherichia* phage moha (1e-84)	98.3	0	Y
239	155034-155282	+	82	Imm immunity to superinfection membrane protein [PF14373; Imm_superinfect; Superinfection immunity protein]	*Escherichia* phage RB69 (1e-48)	98.7	2	N
240	155346-155639	+	97	Sp spackle periplasmic protein [PF17979; zf-CRD; Cysteine rich domain with multizinc binding regions]	*Escherichia* phage RB69 (1e-65)	98.9	0	Y
241	155641-156291	+	216	Hypothetical protein	*Escherichia* phage vB_EcoM_WFbE185 (1e-158)	100	0	N
242	156293-156490	+	65	Hypothetical protein	*Escherichia* phage RB69 (1e-39)	100	0	N

243	156510-156977	+	155	Hypothetical protein [PF07068; Gp23; Major capsid protein Gp23]	*Escherichia* phage vB_EcoM_JS09 (5e-110)	100	0	N
244	157017-158039	+	340	DNA primase [PF08275; Toprim_N; DNA primase catalytic core, N-terminal domain]	*Escherichia* phage phiE142 (0.0)	100	0	N
245	158036-158233	-	65	Hypothetical protein	*Escherichia* phage vB_EcoM_G2469 (5e-35)	100	1	N
246	158324-158845	+	173	DCTP pyrophosphatase [PF08761; dUTPase_2; dUTPase]	*Escherichia* phage vB_EcoM_JS09 (5e-125)	99.4	0	N
247	158891-159127	+	78	Small outer capsid protein [PF16855; Soc; Small outer capsid protein]	*Escherichia* virus vB_Eco_mar005P1 (1e-49)	98.7	0	N
248	159425-159673	+	82	Hypothetical protein	*Escherichia* phage APCEc01 (2e-52)	100	0	N
249	159670-159849	+	59	Hypothetical protein	*Escherichia* phage vB_EcoM_JS09 (1e-33)	100	0	N
250	159849-160313	+	154	Transcription modulator under heat shock	*Escherichia* phage vB_EcoM_PhAPEC2 (5e-109)	100	0	N
251	160315-160494	+	59	Hypothetical protein	*Escherichia* phage vB_EcoM_G2285 (1e-33)	98.3	0	N
252	160491-160655	+	54	Hypothetical protein	*Escherichia* phage RB69 (4e-28)	100	0	N
253	160711-161292	+	193	ModB ADP-ribosylase	*Escherichia* phage vB_EcoM_G53 (4e-142)	100	0	N
254	161350-161958	+	202	ADP-ribosylase	*Escherichia* phage mobillu (5e-148)	98.5	0	N
255	162112-162858	+	248	Srd anti-sigma factor [PF01621; Fusion_gly_K; Cell fusion glycoprotein K]	*Escherichia* phage vB_EcoM_NBG1 (4e-178)	99.1	0	N
256	162861-163172	+	103	Hypothetical protein [PF04666; Glyco_transf_54; N-Acetylglucosaminyltransferase-IV (GnT-IV) conserved region]	*Escherichia* phage vB_EcoM_JS09 (6e-69)	100	0	N
257	163169-164482	+	437	DNA helicase [PF18343; SH3_14; Dda helicase SH3 domain]	*Escherichia* phage APCEc01 (0.0)	99.7	0	N
258	164492-165169	+	225	Exodeoxyribonuclease [PF16473; DUF5051; 3' exoribonuclease, RNase T-like]	*Escherichia* phage p000v (1e-165)	99.5	0	N

259	165236-165730	+	164	Transcriptional regulator [PF17613; motB; Modifier of transcription]	*Escherichia* phage moskry (3e-115)	97.5	0	N
260	165791-166255	+	154	Modifier of transcription [PF17613; motB; Modifier of transcription]	*Escherichia* phage moha (1e-106)	98.7	0	N
261	166265-166684	+	139	Transcriptional regulator [PF17613; motB; Modifier of transcription]	*Escherichia* phage moha (5e-91)	94.2	0	N
262	166744-167268	+	174	Transcriptional regulator	*Escherichia* phage moha (8e-126)	98.8	0	N
263	167326-167553	+	75	Protein Cef	*Escherichia* phage S143_2 (2e-46)	98.6	0	N
264	167553-167966	+	137	mRNA metabolism modulator	*Escherichia* phage vB_EcoM_PhAPEC2 (5e-95)	97	0	N
265	167966-168076		37	Zinc ribbon domain protein [PF09723; Zn-ribbon_8; Zinc ribbon domain]	*Escherichia* phage mobillu (3e-17)	97	0	N
