# Supplementary material for: Characterization of Five Lytic Bacteriophages as New Members of the Genus Mosigvirus, Infecting Multidrug-Resistant Shiga Toxin-Producing Escherichia coli and Their Antibiofilm Activity
Source: Viruses. 2025 Nov 13;17(11):1501. doi: 10.3390/v17111501 (PMC12656860; doi:10.3390/v17111501)
Supplement: Supplementary file 1 [file viruses-17-01501-s001.zip › Table S2.pdf]

Table S2. Features of predicted ORFs and their homology to STEC phage ΦC.

Gene product				Putative function [Conserved domain]	Best match organism (E-value)	Identity (%)	Predicted TMHHM and signal peptide	
ORF No.	Range	Strand	Length (AA)				TMHHM	SignalP
1	3-1568	-	522	Baseplate hub subunit and tail length	*Escherichia* phage F2 (0.0)	99.4	0	N
2	1565-2035	-	156	Baseplate distal hub subunit	*Shigella* phage Shf125875 (1e-110)	100	0	N
3	2046-3218	-	390	Baseplate hub subunit [PF09097; Phage-tail_1; Baseplate structural protein, domain 1]	*Shigella* phage Shf125875 (1e-110)	100	0	N
4	3215-3967	-	250	Baseplate hub assembly protein	*Shigella* phage JK45 (0.0)	99.6	0	N
5	4015-4641	+	208	Baseplate hub subunit	*Shigella* phage SSE1 (6e-153)	99.5	0	N
6	4641-5039	+	132	Putative baseplate wedge subunit	*Escherichia* phage vB_EcoM_G2469 (6e-90)	99.2	0	N
7	5039-5533	+	164	UvsY-like recombination mediator	*Shigella* phage phi25-307 (3e-116)	100	0	N
8	5533-5757	+	74	Hypothetical protein	*Escherichia* phage ST0 (4e-46)	100	0	N
9	5790-5957	+	55	Hypothetical protein [PF10886; DUF2685; Protein of unknown function (DUF2685)]	*Escherichia* phage vB_EcoM_JS09 (9e-32)	100	0	N
10	6014-6247	-	77	DNA helicase [PF11637; UvsW-1; UvsW.1 domain]	*Escherichia* phage F2 (6e-45)	98.7	0	N
11	6273-7787	-	504	ATP-dependent DNA helicase [PF04851; ResIII; Type III restriction enzyme, res subunit]	*Escherichia* phage PTK (0.0)	99.6	0	N
12	7838-8506	+	222	Protein Inh	*Escherichia* phage S143_2 (2e-157)	100	0	N
13	8516-9358	+	280	Putative 6.6 kDa protein [PF13927; Ig_3; Immunoglobulin domain]	*Escherichia* phage S143_2 (9e-37)	100	0	N

14	9458-9652	+	64	Putative 6.6 kDa protein	*Escherichia* phage S143_2 (9e-37)	100	0	N
15	9649-9900	+	85	Hypothetical protein	*Shigella* phage phi25-307 (3e-54)	98.8	0	N
16	10020-11027	+	335	Putative RNA ligase II [PF09414; RNA_ligase; RNA ligase]	*Escherichia* phage 55 (0.0)	97.9	0	N
17	11056-12339	-	427	Capsid vertex protein [PF07068; Gp23; Major capsid protein Gp23]	*Escherichia* phage vB_EcoM_PhAPEC2 (0.0)	99.7	0	N
18	12441-12710	+	89	Hypothetical protein	*Escherichia* phage vB_EcoM_MM02 (2e-57)	98.8	0	N
19	12763-14331	-	522	Major capsid protein [PF07068; Gp23; Major capsid protein Gp23]	scherichia phage vB_EcoM_SQ17 (0.0)	99.6	0	N
20	14349-15161	-	270	Prohead scaffolding protein	*Escherichia* phage AlbertHofmann (0.0)	99.6	0	N
21	15195-15836	-	213	Head maturation protease	*Escherichia* phage F2 (1e-152)	100	0	N
22	15836-16261	-	141	Head scaffolding protein	*Escherichia* phage phiC120 (3e-95)	99.2	0	N
23	16261-16491	-	76	Prohead	*Escherichia* coli O157 typing phage 3 (5e-42)	100	0	N
24	16491-18062	-	523	Portal protein	*Escherichia* phage APCEc01 (0.0)	100	0	N
25	18147-18638	-	163	Tail protein [PF06841; Phage_T4_gp19; T4-like virus tail tube protein gp19]	*Escherichia* phage RB69(5e-116)	100	0	N
26	18752-20734	-	660	Tail sheath	*Escherichia* phage F2 (0.0)	99.8	0	N
27	20765-22600	-	611	Terminase large subunit [PF03237; Terminase_6N; Terminase large subunit, T4likevirus-type, N-terminal]	*Shigella* phage SHSML-52-1 (0.0)	99.8	0	N
28	22584-23078	-	164	Terminase small subunit	*Escherichia* phage vB_EcoM_JS09(2e-116)	99.3	0	N
29	23088-23909	-	273	Putative tail sheath stabilizer and completion	*Escherichia* phage vB_EcoM_G2285 (0.0)	100	0	N

30	23962-24726	-	254	Head closure Hc2	*Escherichia* phage F2 (0.0)	99.2	0	N
31	24713-25348	-	211	Putative endonuclease	*Escherichia* phage F2 (1e-154)	100	0	N
32	25350-26276	-	308	Head-tail adaptor Ad2	*Escherichia* phage vB_EcoM_JS09 (0.0)	100	0	N
33	26309-27757	-	482	Fibritin [PF07921; Fibritin_C; Fibritin C-terminal region]	*Escherichia* phage 308Ecol101PP(0.0)	99.3	0	N
34	27757-29340	-	527	Short tail fiber protein [PF14928; S_tail_recep_bd; Short tail fibre protein receptor-binding domain]	*Escherichia* phage S143_2 (0.0)	99.2	0	N
35	29337-29996	-	219	Baseplate wedge subunit	*Shigella* phage phi25-307 (7e-158)	98.6	0	N
36	29996-31801	-	601	Baseplate wedge subunit [PF07880; T4_gp9_10; Bacteriophage T4 gp9/10-like protein]	*Shigella* phage phi25-307]0.0)	99.6	0	N
37	31801-32673	-	290	Putative baseplate wedge tail fiber connector [PF07880; T4_gp9_10; Bacteriophage T4 gp9/10-like protein]	*Escherichia* phage vB_EcoM_G2285 (0.0)	100	0	N
38	32746-33750	-	334	Baseplate wedge subunit	*Escherichia* phage vB_EcoM-RPN187(0.0)	100	0	N
39	33743-36841	-	1032	Baseplate wedge subunit [PF21428Gp7_helical ; Baseplate wedge protein gp7, helical domain]	*Escherichia* phage 308Ecol101PP(0.0)	99.7	1	N
40	36838-38811	-	657	Baseplate wedge subunit	*Escherichia* phage vB_EcoM-ZQ3 (0.0)	100	0	N
41	38820-39113	-	97	PAAR motif of membrane proteins	*Escherichia* phage RB69 (1e-63)	98.9	0	N
42	39116-39589	-	157	Hypothetical protein	*Escherichia* phage 308Ecol101PP(6e-111)	99.3	0	N
43	39635-41368	-	577	Baseplate hub subunit and tail lysozyme [PF00959 ; Phage_lysozyme ; Phage lysozyme]	*Escherichia* phage vB_EcoM-RPN187(0.0)	99.8	0	N
44	41368-41943	-	191	Baseplate wedge subunit [PF11246; Phage_gp53; Base plate wedge protein 53]	*Shigella* phage phi25-307 (1e-137)	100	0	N

45	42005-42454	+	149	Head completion protein [PF08722; Tn7_Tnp_TnsA_N; TnsA endonuclease N terminal]	*Escherichia* phage ST2 (7e-106)	100	0	N
46	42457-43278	+	273	DNA end protector [PF13422; DUF4110; Domain of unknown function (DUF4110)]	Enterobacteria phage ATK47 (0.0)	100	0	N
47	43381-43965	+	194	Tail completion and sheath stabilizer protein [PF06841; Phage_T4_gp19; T4-like virus tail tube protein gp19]	*Escherichia* phage vB_EcoM_JS09 (3e-142)	99.4	0	N
48	44019-44753	+	244	Deoxynucleoside monophosphate kinase	*Escherichia* phage APCEc01 (6e-178)	100	0	N
49	44758-44988	+	76	Chaperone for tail fiber formation	*Escherichia* phage APCEc01 (4e-42)	100	0	N
50	44988-45443	+	151	RNA ligase	*Escherichia* phage vB_EcoM_JS09 (2e-107)	99.3	0	N
51	45521-45808	+	95	Internal virion protein	*Escherichia* phage WG01 (4e-60)	100	0	N
52	45880-46065	+	61	Hypothetical protein	*Escherichia* phage WG01 (5e-33)	100	2	N
53	46067-46441	+	124	Hypothetical protein	*Escherichia* phage F2 (3e-87)	100	0	N
54	46444-46734	+	96	Hypothetical protein	*Shigella* phage phi25-307 (1e-62)	100	0	N
55	46739-47254	+	171	Hypothetical protein	*Escherichia* phage vB_EcoM_SQ17 (5e-119)	98.8	0	N
56	47507-47851	+	114	Hypothetical protein	*Escherichia* phage vB_EcoM_TU01 (2e-78)	99.1	0	N
57	48232-48696	+	154	Hypothetical protein	*Escherichia* phage vB_EcoM-RPN187 (2e-105)	99.3	0	N
58	48820-49125	+	101	Hypothetical protein	*Escherichia* phage ECO07P3 (2e-66)	99	0	N
59	49193-49420	+	75	Hypothetical protein [PF05798; Phage_FRD3; Bacteriophage FRD3 protein]	*Escherichia* phage SF (5e-48)	100	0	N
60	49491-50084	+	197	Hypothetical protein [PF08644; SPT16; FACT complex subunit (SPT16/CDC68)]	*Escherichia* phage RB69 (7e-136)	100	0	N

61	50134-50730	+	198	Hypothetical protein	*Escherichia* coli O157 typing phage 3 (4e-144)	98.9	0	N
62	51073-51432	+	119	Hypothetical protein	*Escherichia* phage vB_EcoM_JS09 (1e-82)	100	2	N
63	51429-51734	+	101	Hypothetical protein	*Escherichia* phage vB_EcoM_JS09 (1e-66)	100	0	N
64	51744-52016	+	90	Hypothetical protein	*Escherichia* phage SF (8e-60)	100	0	N
65	52026-52223	+	65	Hypothetical protein	*Escherichia* phage HX01 (1e-36)	96.9	0	N
66	52286-52525	+	79	Hypothetical protein	*Escherichia* phage HX01(3e-51)	98.7	0	N
67	52554-53510	+	318	Hypothetical protein	*Escherichia* phage PNJ-6 (0.0)	98.7	0	N
68	53592-53792	+	66	Hypothetical protein	*Escherichia* phage 55 (4e-41)	100	0	N
69	53849-54154	+	101	Hypothetical protein	*Escherichia* phage PTK (1e-67)	99	2	N
70	54156-54842	+	228	Hypothetical protein	*Escherichia* phage PNJ-6 (3e-170)	99.5	0	N
71	54925-55323	+	132	Hypothetical protein	*Escherichia* phage vB_EcoM_SQ17 (4e-86)	96.9	2	N
72	55320-55556	+	78	Hypothetical protein	*Escherichia* phage vB_EcoM_PhAPEC2 (5e-46)	98.7	0	N
73	55549-56004	+	151	Nudix hydrolase [PF00293; NUDIX; NUDIX domain]	*Shigella* phage JK45 (1e-109)	100	0	N
74	56039-56527	+	162	Endolysin protein e [PF00959; Phage_lysozyme; Phage lysozyme]	*Escherichia* phage ChristianSchoenbein (4e-116)	100	0	N
75	56605-56805	+	66	Internal head protein	*Escherichia* phage RB69 (2e-37)	98.4	0	N
76	56864-57277	+	137	Endonuclease V N-glycosylase UV repair enzyme	*Escherichia* phage HX01 (4e-96)	99.2	0	N
77	57369-57671	+	100	Internal protein II	*Escherichia* phage ECO07P1 (1e-64)	98	0	N

78	57785-58114	+	109	Hypothetical protein	*Shigella* phage phi25-307 (1e-71)	100	0	N
79	58287-58826	+	179	Hypothetical protein	*Escherichia* phage PHB12 (2e-130)	99.4	0	Y
80	58823-59131	+	102	Hypothetical protein	*Escherichia* phage vB_EcoM_PhAPEC2 (9e-70)	98	0	N
81	59138-59500	+	120	Autonomous glycyl radical cofactor GrcA [PF01228; Gly_radical; Glycine radical]	*Escherichia* phage F2 (4e-82)	100	0	N
82	59500-59724	+	74	Hypothetical protein	*Escherichia* phage F2 (3e-46)	98.6	0	N
83	59714-59980	+	88	Hypothetical protein	*Escherichia* phage RB69 (4e-57)	98.8	0	N
84	59980-60195	+	71	Hypothetical protein	*Escherichia* phage vB_EcoM_JS09 (1e-44)	100	0	N
85	60258-60716	+	152	Endoribonuclease	*Escherichia* phage vB_EcoM_WFbE185 (7e-106)	98	0	N
86	60725-61267	+	180	Putative 20.7 kDa protein	*Escherichia* phage S143_2 (4e-128)	100	0	Y
87	61264-61611	+	115	Vs valyl-tRNA synthetase modifier	*Escherichia* phage RB69 (2e-77)	99.1	0	Y
88	61604-62071	+	155	Phosphatase [PF01661; Macro; Macro domain]	*Escherichia* phage F2 (3e-110)	100	0	N
89	62068-62280	+	70	Hypothetical protein	*Escherichia* phage ST0 (2e-44)	100	0	N
90	62277-62462	+	61	Hypothetical protein	*Escherichia* phage vB_EcoM_SQ17 (7e-35)	100	0	N
91	62459-62647	+	62	Hypothetical protein	*Shigella* phage SSE1 (3e-34)	100	0	N
92	62649-63230	+	193	Thymidine kinase [PF00265; TK; Thymidine kinase]	*Escherichia* coli O157 typing phage 3 (6e-140)	98.9	0	N
93	63258-63470	+	70	Hypothetical protein	*Escherichia* phage HX01 (3e-41)	98.5	0	N

94	63483-63785	+	100	Lysis inhibition regulator	*Escherichia* phage moskry (1e-67)	99	1	Y
95	63888-64067	+	59	Hypothetical protein	*Escherichia* phage F2 (6e-35)	98.3	0	N
96	64075-64239	+	54	Hypothetical protein	*Escherichia* phage vB_EcoM-ZQ3 (1e-32)	98.1	0	N
97	64236-64331	+	31	Hypothetical protein	*Escherichia* phage vB_EcoM_JS09 (1e-12)	100	1	N
98	64331-64546	+	71	Hypothetical protein	*Escherichia* phage PNJ-6 (9e-44)	100	0	N
99	64592-64714	+	40	Hypothetical protein	*Escherichia* phage ST0 (4e-18)	100	1	N
100	64711-64890	+	59	Hypothetical protein	*Escherichia* phage vB_EcoM-ZQ3 (3e-33)	98.3	0	N
101	64892-65422	+	176	Hypothetical protein	*Escherichia* phage phiE142(3e-121)	96	0	N
102	65432-65905	+	157	Signal-peptide domain-containing protein	*Escherichia* phage vB_EcoM_WFL6982 (2e-107)	96.8	2	N
103	65905-66915	+	336	Nucleotidyltransferase	*Escherichia* phage HX01 (0.0)	97.3	0	N
104	66947-67228	+	93	Hypothetical protein	*Escherichia* phage vB_EcoM-RPN187(1e-59)	100	1	Y
105	67348-68328	+	326	Hypothetical protein [PF13191; AAA_16; AAA ATPase domain]	*Escherichia* phage vB_EcoM_JS09 (0.0)	996	0	N
106	68467-68754	+	95	Thioredoxin	*Escherichia* phage HX01 (4e-61)	98.5	0	N
107	68813-69340	+	175	Hypothetical protein	*Escherichia* phage vB_EcoM_JS09 (8e-123)	100	0	N
108	69403-70380	+	325	Hypothetical protein	*Escherichia* phage vB_EcoM_JS09 (0.0)	98.1	0	N
109	70436-71368	+	310	Hypothetical protein	*Escherichia* phage vB_EcoM-RPN187 (0.0)	99	0	N

110	71431-72381	+	316	Putative 36.3 kDa protein	*Escherichia* phage S143_2 (0.0)	98.4	0	N
111	72381-72686	+	101	Hypothetical protein	*Escherichia* phage vB_EcoM_JS09 (4e-69)	100	0	N
112	72686-73099	+	137	Thioredoxin	Enterobacteria phage ATK47 (3e-94)	99.2	2	N
113	73092-73355	+	87	Phage-associated thioredoxin	*Escherichia* phage F2 (2e-57)	100	0	N
114	73352-73708	+	118	Hypothetical protein	*Shigella* phage Shf125875 (2e-79)	100	0	N
115	73892-74008	+	38	Hypothetical protein	*Escherichia* phage RB69 (9e-19)	100	0	N
116	74010-74435	+	141	Protease inhibitor	*Escherichia* phage vB_EcoM-ZQ3 (4e-97)	97.8	0	N
117	74475-74948	+	157	Endonuclease VII	*Escherichia* phage RB69 (5e-113)	100	0	N
118	74945-76762	+	605	Ribonucleotide reductase of class II [PF13597; NRDD; Anaerobic ribonucleoside-triphosphate reductase]	*Escherichia* phage PHB12 (0.0)	99.3	0	N
119	76759-77229	+	156	Anaerobic ribonucleotide reductase small subunit [PF04055; Radical_SAM ; Radical SAM superfamily]	*Escherichia* phage phiE142 (9e-112)	99.3	0	N
120	77340-77555	+	71	Hypothetical protein	*Escherichia* phage vB_EcoM_JS09 (4e-41)	100	1	N
121	77558-77875	+	105	Hypothetical protein	*Escherichia* coli O157 typing phage 3 (7e-69)	97.1	0	N
122	77841-78164	+	107	Glutaredoxin	*Escherichia* phage ST0 (4e-71)	100	0	N
123	78330-78512	+	60	Hypothetical protein	*Escherichia* phage vB_EcoM_JS09 (5e-35)	98.3	0	N
124	78509-78757	+	82	Hypothetical protein	*Shigella* phage SSE1 (2e-49)	98.7	0	N
125	78765-79058	+	97	Hypothetical protein	*Escherichia* phage vB_EcoM_JS09 (8e-63)	100	0	N
126	79066-79200	+	44	Hypothetical protein	*Escherichia* phage ST0 (4e-23)	100	0	N

127	79197-79397	+	66	Hypothetical protein	*Escherichia* phage 348Ecol098PP (9e-42)	100	0	N
128	79461-79700	+	79	Hypothetical protein	*Shigella* phage phi25-307 (2e-49)	98.7	0	N
129	79728-80099	+	123	Hypothetical protein	*Shigella* phage phi25-307 (4e-81)	98.3	0	N
130	80096-80323	+	75	gp55.1 conserved hypothetical protein	*Escherichia* phage RB69 (2e-45)	98.6	0	N
131	80320-80589	+	89	Hypothetical protein	*Escherichia* phage moskry (3e-58)	98.8	0	N
132	80662-81219	+	185	RNA polymerase sigma factor	*Escherichia* phage RB69 (7e-134)	99.4	0	N
133	81209-81418	+	69	Hypothetical protein	*Escherichia* phage APCEc01 (5e-42)	100	0	N
134	81420-81743	+	107	Hypothetical protein	*Escherichia* phage p000v (1e-68)	99	0	N
135	81964-82137	+	57	Hypothetical protein	*Escherichia* phage APCEc01 (2e-33)	100	0	N
136	82207-83226	+	339	Recombination-related endonuclease	*Escherichia* phage FL31 (0.0)	98.8	0	N
137	83223-83480	+	85	Hypothetical protein	*Escherichia* phage vB_EcoM_JS09 (1e-54)	100	0	N
138	83467-83706	+	79	Hypothetical protein	*Escherichia* phage vB_EcoM-ZQ3 (4e-50)	98.7	0	N
139	83703-85391	+	562	SbcC-like subunit of palindrome specific endonuclease [PF13476; AAA_23; AAA domain]	*Escherichia* phage RB69 (0.0)	100	0	N
140	85446-85634	+	62	Protein GP45.2	*Escherichia* phage RB69 (6e-38)	100	0	N
141	85647-86063	+	138	RNA polymerase binding	*Escherichia* phage vB_EcoM_PhAPEC2 (3e-98)	100	0	N
142	86106-86792	+	228	Putative sliding clamp [IPR046389 ; Sliding_clamp_T4 ; Sliding clamp]	*Escherichia* phage vB_EcoM_WFL6982 (4e-164)	99.5	0	N

143	86868-87830	+	320	Clamp loader of DNA polymerase [IPR046388 ; T4_Clamp_Loader_L ; Sliding-clamp-loader large subunit]	*Escherichia* phage APCEc01 (0.0)	100	0	N
144	87832-88395	+	187	Clamp loader	*Escherichia* phage RB69 (4e-133)	99.4	0	N
145	88398-88766	+	122	Translation repressor [PF01818; Translat_reg; Bacteriophage translational regulator]	*Escherichia* phage RB69 (5e-84)	100	0	N
146	88848-91559	+	903	DNA polymerase	*Escherichia* phage vB_EcoM_JS09 (0.0)	99.7	0	N
147	91600-92235	+	211	Arabinose 5-phosphate isomerase [PF01380; SIS; SIS domain]	*Escherichia* phage RB69 (2e-153)	100	0	N
148	92232-92375	+	47	Hypothetical protein	*Shigella* phage Shf125875 (1e-23)	100	0	N
149	92417-94102	+	561	Hypothetical protein [PF00483; NTP_transferase; Nucleotidyl transferase]	*Escherichia* phage vB_EcoM_JS09 (0.0)	100	0	N
150	94102-94467	+	121	Phosphoheptose isomerase	*Escherichia* phage RB69 (6e-81)	93.3	0	N
151	94523-95683	+	386	Peptidase	Enterobacteria phage ATTK47 (0.0)	100	0	N
152	95680-95919	+	79	Hypothetical protein	*Escherichia* phage phiE142 (1e-48)	98.7	0	N
153	95962-96678	+	238	Hypothetical protein [PF00303; Thymidylat_synt; Thymidylate synthase]	*Escherichia* phage vB_EcoM_JS09 (1e-179)	100	0	N
154	96678-97577	+	299	Hypothetical protein	*Escherichia* phage APCEc01 (0.0)	100	0	N
155	97579-98127	+	182	Thymidylate kinase	Enterobacteria phage ATK47 (6e-130)	98.3	0	N
156	98226-99398	+	390	DNA repair protein	*Escherichia* phage p000v (0.0)	99.4	0	N
157	99391-99732	+	113	Head vertex assembly chaperone	*Shigella* phage JK45 (2e-76)	100	0	N
158	99742-101184	+	480	AAA family ATPase [PF03796; DnaB_C; DnaB-like helicase C terminal domain]	*Shigella* phage Shf125875 (0.0)	100	0	N

159	101273-101647	+	124	Hypothetical protein	*Escherichia* phage RB69 (6e-86)	100	0	N
160	101703-102020	+	105	Hypothetical protein	*Escherichia* phage vB_EcoM_PhAPEC2 (6e-73)	100	0	N
161	102017-102205	+	62	Dmd discriminator of mRNA degradation	*Escherichia coli* O157 typing phage 3 (1e-34)	98.3	0	N
162	102277-102645	+	122	Hypothetical protein	*Escherichia* phage vB_EcoM_JS09 (4e-85)	99.1	0	Y
163	102707-102955	+	82	Immunity to superinfection	*Escherichia* coli O157 typing phage 3 (2e-48)	100	0	N
164	103019-103312	+	97	Spackle	*Escherichia* phage RB69 (2e-65)	98.9	0	Y
165	103314-103964	+	216	Hypothetical protein	*Escherichia* phage vB_EcoM_WFbE185(2e-158)	100	0	N
166	103966-104163	+	65	Hypothetical protein	*Escherichia* phage RB69 (2e-39)	100	0	N
167	104183-104650	+	155	Hypothetical protein	*Escherichia* phage vB_EcoM_JS09 (8e-110)	100	0	N
168	104690-105712	+	340	DNA primase [IPR046392; PRIMASE_T4; DNA primase, bacteriophage T4]	*Escherichia* phage phiE142 (0.0)	100	0	N
169	105709-105906	-	65	Hypothetical protein	*Escherichia* phage vB_EcoM_G2469 (8e-35)	100	0	N
170	105997-106518	+	173	dCTP pyrophosphatase	*Escherichia* phage AV117 (2e-124)	98.8	0	N
171	106564-106800	+	78	Virion structural protein [PF16855; Soc; Small outer capsid protein]	*Shigella* phage SHSML-52-1 (3e-50)	100	0	N
172	107098-107346	+	82	Hypothetical protein	*Escherichia* phage APCEc01 (4e-52)	100	0	N
173	107343-107522	+	59	Hypothetical protein	*Escherichia* phage vB_EcoM_JS09 (2e-33)	100	0	N
174	107522-107986	+	154	Mrh transcription modulator under heat shock	*Escherichia* phage vB_EcoM_PhAPEC2 (8e-109)	100	0	N

175	107988-108167	+	59	Hypothetical protein	*Escherichia* phage vB_EcoM_G2285 (3e-33)	98.3	0	N
176	108164-108328	+	54	Hypothetical protein	*Escherichia* phage RB69 (6e-28)	100	0	N
177	108384-108965	+	193	Hypothetical protein [IPR043662 ; ModB-like ; NAD-protein ADP-ribosyltransferase ModB-like]	*Escherichia* phage vB_EcoM_G53 (6e-142)	100	0	N
178	109023-109631	+	202	ADP-ribosylase	*Escherichia* phage mobillu (1e-147)	98.5	0	N
179	109785-110531	+	248	Srd anti-sigma factor	*Escherichia* phage RB69 (9e-177)	98.7	0	N
180	110534-110845	+	103	Hypothetical protein	*Escherichia* phage vB_EcoM_JS09 (9e-69)	100	0	N
181	110842-112155	+	437	Dda-like helicase	*Escherichia* phage APCEc01 (0.0)	99.7	0	N
182	112165-112842	+	225	Exonuclease [PF16473; Rv2179c-like; 3'-5' exoribonuclease Rv2179c-like domain]	*Shigella* phage SHSML-52-1 (7e-166)	100	0	N
183	112909-113403	+	164	Hypothetical protein [PF17613; motB; Modifier of transcription]	*Escherichia* phage APCEc01 (2e-114)	97.5	0	N
184	113464-113928	+	154	MotB-like transcriptional regulator	*Escherichia* phage APCEc01 (5e-105)	97.4	0	N
185	113938-114357	+	139	MotB-like protein	*Escherichia* phage mogra (2e-95)	97.8	0	N
186	114417-114941	+	174	Hypothetical protein	*Escherichia* phage vB_EcoM_WFL6982 (7e-127)	100	0	N
187	114999-115226	+	75	cef modifier of supressor tRNAs	*Escherichia* phage HX01 (7e-47)	100	0	N
188	115226-115639	+	137	Hypothetical protein	*Escherichia* phage phiC120 (1e-94)	97	0	N
189	115639-115818	+	59	Hypothetical protein	*Escherichia* phage ChristianSchoenbein (1e-35)	98.3	0	N
190	115821-116246	+	141	Hypothetical protein	*Escherichia* phage ST0 (1e-95)	100	0	N

191	116310-118127	+	605	DNA topoisomerase II large subunit [IPR001241 ; Topo_IIA; DNA topoisomerase, type IIA]	*Shigella* phage SHSML-52-1 (0.0)	100	0	N
192	118170-119270	+	366	Hypothetical protein	*Escherichia* phage APCEc01 (2e-94)	97.7	0	N
193	119363-119563	+	66	Hypothetical protein	*Escherichia* phage F2(2e-36)	98.4	0	N
194	119576-121789	+	737	RIIA lysis inhibitor	*Shigella* phage SHSML-52-1	99.1	0	N
195	121799-122734	+	311	RIIA lysis inhibitor	*Escherichia* phage RB69 (0.0)	98.7	0	N
196	122775-123062	+	95	Hypothetical protein	*Escherichia* phage RB69 (3e-61)	98.9	0	N
197	123079-123555	+	158	DenB-like DNA endonuclease IV	*Escherichia* phage a20 (7e-113)	993	0	N
198	123624-123887	+	87	Hypothetical protein	*Escherichia* phage vB_EcoM_G53 (1e-56)	100	0	N
199	123967-124077	+	36	Hypothetical protein	*Escherichia* phage vB_EcoM_JS09 (2e-16)	100	0	N
200	124138-124338	+	66	Hypothetical protein	*Escherichia* phage vB_EcoM_JS09 (5e-41)	100	0	N
201	124415-124861	+	148	Ndd-like nucleoid disruption protein	*Escherichia* phage vB_EcoM_JS09 (3e-104)	100	0	N
202	124914-125060	+	48	Hypothetical protein	*Escherichia* phage p000y (2e-23)	100	0	N
203	125060-125200	+	46	Hypothetical protein	*Shigella* phage SHSML-52-1 (8e-21)	100	0	N
204	125205-126530	+	441	DNA topoisomerase II	*Escherichia* phage APCEc01 (0.0)	99.3	0	N
205	126716-126931	+	71	Hypothetical protein	*Escherichia* phage PNJ-6 (3e-41)	98.5	0	N
206	127036-127668	+	210	Hypothetical protein	*Escherichia* phage vB_EcoM-ZQ3 (2e-146)	99	0	N
207	127679-128008	+	109	Hypothetical protein	*Escherichia* phage APCEc01 (9e-74)	99	0	N

208	128005-128466	+	153	Hypothetical protein	*Escherichia* phage vB_EcoM_JS09 (1e-109)	100	0	N
209	128466-128747	+	93	Hypothetical protein	*Yersinia* phage phiD1 (2e-61)	100	0	N
210	128924-129043	+	39	Hypothetical protein	*Escherichia* phage vB_EcoM_JS09 (2e-17)	100	0	N
211	129033-129332	+	99	Hypothetical protein	*Escherichia* phage HX01 (5e-62)	95.9	0	N
212	129322-129483	+	53	Hypothetical protein	*Escherichia* phage vB_Eco_NicPhage(6e-28)	100	0	N
213	129530-129802	+	90	Hypothetical protein [PF09010; AsiA; Anti-Sigma Factor A]	*Shigella* phage SSE1 (4e-55)	100	0	N
214	129803-130462	-	219	Holin [PF11031; Phage_holin_T; Bacteriophage T holin]	*Yersinia* phage phiD1 (1e-159)	100	0	N
215	130472-131023	-	183	Putative tail fiber assembly protein	*Escherichia* phage vB_EcoM_G2469 (4e-128)	98.9	0	N
216	131054-134245	-	1063	Tail fibers protein	*Shigella* phage ESh17 (0.0)	90	0	N
217	134254-134919	-	221	Hinge connector of long tail fiber, distal connector	*Escherichia* phage vB_Eco_F31 (3e-158)	99.5	0	N
218	134982-136109	-	375	Long-tail fiber protein	*Escherichia* phage JN02 (0.0)	99.2	0	N
219	136118-139996	-	1292	Putative long tail fiber proximal subunit	*Escherichia* phage 55 (0.0)	94.3	0	N
220	140100-141017	+	305	Hypothetical protein [PF09293; RNaseH_C; T4 RNase H, C terminal]	*Escherichia* phage ST0 (0.0)	100	0	N
221	141025-141294	+	89	Transcriptional regulator	*Escherichia* phage RB69 (5e-56)	100	0	N
222	141272-141610	+	112	Late promoter transcription accessory protein	*Escherichia* phage PaulHMueller (3e-74)	100	0	N
223	141607-142260	+	217	DNA helicase loader	*Shigella* phage JK45 (2e-156)	100	0	N
224	142375-143274	+	299	Single-stranded DNA-binding protein [PF08804; gp32; gp32 DNA binding protein like]	*Escherichia* phage F2 (0.0)	100	0	N

225	143388-143780	+	130	Hypothetical protein	*Escherichia* phage phi25-307 (1e-88)	99.2	0	N
226	143842-144090	+	82	DUF5417 domain-containing protein	*Shigella* phage SHSML-52-1 (2e-50)	95.1	0	N
227	144093-144680	+	195	Dihydrofolate reductase	*Shigella* phage SHSML-52-1 (4e-139)	97.9	0	N
228	144677-145537	+	286	dTMP (thymidylate) synthase [PF00303; Thymidylat_synt; Thymidylate synthase]	*Escherichia* phage PNJ-6 (0.0)	99.3	0	N
229	145539-145793	+	84	Hypothetical protein [PF17600; DUF5496; Family of unknown function (DUF5496)]	*Escherichia* phage F2 (7e-54)	100	0	N
230	145881-148136	+	751	NrdA-like aerobic NDP reductase large subunit [IPR039718; Rrm1 ; Ribonucleoside-diphosphate reductase large subunit]	*Escherichia* phage ST0 (0.0)	99	0	N
231	148190-149329	+	379	ribonucleotide reductase class Ia beta subunit [PF00268; Ribonuc_red_sm; Ribonucleotide reductase, small chain]	*Escherichia* phage F2 (0.0)	98.9	0	N
232	149356-149766	+	136	Putative endonuclease [IPR016413Phage_T4_DenA_endoDNaseII Bacteriophage T4, DenA, endonuclease II]	*Escherichia* phage vB_EcoM_MM02 (4e-94)	99.2	0	N
233	149823-150947	+	374	Putative RNA ligase 1 and tail fiber attachment catalyst [IPR012648 ; Rnl1 ; T4 RNA ligase 1]	*Escherichia* phage mobillu (0.0)	99.2	0	N
234	151010-151510	+	166	Inhibitor of host transcription	*Shigella* phage JK45 (8e-119)	99.4	0	N
235	151498-151854	+	118	Rz-like spanin	*Shigella* phage Shf125875 (6e-76)	99.1	0	N
236	151851-152141	+	96	Hypothetical protein	*Escherichia* phage vB_EcoM_G2285(3e-6397.9)	97.9	0	Y
237	152138-152356	+	72	Hypothetical protein	*Escherichia* phage phiC120 (8E-45)	100	0	N
238	152419-152718	+	99	Hypothetical protein	*Escherichia* phage phiC120 (1E-34)	66.6	0	N
239	152718-152909	+	63	Hypothetical protein	*Escherichia* phage vB_EcoM-172859UKE1 (3e-37)	98.4	0	N

240	152906-153805	+	299	Polynucleotide 5'-kinase and 3'-phosphatase [IPR044493 ; PNKP_C_HAD ; Polynucleotide kinase PNKP, C-terminal phosphatase domain]	*Escherichia* phage mobillu (0.0)	100	0	N
241	153806-153997	+	63	Hypothetical protein	*Escherichia* phage vB_EcoM_TU01(6e-37)	100	0	N
242	153987-154202	+	71	Hypothetical protein	*Escherichia* phage vB_EcoM_JS09 (4e-44)	100	0	N
243	154210-154485	+	91	Hypothetical protein	*Escherichia* phage vB_EcoM_G2285 (7e-58)	98.9	0	N
244	154546-154782	+	78	Hypothetical protein	*Escherichia* phage vB_EcoM_JS09 (4e-49)	100	0	N
245	154769-154894	+	41	Hypothetical protein	*Escherichia* coli O157 typing phage 3 (3e-19)	100	0	N
246	154903-155895	+	330	Phospho-2-dehydro-3-deoxyheptonate aldolase [IPR006219; DAHP_synth_1 DAHP synthase, class 1]	*Shigella* phage Shf125875 (0.0)	99.7	0	N
247	155895-156476	+	193	Deoxycytidylate deaminase [IPR015517 ; dCMP_deaminase-rel; Deoxycytidylate deaminase-related]	*Escherichia* phage vB_EcoM_PhAPEC2 (5e-141)	100	0	N
248	156478-156774	+	98	Hypothetical protein [PF10902; WYL_2; WYL_2, Sm-like SH3 beta-barrel fold]	*Shigella* phage Shf125875 (2e-64)	100	0	N
249	156832-157164	+	110	Head assembly chaperone protein [PF00166; Cpn10; Chaperonin 10 Kd subunit]	*Escherichia* phage vB_EcoM_NBG1 (2e-71)	100	0	N
250	157289-157537	+	82	Head assembly chaperone protein [IPR034688 ; Linr3 ; Lysis inhibition accessory protein rIII]	*Escherichia* phage APCEc01 (2e-71)	100	0	N
251	157832-158011	+	59	Hypothetical protein	*Escherichia* phage a20 (2e-31)	98.3	0	N
252	158141-158509	+	122	Hypothetical protein	*Escherichia* phage vB_EcoM_JS09 (1e-84)	100	0	N
253	158584-158949	+	121	Hypothetical protein	*Escherichia* phage APCEc01 (1e-85)	100	0	N
254	158985-159599	+	204	Hypothetical protein	*Shigella* phage JK45 (2e-151)	100	0	N
255	159654-159851	+	65	Hypothetical protein	*Escherichia* phage APCEc01 (6e-39)	100	0	N

256	159841-160059	+	72	Hypothetical protein	*Escherichia* phage APCEc01 (6e-39)	100	0	N
257	160052-160510	+	152	Hypothetical protein	*Escherichia* coli O157 typing phage 3 (2e-108)	100	0	N
258	160507-161322	+	271	Hypothetical protein	*Escherichia* phage vB_EcoM-ZQ3 (0.0)	98.1	0	N
259	161332-161601	+	89	Hypothetical protein	*Escherichia* phage vB_EcoM_JS09 (6e-59)	100	0	N
260	161598-163091	+	497	DNA ligase [PF01068; DNA_ligase_A_M; ATP dependent DNA ligase domain]	*Escherichia* phage vB_EcoM-RPN187(0.0)	100	0	N
261	163091-163279	+	62	Hypothetical protein	*Escherichia* phage vB_EcoM_G53 (1e-35)	95.1	0	N
262	163335-165422	+	695	RNA polymerase ADP-ribosylase [IPR016225 ; Phage_T4_Alt-like; NAD--protein ADP-ribosyltransferase Alt-like]	*Escherichia* phage moskry (0.0)	99.5	0	N
263	165481-165774	+	97	Hypothetical protein	*Escherichia* phage vB_EcoM_PhAPEC2 (5e-63)	97.9	0	N
264	165808-166770	-	320	Tail tube	*Shigella* phage SHSML-52-1 (0.0)	99.6	0	N
265	166770-167879	-	369	Baseplate tail tube cap	*Escherichia* phage HX01 (0.0)	99.4	0	N
266	167888-168094	-	68	Baseplate hub	*Escherichia* phage F2 (3e-36)	100	0	N
