# Supplementary material for: Characterization of Five Lytic Bacteriophages as New Members of the Genus Mosigvirus, Infecting Multidrug-Resistant Shiga Toxin-Producing Escherichia coli and Their Antibiofilm Activity
Source: Viruses. 2025 Nov 13;17(11):1501. doi: 10.3390/v17111501 (PMC12656860; doi:10.3390/v17111501)
Supplement: Supplementary file 1 [file viruses-17-01501-s001.zip › Table S3.pdf]

Table S3. Features of predicted ORFs and their homology to STEC phage ΦJ.

Gene product				Putative function [Conserved domain]	Best match organism (E-value)	Identity (%)	Predicted TMHHM and signal peptide	
ORF No.	Range	Strand	Length (AA)				TMHHM	SignalP
1	2-337	+	111	Hypothetical protein	*Escherichia* phage vB_EcoM_JS09 (3e-74)	100	0	N
2	334-681	+	115	Hypothetical protein	*Escherichia* phage F2 (2e-78)	100	0	Y
3	674-1141	+	155	Phosphatase	*Escherichia* phage Av-05 (4e-110)	100	0	N
4	1138-1350	+	70	Hypothetical protein	*Escherichia* phage vB_EcoM_IME537 (5e-45)	100	0	N
5	1347-1553	+	68	Hypothetical protein	*Escherichia* phage F2 (4e-33)	100	0	N
6	1550-1732	+	60	Hypothetical protein	*Escherichia* coli O157 typing phage 3 (3e-33)	100	0	N
7	1742-2323	+	193	Tk thymidine kinase [PF00265; TK; Thymidine kinase]	*Escherichia* coli O157 typing phage 3 (1e-140)	99.4	0	N
8	2351-2563	+	70	Hypothetical protein	*Escherichia* phage HX01 (3e-41)	98.5	0	N
9	2576-2878	+	100	Lysis inhibition regulator	*Escherichia* phage moskry (1e-67)	99	1	Y
10	2980-3159	+	59	Hypothetical protein	*Escherichia* phage vB_EcoM_PhAPEC2 (6e-35)	100	0	N
11	3167-3331	+	54	Hypothetical protein	*Escherichia* phage vB_EcoM-ZQ3 (2e-32)	100	0	N
12	3328-3423	+	31	Hypothetical protein	*Escherichia* phage vB_EcoM_JS09 (1e-12)	100	1	N
13	3423-3638	+	71	Hypothetical protein	*Escherichia* phage vB_EcoM-RPN187 (1e-43)	100	0	N
14	3684-3806	+	40	Hypothetical protein	*Escherichia* phage ST0 (4e-18)	100	1	N

15	3803-3982	+	59	Helicase loader	*Escherichia* phage ST0 (3e-34)	100	0	N
16	3984-4514	+	176	Hypothetical protein	*Escherichia* phage vB_EcoM-RPN187 (2e-125)	98.8.	0	N
17	4524-4997	+	157	Membrane protein	*Escherichia* phage ST0 (5e-107)	96.8	2	N
18	4997-5983	+	328	Nucleotidyltransferase [PF10127; RlaP; RNA repair pathway DNA polymerase beta family]	*Escherichia* phage ST0 (0.0)	99.7	0	N
19	6015-6281	+	88	Hypothetical protein	*Escherichia* phage vB_EcoM_G2285 (5e-57)	100	1	Y
20	6401-7381	+	326	Hypothetical protein [IPR003593 ; AAA+_ATPase ; AAA+ ATPase domain]	*Escherichia* phage vB_EcoM_JS09 (0.0)	100	0	N
21	7520-7807	+	95	Hypothetical protein	*Escherichia* phage vB_EcoM_SQ17(5e-61)	100	0	N
22	7866-8393	+	175	Hypothetical protein	*Shigella* phage phi25-307 (2e-122)	99.4	0	N
23	8456-9451	+	331	Hypothetical protein	*Escherichia* phage ST0 (0.0)	99.4	0	N
24	9507-10466	+	319	Hypothetical protein	*Shigella* phage SSE1 (0.0)	98.7	0	N
25	10529-11479	+	316	Hypothetical protein	*Escherichia* phage GADS24 (0.0)	98.1	0	N
26	11479-11784	+	101	Hypothetical protein	*Escherichia* phage vB_EcoM_JS09 (4e-69)	100	0	N
27	11784-12197	+	137	Hypothetical protein	*Escherichia* phage p000y (1e-93)	97.8	2	Y
28	12190-12453	+	87	Putative thioredoxin [PF00462; Glutaredoxin; Glutaredoxin]	*Escherichia* phage RB69 (1e-57)	100	0	N
29	12450-12806	+	118	Hypothetical protein	*Shigella* phage Shf125875 (2e-79)	100	0	N
30	12935-13105	+	56	Hypothetical protein	*Escherichia* phage vB_EcoM_MM02 (2e-32)	98.2	0	N

31	13107-13520	+	137	Protease inhibitor [PF10465; Inhibitor_I24; PinA peptidase inhibitor]	*Escherichia* phage GADS24 (6e-95)	100	0	N
32	13530-13709	+	59	Hypothetical protein	*Escherichia* phage vB_EcoM_TU01 (3e-35)	98.3	0	N
33	13748-14221	+	157	Endonuclease VII [PF02945 ; Endonuclease_7 ; Recombination endonuclease VII]	*Escherichia* phage vB_EcoM_JS09 (6e-113)	100	0	N
34	14218-16035	+	605	Anaerobic ribonucleoside reductase large subunit [PF13597 ; NRDD ; Anaerobic ribonucleoside-triphosphate reductase]	*Escherichia* phage HX01 (0.0)	99.8	0	N
35	16032-16502	+	156	Anaerobic NTP reductase small subunit [IPR007197 ; rSAM ; Radical SAM]	*Escherichia* phage vB_EcoM-ZQ3 (6e-111)	98.7	0	N
36	16495-16608	+	37	Ribonucleotide reductase of class III activating protein	*Escherichia* phage HX01 (2e-17)	100	0	N
37	16617-16829	+	70	Hypothetical protein	*Escherichia* phage vB_EcoM_G2285 (9e-40)	98.5	1	N
38	16832-17140	+	102	Putative glutaredoxin	*Escherichia* phage 55 (1e-67)	100	0	N
39	17306-17488	+	60	Hypothetical protein	*Escherichia* phage vB_EcoM_JS09 (7e-36)	100	0	N
40	17485-17733	+	82	Hypothetical protein	*Escherichia* phage vB_EcoM_SQ17 (5e-49)	98.7	0	N
41	17741-18034	+	97	Hypothetical protein	*Escherichia* phage vB_EcoM_JS09 (8e-63)	100	0	N
42	18042-18176	+	44	Hypothetical protein [PF17583; DUF5484; Family of unknown function (DUF5484)]	*Shigella* phage Shf125875 (1e-23)	100	0	N
43	18173-18373	+	66	Hypothetical protein [PF17593; DUF5490; Family of unknown function (DUF5490)]	*Escherichia* phage vB_EcoM_JS09 (2e-41)	100	0	N
44	18437-18676	+	79	Hypothetical protein	*Escherichia* phage vB_EcoM_JS09 (1e-49)	98.7	0	N
45	18676-19077	+	133	Hypothetical protein	*Escherichia* phage F2 (6e-82)	100	0	N
46	19074-19301	+	75	Hypothetical protein	*Escherichia* phage ST0 (4e-44)	98.6	0	N

47	19298-19567	+	89	Hypothetical protein	*Escherichia* phage moskry(3e-44)	98.8	0	N
48	19640-20197	+	185	RNA polymerase sigma factor [IPR046386 ; T4_sigma-like_factor ; RNA polymerase sigma-like factor]	*Escherichia* phage RB69 (7e-134)	99.4	0	N
49	20187-20396	+	69	Hypothetical protein [PF17595; DUF5491; Family of unknown function (DUF5491)]	*Escherichia* phage APCEc01 (5e-42)	100	0	N
50	20398-20721	+	107	Hypothetical protein [PF10849; DUF2654; Protein of unknown function (DUF2654)]	*Escherichia* phage p000v (1e-68)	99	0	N
51	20942-21115	+	57	Hypothetical protein [PF17588 ; DUF5486 ; Family of unknown function]	*Escherichia* phage APCEc01 (2e-33)	100	0	N
52	21185-22204	+	339	Sbc-D-like subunit of palindrome specific endonuclease [PF00149; Metallophos; Calcineurin-like phosphoesterase]	*Escherichia* ST0 (0.0)	99.7	0	N
53	22201-22458	+	85	Hypothetical protein	*Escherichia* phage vB_EcoM-ZQ3 (6e-55)	100	0	N
54	22445-22684	+	79	Hypothetical protein [PF17589; DUF5487; Family of unknown function (DUF5487)]	*Escherichia* phage 308Ecol101PP (4e-50)	100	0	N
55	22681-24369	+	562	recombination endonuclease subunit [PF13476; AAA_23; AAA domain]	*Escherichia* phage vB_EcoM-ZQ3 (0.0)	99.6	0	N
56	24424-24612	+	62	Hypothetical protein [PF17470; Gp45_2; Phage gene product 45.2]	*Escherichia* phage RB69 (6e-38)	100	0	N
57	24625-25041	+	138	RNA polymerase binding [PF10789; Phage_RpbA; Phage RNA polymerase binding, RpbA]	*Escherichia* phage RB69 (2e-98)	100	0	N
58	25084-25770	+	228	Putative sliding clamp [PF02916 ; DNA_PPF ; DNA polymerase processivity factor]	*Escherichia* phage vB_EcoM_WFL6982 (4e-164)	99.5	0	N
59	25846-26808	+	320	Clamp loader of DNA polymerase [PF00004 ; AAA ; ATPase family associated with various cellular activities (AAA)]	*Shigella* phage Shf125875	99.6	0	N
60	26810-27373	+	187	DNApol clamp loader large subunit [PF16790; Phage_clamp_A; Bacteriophage clamp loader A subunit]	*Escherichia* phage AlbertHofmann (1e-133)	99.4	0	N
61	27376-27744	+	122	Translation repressor [PF01818; Translat_reg; Bacteriophage translational regulator]	*Escherichia* phage RB69 (5e-84)	100	0	N

62	27826-30537	+	903	DNA polymerase [PF00136; DNA_pol_B; DNA polymerase family B]	*Escherichia* phage vB_EcoM_JS09 (0.0)	99.8	0	N
63	30578-31213	+	211	arabinose 5-phosphate isomerase [PF01380; SIS; SIS domain]	*Escherichia* phage F2 (9e-153)	99.5	0	N
64	31210-31353	+	47	Hypothetical protein	*Shigella* phage Shf125875 (1e-22)	98	1	N
65	31395-33080	+	561	Hypothetical protein [PF00483; NTP_transferase; Nucleotidyl transferase]	*Escherichia* phage vB_EcoM_JS09 (0.0)	100	0	N
66	33080-33466	+	128	Phosphoheptose isomerase	*Escherichia* phage UGJNEcP1 (9e-91)	100	0	N
67	33521-34681	+	386	Peptidase U32 [PF01136; Peptidase_U32; Peptidase family U32]	*Escherichia* phage 308Ecol101PP (0.0)	99.7	0	N
68	34678-34863	+	61	Hypothetical protein	*Escherichia* phage ChristianSchoenbein (2e-35)	100	0	N
69	34959-35675	+	238	Hypothetical protein [PF00303; Thymidylat_synt; Thymidylate synthase]	*Escherichia* phage vB_EcoM_TU01 (2e-179)	100	0	N
70	35675-36574	+	299	RNA polymerase binding protein	*Escherichia* phage HX01 (0.0)	99.6	0	N
71	36577-37125	+	182	Thymidylate kinase	*Escherichia* phage F2 (2e-130)	99.4	0	N
72	37225-38397	+	390	RecA-like recombination protein [PF21134 ; T4_UVSX_C ; Bacteriophage T4, Recombination and repair protein, C-terminal domain]	*Escherichia* phage mogra (0.0)	99.7	0	N
73	38390-38731	+	113	Head vertex assembly chaperone [PF11113; Phage_head_chap; Head assembly gene product]	*Shigella* phage Shf125875 (3e-76)	99.7	0	N
74	38741-40183	+	480	AAA family ATPase [PF03796; DnaB_C; DnaB-like helicase C terminal domain]	*Escherichia* phage ST0 (0.0)	99.7	0	N
75	40272-40646	+	124	Hypothetical protein	*Escherichia* phage HX01 (5e-86)	100	0	N
76	40702-41019	+	105	Hypothetical protein	*Escherichia* phage OLB35 (2e-75)	99	0	N
77	41016-41207	+	63	Dmd discriminator of mRNA degradation [PF17587 ; Dmd ; Discriminator of mRNA degradation]	*Escherichia* phage F2 (1e-35)	98.4	0	N

78	41209-41421	+	70	Hypothetical protein	*Escherichia* phage RB69 (6e-44)	100	0	N
79	41482-41850	+	122	Hypothetical protein [PF17578; DUF5481; Family of unknown function (DUF5481)]	*Escherichia* phage vB_EcoM_JS09 (5e-85)	99.1	0	Y
80	41912-42160	+	82	Immunity to superinfection [PF14373; Imm_superinfect; Superinfection immunity protein]	*Escherichia* coli O157 typing phage 3(2e-48)	100	2	N
81	42224-42517	+	97	Spackle periplasmic [PF17979; zf-CRD; Cysteine rich domain with multizinc binding regions]	*Escherichia* phage RB69 (2e-65)	99.88	0	Y
82	42519-43169	+	216	Hypothetical protein	*Escherichia* phage PTK (2e-158)	100	0	N
83	43171-43368	+	65	Hypothetical protein	*Escherichia* phage vB_EcoM_JS09 (2e-39)	100	0	N
84	43388-43855	+	155	Hypothetical protein	*Escherichia* phage vB_EcoM_JS09 (8e-110)	100	0	N
85	43895-44917	+	340	DNA primase [PF08275; Toprim_N; DNA primase catalytic core, N-terminal domain]	*Shigella* phage Shf125875 (0.0)	100	0	N
86	44914-45111	-	65	Hypothetical protein	*Escherichia* phage ST0 (3e-35)	100	1	N
87	45201-45722	+	173	nucleoside triphosphate pyrophosphohydrolase [PF08761; dUTPase_2; dUTPase]	*Shigella* phage JK45 (6e-125)	99.4	0	N
88	45768-46004	+	78	Virion structural protein [PF16855; Soc; Small outer capsid protein]	*Escherichia* phage F2 (4e-49)	98.7	0	N
89	46304-46552	+	82	Hypothetical protein	*Escherichia* phage moha (3e-51)	97.5	0	N
90	46549-46728	+	59	Hypothetical protein	*Escherichia* phage ST0 (6e-32)	96.61	0	N
91	46728-47192	+	154	Mrh transcription modulator under heat shock	*Escherichia* phage phiE142 (5e-109)	100	0	N
92	47209-47373	+	54	molybdenum ABC transporter, periplasmic molybdenum-binding protein	*Escherichia* phage vB_EcoM_TU01 (2e-30)	100	0	N
93	47370-47534	+	54	Hypothetical protein	*Escherichia* phage vB_EcoM_WFbE185 (8e-28)	98.1	0	N
94	47590-48171	+	193	RNA polymerase ADP-ribosylase [IPR043662 ; ModB-like ; NAD-protein ADP-ribosyltransferase	*Escherichia* phage vB_EcoM_JS09 (6e-140)	98.96	0	N

				ModB-like]				
95	48229-48837	+	202	RNA polymerase ADP-ribosylase [IPR043662 ; ModB-like ; NAD-protein ADP-ribosyltransferase ModB-like]	*Escherichia* phage F2 (2e-149)	100	0	N
96	48989-49735	+	248	Srd anti-sigma factor	*Escherichia* phage RB69 (1e-178)	100	0	N
97	49738-50049	+	103	Hypothetical protein	*Escherichia* phage F2 (2e-68)	99	0	N
98	50046-51359	+	437	Dda-like helicase [PF18343; SH3_14; Dda helicase SH3 domain]	*Shigella* phage phi25-307 (0.0)	100	0	N
99	51369-52046	+	225	Exonuclease [PF16473; DUF5051; 3' exoribonuclease, RNase T-like]	*Shigella* phage phi25-307 (7e-166)	100	0	N
100	52113-52607	+	164	Transcriptional regulator [PF17613; motB; Modifier of transcription]	*Escherichia* phage vB_EcoM-ZQ3 (5e-118)	100	0	N
101	52669-53124	+	151	Modifier of transcription [PF17613; motB; Modifier of transcription]	*Escherichia* phage vB_EcoM_SQ17(8e-108)	100	0	N
102	53134-53553	+	139	Transcriptional regulator [PF17613; motB; Modifier of transcription]	*Escherichia* phage vB_EcoM_ZQ3 (3e-96)	100	0	N
103	53613-54137	+	174	Hypothetical protein	*Escherichia* phage HX01 (2e-126)	100	0	N
104	54195-54422	+	75	protein Cef	*Escherichia* phage S143_2 (8e-47)	100	0	N
105	54422-54835	+	137	Putative RNA metabolism moderator	*Escherichia* phage vB_EcoM-ZQ3 (2e-97)	100	0	N
106	54835-55014	+	59	Hypothetical protein [PF09723; Zn-ribbon_8; Zinc ribbon domain]	*Escherichia* phage ChristianSchoenbein (2e-36)	98.3	0	N
107	55017-55442	+	141	Hypothetical protein	*Escherichia* phage ST0 (1e-95)	100	0	N
108	55506-57323	+	605	Topoisomerase II subunit [PF00204; DNA_gyraseB]	*Escherichia* phage vB_EcoM-ZQ3(0.0)	99.8	0	N
109	57366-58466	+	366	Hypothetical protein	*Escherichia* phage F2 (0.0)	99.5	0	N

110	58559-58759	+	66	Hypothetical protein	*Escherichia* phage F2 (4e-37)	98.4	0	N
111	58772-60985	+	737	rIIA lysis inhibitor	*Escherichia* phage PHB12 (0.0)	99.7	0	N
112	60995-61930	+	311	Hypothetical protein	*Escherichia* phage vB_EcoM-RPN187(0.0)	99.6	0	N
113	61972-62259	+	95	Hypothetical protein	*Escherichia* phage vB_EcoM_JS09 (4e-61)	98.95	0	N
114	62276-62752	+	158	endonuclease IV	*Escherichia* phage S143_2 (1e-112)	100	0	N
115	62821-63084	+	87	Hypothetical protein	*Escherichia* phage RB69 (6e-57)	100	0	N
116	63164-63271	+	35	Hypothetical protein	*Escherichia* phage p000y (9e-16)	100	1	Y
117	63332-63532	+	66	Hypothetical protein	*Escherichia* phage SF (1e-40)	100	0	N
118	63609-64055	+	148	Ndd-like nucleoid disruption protein [PF06591; Phage_T4_Ndd; T4-like phage nuclear disruption protein (Ndd)]	*Escherichia* phage ST0 (4e-104)	100	0	N
119	64108-64257	+	49	Hypothetical protein	*Escherichia* phage vB_EcoM_JS09(3e-24)	100	1	N
120	64399-65724	+	441	DNA topoisomerase II [PF00521; DNA_topoisoIV; DNA gyrase/topoisomerase IV, subunit A]	*Escherichia* phage ST0 (0.0)	100	0	N
121	65910-66125	+	71	Hypothetical protein	*Escherichia* phage PNJ-6 (3e-41)	100	0	N
122	66229-66861	+	210	Activator of middle period transcription [PF09114; MotA_activ; Transcription factor MotA, activation domain]	*Escherichia* phage vB_EcoM-ZQ3 (2e-147)	99.5	0	N
123	66872-67216	+	114	Hypothetical protein	*Escherichia* phage ST0 (6e-78)	100	0	N
124	67213-67674	+	153	Hypothetical protein	*Escherichia* phage ST2 (7e-110)	99.3	0	N
125	67674-67955	+	93	Anti-restriction nuclease	*Escherichia* phage PNJ-6 (9e-61)	98.9	0	N

126	68132-68251	+	39	Hypothetical protein	*Escherichia* phage vB_EcoM_JS09 (2e-17)	100	0	N
127	68241-68540	+	99	Hypothetical protein	*Escherichia* phage vB_EcoM_TU01 (4e-66)	100	0	N
128	68530-68691	+	53	Hypothetical protein [PF09114 ; MotA_activ ; Transcription factor MotA, activation domain]	*Shigella* phage phi25-307 (2e-49)	98.7	0	N
129	68738-69010	+	90	Hypothetical protein [PF09010; AsiA; Anti-Sigma Factor A]	*Escherichia* phage HX01 (4e-56)	100	0	N
130	69011-69670	-	219	Holin [PF11031; Phage_holin_T; Bacteriophage T holin]	*Shigella* phage JK45 (8e-159)	99	1	N
131	69680-70231	-	183	Tail fiber assembly protein [PF02413; Caudo_TAP; Caudovirales tail fibre assembly protein, lambda gpK]	*Escherichia* phage F2 (9e-129)	99.4	0	N
132	70259-73696	-	1145	Putative long tail fiber protein [PF20744 ; gp37_trimer ; Tail fibre protein gp37 trimerization region]	*Escherichia* phage moha (0.0)	92.3	0	N
133	73705-74370	-	221	Hinge connector of long tail fiber protein distal connector [PF03903; Phage_T4_gp36; Phage T4 tail fibre]	*Escherichia* phage UGJNEcP1 (5e-158)	99.5	0	N
134	74433-75560	-	375	Hinge connector of long tail fiber, proximal connector [PF15711; ILEI; Interleukin-like EMT inducer]	*Escherichia* phage vB_EcoM_SA79RD (0.0)	99.2	0	N
135	75569-79444	-	1291	Long tail fiber proximal subunit [PF21446 ; Gp34_C ; Long-tail fiber proximal subunit, C-terminal domain]	*Escherichia* phage moskry (0.0)	99.1	0	N
136	79548-80465	+	305	Ribonuclease H [PF09293; RNaseH_C; T4 RNase H, C terminal]	*Shigella* phage SHSML-52-1 (0.0)	100	0	N
137	80473-80742	+	89	Transcriptional regulator [PF11126; Phage_DsbA; Transcriptional regulator DsbA]	*Escherichia* phage RB69 (5e-56)	100	0	N
138	80720-81058	+	112	Late promoter transcription accessory protein	*Escherichia* phage PaulHMueller (3e-74)	99.1	0	N
139	81055-81708	+	217	Hypothetical protein [PF08993; T4_Gp59_N; T4 gene Gp59 loader of gp41 DNA helicase]	*Escherichia* phage mobillu (4e-154)	99	0	N
140	81826-82725	+	299	Putative single-stranded DNA binding protein [PF08804; gp32; gp32 DNA binding protein like]	*Escherichia* phage vB_EcoM_G2285 (0.0)	100	0	N
141	82840-83091	+	83	Hypothetical protein	*Escherichia* phage moskry (2e-54)	100	0	N

142	83102-83494	+	130	Hypothetical protein [PF03197; FRD2; Bacteriophage FRD2 protein]	Enterobacteria phage ATK47 (2e-88)	99.2	0	N
143	83556-83804	+	82	Hypothetical protein [PF17438; DUF5417; Family of unknown function (DUF5417)]	*Shigella* phage SHSML-52-1 (4e-53)	100	0	N
144	83807-84394	+	195	Dihydrofolate reductase [PF00186; DHFR_1; Dihydrofolate reductase]	*Shigella* phage SHSML-52-1 (3e-139)	98.4	0	N
145	84391-85251	+	286	Putative thymidylate synthase [PF00303; Thymidylat_synt; Thymidylate synthase]	*Escherichia* phage vB_EcoM_MM02 (0.0)	99.6	0	N
146	85253-85507	+	84	Hypothetical protein [PF17600; DUF5496; Family of unknown function (DUF5496)]	*Escherichia* coli phage mobillu (3e-53)	100	0	N
147	85595-87850	+	751	Ribonucleoside-diphosphate reductase 1 subunit alpha [PF02867; Ribonuc_red_lgC; Ribonucleotide reductase, barrel domain]	*Escherichia* phage vB_EcoM_WFbE185 (0.0)	99.8	0	N
148	87904-89082	+	392	Putative ribonucleoside-diphosphate reductase 1 subunit beta [PF00268; Ribonuc_red_sm; Ribonucleotide reductase, small chain]	*Escherichia* phage vB_EcoM_G53 (8e-95)	98.7	0	N
149	89109-89519	+	136	Putative endonuclease [PF00483 ; NTP_transferase ; Nucleotidyl transferase]	*Escherichia* phage vB_EcoM_MM02 (5e-95)	99.2	0	N
150	89575-90699	+	374	RNA ligase [PF09511; RNA_lig_T4_1; RNA ligase]	*Escherichia* phage FL18 (0.0)	99.7	0	N
151	90761-91261	+	166	Inhibitor of host transcription [PF17527; ALC; Phage ALC protein]	*Shigella* phage JK45 (3e-119)	100	0	N
152	91249-91605	+	118	Hypothetical protein	*Escherichia* phage vB_EcoM_G2285 (1e-76)	99.1	1	Y
153	91602-91892	+	96	Rz-like spanin	*Escherichia* phage APCEc01 (9e-65)	98.9	0	Y
154	91889-92107	+	72	Hypothetical protein	*Escherichia* phage phiC120 (8e-45)	100	0	N
155	92165-92464	+	99	Hypothetical protein	*Escherichia* phage APCEc01 (8e-63)	98.9	0	N
156	92464-92655	+	63	Hypothetical protein	*Escherichia* phage APCEc01 (2e-37)	100	0	N
157	92652-93551	+	299	Polynucleotide 5'-kinase and 3'-phosphatase [PF13671; AAA_33; AAA domain]	*Escherichia* phage mobillu (0.0)	99.6	0	N

158	93552-93743	+	63	Hypothetical protein	*Escherichia* phage APCEc01 (1e-37)	100	0	N
159	93733-93948	+	71	Hypothetical protein	*Escherichia* phage vB_EcoM_JS09(4e-44)	100	0	N
160	93956-94231	+	91	Hypothetical protein	*Escherichia* phage p000y (3e-58)	100	0	N
161	94293-94529	+	78	Hypothetical protein	*Escherichia* phage vB_EcoM_JS09(4e-49)	100	0	N
162	94529-94642	+	37	Hypothetical protein	*Escherichia* coli O157 typing phage 3 (2e-14)	94.4	0	N
163	94651-95643	+	330	dAHP synthase, class 1 [PF00793; DAHP_synth_1; DAHP synthetase I family]	*Escherichia* phage vB_EcoM_JS09 (0.0)	98.79	0	N
164	95643-96224	+	193	dCMP deaminase [PF00383; dCMP_cyt_deam_1; Cytidine and deoxycytidylate deaminase zinc-binding region]	*Shigella* phage Shf125875 (5e-141)	100	0	N
165	96226-96522	+	98	Hypothetical protein [PF10902 ; WYL_2 ; WYL_2, Sm-like SH3 beta-barrel fold]	*Escherichia* phage APCEc01 (7e-64)	97.9	0	N
166	96580-96912	+	110	Head assembly chaperone protein [PF00166; Cpn10; Chaperonin 10 Kd subunit]	*Escherichia* phage vB_EcoM-ZQ3 (3e-71)	99	0	N
167	97037-97285	+	82	Hypothetical protein	*Escherichia* phage vB_EcoM-RPN187 (4e-51)	97.5	0	N
168	97554-97733	+	59	Hypothetical protein	*Escherichia* phage a20 (2e-31)	98.3	0	N
169	97866-98234	+	122	Hypothetical protein [PF06019; Phage_30_8; Phage GP30.8 protein]	*Escherichia* phage vB_EcoM_JS09 (2e-83)	99.1	0	N
170	98309-98674	+	121	Hypothetical protein [PF06919; Phage_T4_Gp30_7; Phage Gp30.7 protein]	*Escherichia* phage vB_EcoM_JS09 (9e-86)	100	0	N
171	98710-99324	+	204	Hypothetical protein	*Escherichia* phage 308Ecol101P (4e-149)	99.5	0	N
172	99379-99576	+	65	Hypothetical protein	*Escherichia* phage vB_EcoM_JS09 (1e-38)	100	0	N
173	99566-99778	+	70	Hypothetical protein	*Escherichia* phage 55 (9e-41)	97.1	0	N

174	99771-100229	+	152	Hypothetical protein [PF08010; Phage_30_3; Bacteriophage protein GP30.3]	*Shigella* phage Shf125875 (5e-108)	98.6	0	N
175	100226-101041	+	271	Hypothetical protein	*Escherichia* phage JN02 (0.0)	99.2	0	N
176	101051-101320	+	89	Hypothetical protein	*Escherichia* phage vB_EcoM_JS09 (3e-59)	98.8	0	N
177	101317-102810	+	497	ATP-dependent DNA ligase [PF01068; DNA_ligase_A_M; ATP dependent DNA ligase domain]	*Escherichia* phage vB_EcoM_JS09 (0.0)	100	0	N
178	102810-102998	+	62	Hypothetical protein	*Shigella* phage SSE1 (9e-37)	100	0	N
179	103054-105141	+	695	Alt-like RNA polymerase ADP-ribosyltransferase [PF03496; ADPrib_exo_Tox; ADP-ribosyltransferase exoenzyme]	*Escherichia* phage ST0 (0.0)	100	0	N
180	105200-105493	+	97	Hypothetical protein [PF17602; DUF5498; Family of unknown function (DUF5498)]	*Escherichia* phage vB_EcoM_JS09 (1e-63)	100	0	N
181	105526-106488	-	320	Tail tube	*Shigella* phage Shf125875 (0.0)	100	0	N
182	106488-107597	-	369	Baseplate tail tube cap [PF11091; T4_tail_cap; Tail-tube assembly protein]	*Escherichia* phage ST0 (0.0)	100	0	N
183	107606-109378	-	590	Tape measure protein	*Escherichia* phage S143_2 (0.0)	100	0	N
184	109375-109845	-	156	Baseplate hub distal subunit [PF11110; Phage_hub_GP28; Baseplate hub distal subunit]	*Shigella* phage Shf125875 (1e-110)	100	0	N
185	109856-111028	-	390	Baseplate hub [PF09096 ; Phage-tail_2 ; Baseplate structural protein, domain 2]	*Escherichia* phage F2 (0.0)	99.4	0	N
186	111025-111777	-	250	Baseplate hub assembly protein [PF12322; T4_baseplate; T4 bacteriophage base plate protein]	*Shigella* phage JK45 (0.0)	99.6	0	N
187	111825-112451	+	208	Baseplate hub subunit [PF12322; T4_baseplate; T4 bacteriophage base plate protein]	*Shigella* phage SSE1 (6e-153)	100	0	N
188	112451-112849	+	132	Putative baseplate wedge subunit [PF04965; GPW_gp25; Baseplate wedge protein gp25]	*Escherichia* phage vB_EcoM_G2469 (6e-90)	99.2	0	N
189	112849-113343	+	164	UvsY-like recombination mediator [PF11056; UvsY; Recombination, repair and ssDNA binding protein UvsY]	*Escherichia* phage RB69 (3e-116)	100	0	N

190	113343-113567	+	74	Hypothetical protein	*Escherichia* phage vB_EcoM_JS09 (3e-46)	100	0	N
191	113600-113767	+	55	Hypothetical protein [PF10886; DUF2685; Protein of unknown function (DUF2685)]	*Escherichia* phage vB_EcoM_JS09 (9e-32)	100	0	N
192	113826-114059	-	77	DNA helicase [PF11637; UvsW-1; UvsW.1 domain]	*Escherichia* phage F2 (6e-45)	98.7	0	N
193	114085-115599	-	504	DNA helicase [PF21241 ; UvsW_N ; DNA helicase UvsW, N-terminal]	*Escherichia* phage ST0 (0.0)	100	0	N
194	115650-116318	+	222	Inhibitor of prohead protease	*Escherichia* phage FP43 (9e-158)	100	0	N
195	116328-117746	+	472	Hypothetical protein [PF00801; PKD; PKD domain]	*Escherichia* phage JN02 (0.0)	94.2	0	N
196	117848-118042	+	64	Hypothetical protein [PF11242; DUF2774; Protein of unknown function (DUF2774)]	*Escherichia* phage RB69 (2e-36)	100	0	N
197	118039-118290	+	83	Hypothetical protein	*Shigella* phage SSE1 (8e-55)	100	0	N
198	118409-119407	+	332	RNA ligase 2 [PF09414; RNA_ligase; RNA ligase]	*Escherichia* phage vB_EcoM-ZQ3 (0.0)	99.1	0	N
199	119438-120721	-	427	Capsid vertex protein [PF07068; Gp23; Major capsid protein Gp23]	*Escherichia* phage vB_EcoM_JS09 (0.0)	100	0	N
200	120823-121092	+	89	Hypothetical protein	*Escherichia* phage vB_EcoM_JS09 (3e-57)	100	0	N
201	121145-122713	-	522	Major capsid protein [PF07068; Gp23; Major capsid protein Gp23]	*Escherichia* phage vB_EcoM_JS09 (0.0)	100	0	N
202	122731-123543	-	270	Head scaffolding protein	*Escherichia* phage vB_EcoM_JS09 (0.0)	100	0	N
203	123577-124224	-	215	Prohead core scaffold and protease [PF03420; Peptidase_S77; Prohead core protein serine protease]	*Shigella* phage SSE1 (3e-154)	100	0	N
204	124224-124649	-	141	Head scaffolding protein	*Escherichia* phage phiC120 (3e-95)	99.2	0	N
205	124649-124885	-	78	Prohead core protein [PF17634; GP67; Gene product 67]	*Escherichia* phage vB_EcoM_NBG1 (1e-42)	98.7	0	N
206	124885-126456	-	523	Portal protein [PF07230; Portal_Gp20; Bacteriophage T4-like portal protein (Gp20)]	*Escherichia* phage vB_EcoM_JS09 (0.0)	100	0	N

207	126541-127032	-	163	Tail protein [PF06841; Phage_T4_gp19; T4-like virus tail tube protein gp19]	*Escherichia* phage RB69 (1e-115)	99.3	0	N
208	127145-129127	-	660	Tail sheath [PF17481 ; Phage_sheath_1N ; Phage tail sheath protein beta-sandwich domain]	*Escherichia* phage vB_EcoM_JS09 (0.0)	100	0	N
209	129158-130993	-	611	Terminase large subunit [PF17289 ; Terminase_6C ; Terminase RNaseH-like domain]	*Escherichia* phage HX01 (0.0)	100	0	N
210	130977-131471	-	164	Terminase small subunit [PF11053; DNA_Packaging; Terminase DNA packaging enzyme]	*Shigella* phage SHSML-52-1 (5e-117)	100	0	N
211	131481-132302	-	273	Putative tail sheath stabilizer and completion protein [PF16724; T4-gp15_tss; T4-like virus Myoviridae tail sheath stabiliser]	*Escherichia* phage vB_EcoM_G2285 (0.0)	100	0	N
212	132355-133119	-	254	Head closure Hc2 [PF11649; T4_neck-protein; Virus neck protein]	*Escherichia* phage vB_EcoM_JS09 (0.0)	99.6	0	N
213	133121-134047	-	308	Head-tail adaptor Ad2	*Escherichia* phage vB_EcoM_JS09 (0.0)	100	0	N
214	134080-135528	-	482	Fibritin neck whisker [PF07921; Fibritin_C; Fibritin C-terminal region]	*Escherichia* phage ST0 (0.0)	100	0	N
215	135528-137111	-	527	Tail collar fiber protein [PF07484 ; Collar ; Phage Tail Collar Domain]	*Escherichia* phage ST0 (0.0)	100	0	N
216	137108-137767	-	219	Baseplate wedge subunit [PF08677; GP11; GP11 baseplate wedge protein]	*Escherichia* phage ST0 (2e-158)	100	0	N
217	137767-139572	-	601	Baseplate wedge subunit and tail pin [PF07880; T4_gp9_10; Bacteriophage T4 gp9/10-like protein]	*Escherichia* phage PNJ-6 (0.0)	100	0	N
218	139572-140444	-	290	Putative baseplate wedge tail fiber connector [PF07880; T4_gp9_10; Bacteriophage T4 gp9/10-like protein]	*Escherichia* phage vB_EcoM_G2285 (0.0)	100	0	N
219	140517-141521	-	334	Baseplate wedge subunit	*Escherichia* phage vB_EcoM-RPN187 (0.0)	100	0	N
220	141514-144612	-	1032	Baseplate wedge protein [PF21428 ; Gp7_helical ; Baseplate wedge protein gp7, helical domain]	*Escherichia* phage 308Ecol101PP (0.0)	100	1	N
221	144609-146582	-	657	Baseplate wedge subunit [PF21515 ; Gp6_2nd ; Baseplate wedge protein gp6, domain II]	*Escherichia* phage vB_EcoM_ZQ3 (0.0)	100	0	N
222	146591-146884	-	97	PAAR motif of membrane proteins	*Escherichia* phage SF (2e-63)	98.9	0	N

223	146887-147360	-	157	Hypothetical protein	*Escherichia* phage vB_EcoM-ZQ3 (2e-109)	99.3	0	N
224	147406-149139	-	577	Baseplate hub subunit and tail lysozyme [PF00959; Phage_lysozyme; Phage lysozyme]	*Escherichia* phage vB_EcoM-RPN187 (0.0)	99.8	0	N
225	149139-149714	-	191	Baseplate wedge subunit [PF11246; Phage_gp53; Base plate wedge protein 53]	*Shigella* phage SHSML-52-1 (1e-137)	100	0	N
226	149776-150225	+	149	Phage head completion protein [PF08722; Tn7_Tnp_TnsA_N; TnsA endonuclease N terminal]	*Escherichia* phage vB_Eco_NicPhage (6e-106)	100	0	N
227	150228-151049	+	273	DNA end protector	*Shigella* phage Shf125875 (0.0)	99.6	0	N
228	151152-151736	+	194	Tail completion and sheath stabilizer protein [PF06841; Phage_T4_gp19; T4-like virus tail tube protein gp19]	*Escherichia* phage vB_EcoM_JS09 (1e-142)	100	0	N
229	151790-152524	+	244	Deoxynucleoside monophosphate kinase [PF21448 ; DNMK ; Deoxynucleotide monophosphate kinase]	*Escherichia* phage APCEc01 (46e-178)	100	0	N
230	152529-152759	+	76	Chaperone for tail fiber formation [PF17594; GP57; Phage Tail fiber assembly helper gene product 57]	*Escherichia* phage PNJ-6 (4e-42)	100	0	N
231	152759-153214	+	151	RNA ligase	*Escherichia* phage vB_EcoM_JS09 (1e-107)	100	0	N
232	153291-153578	+	95	Internal virion protein [PF11634; IPI_T4 ; Nuclease inhibitor from bacteriophage T4]	*Escherichia* phage vB_EcoM_PhAPEC2 (1e-59)	98.9	0	N
233	153649-153834	+	61	Hypothetical protein	*Escherichia* phage AlbertHofmann (5e-33)	100	2	N
234	153836-154198	+	120	Hypothetical protein	*Escherichia* phage PNJ-6 (4e-84)	100	0	N
235	154195-154485	+	96	Hypothetical protein	*Escherichia* phage vB_EcoM_PhAPEC2 (4e-62)	98.9	0	N
236	154490-155005	+	171	Hypothetical protein	*Escherichia* phage PNJ-6 (5e-116)	98.2	0	N
237	155258-155602	+	114	Hypothetical protein	*Shigella* phage Shf125875 (1e-79)	100	0	N
238	155978-156604	+	208	Hypothetical protein	*Escherichia* phage ST0 (4e-149)	99.5	0	N

239	156714-157217	+	167	Hypothetical protein	*Escherichia* phage vB_EcoM-ZQ3 (4e-114)	98.8	0	N
240	157276-157503	+	75	Hypothetical protein [PF05798; Phage_FRD3; Bacteriophage FRD3 protein]	*Shigella* phage SHSML-52-1 (3e-48)	100	0	N
241	157528-157809	+	93	Hypothetical protein	*Escherichia* phage vB_EcoM_SA79RD (2e-58)	100	0	N
242	157881-158474	+	197	Hypothetical protein	*Escherichia* phage SF (8e-135)	99.4	0	N
243	158524-159120	+	198	Hypothetical protein	*Escherichia* coli O157 typing phage 3 (1e-114)	98.9	0	N
244	159463-159822	+	119	Hypothetical protein	*Escherichia* phage vB_EcoM_JS09 (1e-82)	100	2	N
245	159819-160124	+	101	Hypothetical protein	*Escherichia* phage ST0 (1e-66)	100	0	N
246	160134-160406	+	90	Hypothetical protein	*Escherichia* phage vB_EcoM_SQ17 (7e-60)	100	0	N
247	160416-160613	+	65	Hypothetical protein	*Escherichia* phage phiC120 (1e-37)	98.4	0	N
248	160676-160915	+	79	Hypothetical protein	*Shigella* phage SHSML-52-1 (2e-51)	100	0	N
249	160940-161896	+	318	Hypothetical protein	*Escherichia* phage vB_EcoM_JS09 (0.0)	99	0	N
250	161967-162272	+	101	Hypothetical protein	*Shigella* phage phi25-307 (1e-66)	98	2	N
251	162274-162960	+	228	Hypothetical protein	*Escherichia* phage ChristianSchoenbein (5e-166)	96.4	0	N
252	162960-163451	+	163	Membrane protein	*Escherichia* phage vB_EcoM_PhAPEC2 (1e-112)	98.7	2	N
253	163448-163684	+	78	Hypothetical protein	*Escherichia* phage vB_EcoM_TU01 (2e-45)	98.7	0	N
254	163674-164132	+	152	Nudix hydrolase [PF00293; NUDIX; NUDIX domain]	*Escherichia* phage vB_EcoM_JS09 (7e-111)	100	0	N

255	164167-164655	+	162	Endolysin protein e [PF00959; Phage_lysozyme; Phage lysozyme]	*Escherichia* phage ChristianSchoenbein (9e-116)	99.3	0	N
256	164652-164933	+	93	Putative internal head protein	*Shigella* phage vB_SboM_Phaginator (5e-58)	100	0	N
257	164992-165405	+	137	Endonuclease V N-glycosylase UV repair enzyme [PF03013; Pyr_excise; Pyrimidine dimer DNA glycosylase]	*Escherichia* phage HX01 (4e-97)	100	0	N
258	165497-165799	+	100	Internal protein II	*Escherichia* phage ECO07P1 (5e-64)	98	0	N
259	166072-166611	+	179	Hypothetical protein	*Escherichia* phage vB_EcoM_ZQ3 (2e-131)	100	0	N
260	166608-166916	+	102	Hypothetical protein	*Escherichia* phage vB_EcoM_PhAPEC2 (2e-70)	100	0	N
261	166923-167285	+	120	Pyruvate formate-lyase [PF01228; Gly_radical; Glycine radical]	*Shigella* phage SSE1 (4e-82)	100	0	N
262	167285-167509	+	74	Hypothetical protein	*Escherichia* phage F2 (3e-46)	98.6	0	N
263	167499-167765	+	88	Hypothetical protein	*Escherichia* phage F2 (2e-57)	100	0	N
264	167765-167980	+	71	Hypothetical protein	*Escherichia* phage APCEc01 (5e-45)	100	0	N
265	168043-168501	+	152	Endoribonuclease [PF10715; REGB_T4; T4-page Endoribonuclease RegB]	*Escherichia* phage vB_EcoM-RPN187 (3e-107)	99.3	0	N
266	168510-168713	+	68	Gpvs.1 protein	*Escherichia* phage RB69 (7e-42)	100	0	Y
