# Supplementary material for: Characterization of Five Lytic Bacteriophages as New Members of the Genus Mosigvirus, Infecting Multidrug-Resistant Shiga Toxin-Producing Escherichia coli and Their Antibiofilm Activity
Source: Viruses. 2025 Nov 13;17(11):1501. doi: 10.3390/v17111501 (PMC12656860; doi:10.3390/v17111501)
Supplement: Supplementary file 1 [file viruses-17-01501-s001.zip › Table S4.pdf]

Table S4. Features of predicted ORFs and their homology to STEC phage ΦK.

Gene product				Putative function [Conserved domain]	Best match organism (E-value)	Identity (%)	Predicted TMHHM and signal peptide	
ORF No.	Range	Strand	Length (AA)				TMHHM	SignalP
1	1-2214	+	737	Riia Lysis Inhibitor	*Escherichia* phage ST0 (0.0)	100	0	N
2	2224-3171	+	315	Riia Lysis Inhibitor	*Escherichia* phage vB_EcoM_JS09 (0.0)	99.7	0	N
3	3213-3500	+	95	Hypothetical Protein	*Escherichia* phage vB_EcoM_JS09 (2e-62)	100	0	N
4	3517-3993	+	158	Endonuclease IV	*Shigella* phage SSE1 (3e-113)	100	0	N
5	4062-4325	+	87	Hypothetical Protein	*Escherichia* phage RB69 (4e-57)	100	0	N
6	4405-4515	+	36	Hypothetical Protein	*Escherichia* phage vB_EcoM_JS09 (1e-16)	100	1	N
7	4576-4776	+	66	Hypothetical Protein	*Escherichia* phage vB_EcoM_PhAPEC2 (6e-41)	100	0	N
8	4853-5299	+	148	Ndd-like nucleoid disruption protein	*Escherichia* phage vB_EcoM_JS09 (3e-104)	100	0	N
9	5352-5498	+	48	Ac Acridine Resistance Protein	*Escherichia* phage RB69 (9e-24)	100	1	N
10	5498-5638	+	46	Hypothetical Protein	*Escherichia* phage vB_EcoM_PhAPEC2 (1e-20)	100	1	N
11	5643-6968	+	441	DNA Topoisomerase II [PF00521; DNA_topoisoIV; DNA gyrase/topoisomerase IV, subunit A]	*Escherichia* phage ST0 (0.0)	99.5	0	N
12	6965-7069	+	34	Hypothetical Protein	*Escherichia* phage MX01 (2e-12)	100	1	N
13	7178-7810	+	210	Activator Of Middle Period Transcription	*Escherichia* phage APCEc01 (4e-147)	100	0	N
14	7821-8165	+	114	Hypothetical Protein	*Escherichia* phage HX01 (4e-78)	100	0	N

15	8162-8623	+	153	Anti-Restriction Nuclease	*Escherichia* phage HX01 (9e-110)	100	0	N
16	8623-8904	+	93	Hypothetical Protein	*Escherichia* phage HX01 (1e61)	100	0	N
17	9081-9488	+	135	Hypothetical Protein	*Escherichia* phage vB_EcoM-ZQ3 (2e-66)	100	0	N
18	9478-9639	+	53	Hypothetical Protein	*Escherichia* phage HX01 (4e-29)	100	0	N
19	9686-9958	+	90	Anti-sigma factor [PF09010; AsiA; Anti-Sigma Factor A]	*Escherichia* phage AV116 (5e-56)	100	0	N
20	9959-10618	-	219	Holin [PF11031; Phage_holin_T; Bacteriophage T holin]	*Escherichia* phage slur14 (1e-158)	99	1	N
21	10628-11179	-	183	Tail fiber assembly	*Shigella* phage SHSML-52-1 (3e-129)	100	0	N
22	11210-14407	-	1065	Tail Fibers Protein	*Escherichia* phage mogra (0.0)	82	0	N
23	14416-15081	-	221	hinge connector of long tail fiber protein distal connector	*Shigella* phage phi25-307 (2e-155)	97.7	0	N
24	15144-16271	-	375	Long-Tail Fiber Protein prioximal connector	*Escherichia* phage vB_EcoM_JS09 (0.0)	99.4	0	N
25	16280-20155	-	1291	Long tail fiber protein proximal subunit [PF14366; DUF4410; Domain of unknown function (DUF4410)]	*Shigella* phage SHSML-25-1 (0.0)	98.3	0	N
26	20259-21176	+	305	Ribonuclease H	*Escherichia* phage SF (0.0)	100	0	N
27	21184-21453	+	89	Transcriptional regulator [PF11126; Phage_DsbA; Transcriptional regulator DsbA]	*Escherichia* phage RB69 (5e-56)	100	0	N
28	21431-21769	+	112	Late promoter transcriptional regulator	*Escherichia* phage RB69 (1e-74)	100	0	N
29	21781-22419	+	217	DNA Helicase Loader	*Escherichia* phage HX01 (2e-156)	100	0	N
30	22542-23444	+	300	Single-Stranded DNA Binding Protein [PF08804; gp32; gp32 DNA binding protein like]	*Escherichia* phage vB_EcoM_PhAPEC2 (0.0)	100	0	N

31	23558-23962	+	134	Hypothetical Protein	*Escherichia* phage HX01 (2e-92)	100	0	N
32	24027-24275	+	82	DUF5417 domain-containing protein	*Escherichia* phage vB_EcoM_JS09 (3e-52)	98.7	0	N
33	24278-24526	+	82	Hypothetical Protein	*Escherichia* phage vB_EcoM_JS09 (5e-53)	100	0	N
34	24519-25106	+	195	Dihydrofolate Reductase [PF00186; DHFR_1; Dihydrofolate reductase]	*Escherichia* phage vB_EcoM_JS09 (5e-142)	100	0	N
35	25103-25963	+	286	Thymidylate Synthase [PF00303; Thymidylat_synt; Thymidylate synthase]	*Escherichia* phage JN02 (0.0)	100	0	N
36	25965-26219	+	84	Hypothetical Protein	*Escherichia* phage ST0 (2e-53)	100	0	N
37	26307-28562	+	751	NrdA-like aerobic NDP reductase large subunit [PF02867; Ribonuc_red_lgC; Ribonucleotide reductase, barrel domain]	*Escherichia* phage HX01 (0.0)	100	0	N
38	28615-29793	+	392	Ribonucleotide reductase of class Ia beta subunit [PF00268; Ribonuc_red_sm; Ribonucleotide reductase, small chain]	*Escherichia* phage vB_EcoM_JS09 (0.0)	100	0	N
39	29820-30230	+	136	Putative endonuclease [IPR016413Phage_T4_DenA_endoDNaseII ; Bacteriophage T4, DenA, endonuclease II]	*Escherichia* phage vB_EcoM_MM02 (4e-94)	99.2	0	N
40	30287-31411	+	374	RNA ligase tail fiber protein attachment catalyst [IPR012648 ; Rnl1 ; T4 RNA ligase 1]	*Escherichia* phage APCRc01 (0.0)	99.7	0	N
41	31473-31973	+	166	Inhibitor of Host Transcription [PF17527; ALC; Phage ALC protein]	*Shigella* phage JK45 (3e-119)	100	0	N
42	31961-32317	+	118	Hypothetical Protein	*Escherichia* phage vB_EcoM_G2285 (2e-76)	99.1	1	N
43	32314-32604	+	96	hypothetical protein MM02_00234	*Escherichia* phage vB_EcoM_MM02 (4e-65)	98.6	0	Y
44	32601-32819	+	72	Hypothetical Protein	*Escherichia* phage phiC120 (8e-45)	100	0	N
45	32877-33176	+	99	Hypothetical Protein	*Escherichia* phage vB_EcoM_JS09 (8e-64)	100	0	N
46	33176-33367	+	63	Hypothetical Protein	*Escherichia* phage vB_EcoM-172859UKE1 (3e-37)	98.4	0	N

47	33364-34263	+	299	Polynucleotide Kinase [IPR044493 ; PNKP_C_HAD ; Polynucleotide kinase PNKP, C-terminal phosphatase domain]	*Shigella* phage phi25-307 (0.0)	99.3	0	N
48	34263-34454	+	63	Hypothetical Protein	*Escherichia* phage ST0 (3e-37)	100	0	N
49	34444-34659	+	71	Hypothetical Protein	*Escherichia* phage RB69 (2e-44)	100	0	N
50	34667-34942	+	91	Hypothetical Protein	*Escherichia* phage vB_EcoM_G2285 (7e-58)	98.9	0	N
51	35003-35239	+	78	Hypothetical Protein	*Escherichia* hage vB_EcoM_JS09 (4e-49)	100	0	N
52	35226-35351	+	41	Hypothetical Protein	*Shigella* phage SSE1 (2e-19)	100	0	N
53	35360-36352	+	330	phospho-2-dehydro-3-deoxyheptonate aldolase [PF00793; DAHP_synth_1 ; DAHP synthetase I family]	*Escherichia* phage ST0 (0.0)	100	0	N
54	36352-36933	+	193	dCMP deaminase	*Escherichia* phage ST0 (4e-141)	100	0	N
55	36935-37231	+	98	Hypothetical Protein	*Escherichia* phage ST0 (4e-65)	100	0	N
56	37289-37621	+	110	Head assembly chaperone protein [PF00166; Cpn10; Chaperonin 10 Kd subunit]	*Escherichia* phage vB_EcoM-ZQ3 (3e-71)	99	0	N
57	37746-37994	+	82	hypothetical protein RPN187_gp212 [IPR034688 ; Linr3 ; Lysis inhibition accessory protein rIII]	*Escherichia* phage vB_EcoM-RPN187 (1e-51)	98.8	0	N
58	38263-38442	+	59	1,4-alpha-glucan (glycogen) branching enzyme, GH-13-type	*Escherichia phage AV116* (1e-31)	100	0	N
59	38573-38941	+	122	Hypothetical Protein	*Escherichia* phage vB_EcoM_JS09 (1e-84)	100	0	N
60	39016-39381	+	121	Hypothetical Protein	*Escherichia* phage vB_EcoM_JS09 (9e-86)	100	0	N
61	39417-40031	+	204	hypothetical protein FDI17_gp172	*Escherichia* phage ST0 (9e-149)	98	0	N
62	40089-40286	+	65	hypothetical protein KMC05_gp174	*Escherichia* phage F2 (2e-38)	98.4	0	N

63	40276-40488	+	70	Hypothetical Protein	*Escherichia* phage 55 (7e-42)	98.5	0	N
64	40559-40939	+	152	Hypothetical Protein	*Shigella* phage Shf125875 (3e-105)	98.6	0	N
65	40936-41751	+	271	Hypothetical Protein	*Shigella* phage SSE1 (0.0)	100	0	N
66	41761-42030	+	89	Hypothetical Protein	*Escherichia* phage vB_EcoM_JS09 (0.0)	100	0	N
67	42027-43520	+	497	ATP-dependent DNA ligase	*Escherichia* phage vB_EcoM_JS09 (0.0)	100	0	N
68	43520-43708	+	62	Hypothetical Protein	*Shigella* phage SSE1 (1e-37)	100	0	N
69	43764-45851	+	695	RNA polymerase ADP-ribosylase [PF03496; ADPrib_exo_Tox; ADP-ribosyltransferase exoenzyme]	*Shigella* phage SSE1 (0.0)	99.8	0	N
70	45910-46203	+	97	Hypothetical Protein	*Escherichia* phage vB_EcoM_JS09 (8e-64)	100	0	N
71	46236-47198	-	320	tail tube	*Escherichia* phage vB_EcoM_JS09 (0.0)	99.6	0	N
72	47198-48307	-	369	Baseplate tail tube cap	*Escherichia* phage vB_EcoM_JS09 (0.0)	100	0	N
73	48316-50088	-	590	Baseplate hub subunit and tail length	*Escherichia* phage HX01 (0.0)	99.4	0	N
74	50085-50555	-	156	Baseplate Hub Subunit, Distal	*Shigella* phage Shf125875 (9e-111)	100	0	N
75	50566-51738	-	390	Putative baseplate hub subunit [PF09097; Phage-tail_1; Baseplate structural protein, domain 1]	*Escherichia* phage vB_EcoM_WFbE185 (0.0)	99.7	0	N
76	51735-52487	+	250	Baseplate Hub Assembly Protein	*Shigella* phage JK45 (0.0)	99.6	0	N
77	52535-53161	+	208	Baseplate hub assembly chaperone	*Escherichia* phage AV111 (4e-153)	99.5	0	N
78	53161-53559	+	132	Baseplate Wedge Subunit	*Escherichia* phage vB_EcoM_G2469 (6e-90)	99.2	0	N

79	53583-54053	+	164	UvsY-like recombination mediator	*Escherichia* phage RB69 (3e-116)	100	0	N
80	54053-54277	+	74	Hypothetical Protein	*Escherichia* phage ST0 (2e-46)	100	0	N
81	54310-54477	+	55	Hypothetical Protein	*Escherichia* phage vB_EcoM_JS09 (5e-32)	100	0	N
82	54535-54768	-	77	DNA helicase	*Escherichia* phage F2 (6e-45)	100	0	N
83	54794-56308	-	504	DNA helicase [PF04851; ResIII; Type III restriction enzyme, res subunit]	*Escherichia* phage ST0 (0.0)	100	0	N
84	56359-57027	+	222	Inhibitor of Prohead Protease	*Escherichia* phage FP43 (9e-158)	100	0	N
85	57037-58455	+	472	Hypothetical protein [PF00801; PKD; PKD domain]	*Escherichia* phage JN02 (0.0)	93.4	0	N
86	58555-58749	+	64	Duf2774 Domain-Containing Protein [PF11242; DUF2774; Protein of unknown function (DUF2774)]	*Escherichia* phage F2 (3e-36)	98.4	0	N
87	58746-58997	+	83	Hypothetical Protein	*Escherichia* phage F2 (4e-55)	100	0	N
88	59117-60115	+	332	RNA Ligase 2 [IPR044263 ; Rnl2_vir ; RNA Ligase 2, viral]	*Escherichia* phage p000y (0.0)	99.4	0	N
89	60146-61429	-	427	Capsid Vertex [PF07068; Gp23; Major capsid protein Gp23]	*Escherichia* phage moskry (0.0)	100	0	N
90	61531-61800	+	89	Hypothetical Protein	*Escherichia* phage vB_EcoM_PhAPEC2 (7e-58)	100	0	N
91	61853-63421	-	522	Major Capsid Protein [PF07068; Gp23; Major capsid protein Gp23]	*Escherichia* phage phiC120 (0.0)	100	0	N
92	63439-64251	-	270	Head scaffolding protein [PF04344; CheZ; Chemotaxis phosphatase, CheZ]	*Escherichia* phage vB_EcoM_JS09 (0.0)	100	0	N
93	64285-64932	-	215	Prohead Core Scaffold And Protease	*Shigella* phage SSE1 (3e-154)	100	0	N
94	64932-65357	-	141	Phage capsid and scaffold	*Escherichia* phage vB_Eco_NicPhage (6e-95)	99.2	0	N

95	65357-65587	-	76	Prohead	*Escherichia* coli O157 typing phage 3 (5e-42)	100	0	N
96	65587-67158	-	523	Portal Protein	*Escherichia* phage vB_EcoM_JS09 (0.0)	100	0	N
97	67243-67734	-	163	Tail protein [PF06841; Phage_T4_gp19; T4-like virus tail tube protein gp19]	*Escherichia* phage RB69 (5e-116)	100	0	N
98	67847-69829	-	660	Tail Sheath Monomer	*Escherichia* phage mogra (0.0)	100	0	N
99	69860-71695	-	611	Terminase Large Subunit [PF03237; Terminase_6N; Terminase large subunit, T4likevirus-type, N-terminal]	*Shigella* phage SHSML-52-1 (0.0)	99.6	0	N
100	71679-72173	-	164	Terminase small subunit	*Shigella* phage SHSML-52-1 (5e-117)	100	0	N
101	72183-72959	-	273	Tail Sheath Stabilizer And Completion Protein	*Escherichia* phage vB_EcoM_PhAPEC2 (0.0)	100	0	N
102	73057-73821	-	254	Head closure Hc2	*Escherichia* phage vB_EcoM_JS09 (0.0)	99.6	0	N
103	73823-74749	-	308	Neck Protein	*Escherichia* phage vB_EcoM_JS09 (0.0)	100	0	N
104	74782-76230	-	482	Fibritin neck whisker	*Escherichia* phage vB_EcoM_JS09 (0.0)	99.5	0	N
105	76230-77813	-	527	Short tail fibers Protein [PF14928; S_tail_recep_bd; Short tail fibre protein receptor-binding domain]	Shigella phage SSE1 (0.0)	99.8	0	N
106	77810-78469	-	219	Baseplate Wedge Subunit And Tail Pin	*Escherichia* phage vB_EcoM_JS09 (1e-158)	100	0	N
107	78469-80274	-	601	Putative Baseplate Wedge Subunit And Tail Pin [PF07880; T4_gp9_10; Bacteriophage T4 gp9/10-like protein]	*Escherichia* phage vB_EcoM_WFbE185 (0.0)	99.8	0	N
108	80274-81146	-	290	Baseplate Protein [PF07880; T4_gp9_10; Bacteriophage T4 gp9/10-like protein]	*Escherichia* phage S143_2 (0.0)	100	0	N
109	81219-82223	-	334	Baseplate Wedge Subunit	*Escherichia* phage vB_Eco_F31 (0.0)	100	0	N
110	82216-85269	-	1032	Baseplate Wedge Protein [IPR034697 ; Baseplate wedge protein gp7]	*Escherichia* phage HX01 (0.0)	99.3	1	N

111	85311-87284	-	657	Baseplate Wedge initiator [IPR034698 ; GP6_T4 ; Baseplate wedge protein gp6]	*Escherichia coli O157 typing phage 3* (0.0)	99.8	0	N
112	87293-87586	-	97	Phospholipase	*Escherichia* phage AV110(6e-64)	100	0	N
113	87589-88062	-	157	Putative 18.5 Kda Protein	*Escherichia* phage S143_2 (2e-111)	100	0	N
114	88108-89841	-	577	Baseplate hub subunit and tail lysozyme [PF00959 ; Phage_lysozyme ; Phage lysozyme]	*Escherichia* phage vB_EcoM-RPN187 (0.0)	99.8	0	N
115	89841-90416	-	191	Baseplate Wedge Subunit	*Escherichia* phage vB_EcoM_JS09 (7e-138)	100	0	N
116	90478-90927	+	149	Head closure [PF08722; Tn7_Tnp_TnsA_N; TnsA endonuclease N terminal]	*Escherichia* phage F2 (5e-106)	100	0	N
117	90930-91751	+	273	DNA End Protector Protein	*Shigella* phage Shf125875 (0.0)	100	0	N
118	91854-92438	+	194	Tail tube protein [PF06841; Phage_T4_gp19; T4-like virus tail tube protein gp19]	*Escherichia* coli O157 typing phage 3 (3e-142)	100	0	N
119	92492-93226	+	244	Deoxynucleoside Monophosphate Kinase	*Escherichia* phage APCEc01 (6e-178)	100	0	N
120	93231-93461	+	76	Tail fiber assembly protein	*Escherichia* phage AV117 (4e-42)	100	0	N
121	93461-93916	+	151	RNA ligase	*Escherichia* phage vB_EcoM_JS09 (2e-107)	99.3	0	N
122	93994-94281	+	95	Internal virion protein	*Escherichia* coli O157 typing phage 3 (1e-59)	98.9	0	N
123	94353-94538	+	61	Hypothetical Protein	*Escherichia* phage RB69 (3e-33)	100	2	N
124	94540-94902	+	120	Hypothetical Protein	*Escherichia* phage vB_EcoM_JS09 (2e-85)	100	0	N
125	94899-95189	+	96	Hypothetical Protein	*Escherichia* phage vB_EcoM_JS09 (6e-64)	100	0	N
126	95194-95709	+	171	Hypothetical Protein	*Escherichia* phage vB_EcoM_JS09 (5e-120)	100	0	N

127	95962-96306	+	114	Hypothetical Protein	*Escherichia* phage vB_EcoM_JS09 (2e-79)	100	0	N
128	96683-97309	+	208	Hypothetical Protein	*Escherichia* phage vB_EcoM_JS09 (4e-150)	100	0	N
129	97419-97922	+	167	Hypothetical Protein	*Escherichia* phage p000y (2e-115)	99.4	0	N
130	97982-98146	+	54	Hypothetical Protein	*Escherichia* phage RB69 (8e-31)	100	0	N
131	98193-98420	+	75	Hypothetical Protein	*Escherichia* phage RB69 (4e-35)	98.3	0	N
132	98491-99084	+	197	Hypothetical Protein	*Escherichia* phage ST0 (1e-135)	100	0	N
133	99134-99730	+	198	Hypothetical Protein	*Shigella* phage SSE1 (1e-145)	100	0	N
134	99915-100094	+	124	Hypothetical Protein	*Escherichia* phage S134_2 (2e-86)	98.3	2	N
135	100073-100315	+	80	Putative membrane protein	*Shigella* phage SSE1 (8e-50)	98.7	2	N
136	100427-100732	+	101	Hypothetical Protein	*Escherichia* phage vB_EcoM_JS09 (2e-66)	99	0	N
137	100742-101014	+	90	Hypothetical Protein	*Escherichia* phage SF (5e-60)	100	0	N
138	101024-101221	+	65	Hypothetical Protein	*Escherichia* phage RB69 (6e-37)	96.9	0	N
139	101284-101523	+	79	Hypothetical Protein	*Escherichia* phage moskry (5e-51)	97.5	0	N
140	101548-102504	+	318	Hypothetical Protein	*Escherichia* phage PTK (0.0)	99.1	0	N
141	102575-102880	+	101	Hypothetical Protein	*Escherichia* phage p000v (1e-68)	100	2	N
142	102882-103568	+	228	Hypothetical Protein	*Escherichia* phage p000v (8e-171)	100	0	N

143	103568-104059	+	163	Hypothetical Protein	*Escherichia* phage Av-05 (2e-112)	98.8	2	N
144	104056-104292	+	78	Hypothetical Protein	*Escherichia* phage vB_EcoM_PhAPEC2 (4e-46)	98.7	0	N
145	104285-104740	+	151	Nudix hydrolase	*Escherichia* phage RB69 (9e-109)	99.3	0	N
146	104775-105263	+	162	Endolysin protein e [PF00959; Phage_lysozyme; Phage lysozyme]	*Escherichia* phage ChristianSchoenbein (4e-116)	100	0	N
147	105260-105541	+	93	Putative internal head protein	*Shigella* phage vB_SboM_Phaginator (5e-58)	100	0	N
148	105600-106013	+	137	Endonuclease V, N-Glycosylase Uv Repair Enzyme	*Escherichia* phage vB_EcoM_PhAPEC2 (4e-97)	100	0	N
149	106026-106136	+	36	Hypothetical Protein	*Escherichia* phage vB_EcoM_JS09 (3e-16)	100	0	N
150	106200-106514	+	104	Hypothetical Protein	*Escherichia* phage vB_EcoM_JS09 (3e-70)	100	0	N
151	106541-106843	+	100	Hypothetical Protein	*Escherichia* phage AV117 (4e-64)	99	0	N
152	106901-106999	+	32	Endonuclease V N-glycosylase UV repair enzyme	*Escherichia* phage vB_EcoM_JS09 (2e-11)	100	0	N
153	107173-107712	+	179	Hypothetical Protein	*Escherichia* phage vB_EcoM-172859UKE1 (1e-131)	100	0	Y
154	107709-108017	+	102	Hypothetical Protein	*Shigella* phage SHSML-52-1 (1e-70)	100	0	N
155	108024-108386	+	120	Pyruvate formate-lyase [PF01228; Gly_radical; Glycine radical]	*Shigella* phage SSE1 (3e-81)	99..1	0	N
156	108386-108610	+	74	Hypothetical Protein	*Escherichia* phage vB_EcoM_G2285 (8e-46)	98.6	0	N
157	108600-108845	+	81	Hypothetical Protein	*Escherichia* phage vB_EcoM-172859UKE1 (6e-51)	98.7	0	N
158	108845-109144	+	99	Hypothetical Protein	*Shigella* phage Shf125875 (8e-67)	100	0	N

159	109201-109659	+	152	Endoribonuclease [PF10715; REGB_T4; T4-page Endoribonuclease RegB]	*Escherichia* phage RB69 (2e-107)	99.3	0	N
160	109668-110210	+	180	Hypothetical Protein	*Shigella* phage SSE1 (4e-128)	100	0	Y
161	110207-110554	+	115	Vs Valyl-tRNA Synthetase Modifier	*Shigella* phage Shf125875 (3e-78)	100	0	Y
162	110547-111014	+	155	Phosphatase [PF01661; Macro; Macro domain]	*Escherichia* phage F2 (8e-110)	99.3	0	N
163	111011-111223	+	70	Hypothetical Protein	*Shigella* phage SHSML-52-1 (5e-45)	100	0	N
164	111220-111405	+	61	Hypothetical Protein	*Shigella* phage SSE1 (1e-34)	100	0	N
165	111402-111599	+	65	Hypothetical Protein	*Shigella* phage SSE1 (5e-37)	100	0	N
166	111592-112173	+	193	Thymidine Kinase [PF00265; TK; Thymidine kinase]	*Escherichia coli* O157 typing phage 3 (5e-139)	98.4	0	N
167	112201-112413	+	70	Hypothetical Protein	*Escherichia* phage vB_EcoM_JS09 (8e-42)	100	0	N
168	112426-112728	+	100	Lysis Inhibition Regulator	*Escherichia* phage APCEc01 (1e-68)	100	1	Y
169	112830-113009	+	59	Hypothetical Protein	*Escherichia* phage phiE142 (3e-35)	100	0	N
170	113017-113226	+	69	Hypothetical Protein	*Escherichia* phage mogra (9e-43)	98.5	0	N
171	113272-113394	+	40	Hypothetical Protein	*Escherichia* phage HX01 (4e-18)	100	1	N
172	113391-113570	+	59	Hypothetical Protein	*Shigella* phage SHSML-52-1 (9e-33)	98.3	0	N
173	113572-114105	+	177	Hypothetical Protein	*Escherichia* phage vB_EcoM_WFL6982 (2e-125)	98.8	0	N
174	114115-114588	+	157	Hypothetical Protein	*Escherichia* phage PNJ-6 (5e-109)	99.3	2	N

175	114588-115574	+	328	Thioredoxin	*Escherichia* phage vB_EcoM-ZQ3 (0.0)	99.3	0	N
176	115606-115872	+	88	Hypothetical Protein	*Escherichia* phage vB_EcoM_G2285 (3e-57)	100	1	Y
177	115990-116970	+	326	Hypothetical Protein [IPR003593; AAA+_ATPase ; AAA+ ATPase domain]	*Escherichia* phage F2 (0.0)	100	0	N
178	117109-117396	+	95	Thioredoxin	*Escherichia* phage APCEc01 (8e-61)	98.9	0	N
179	117455-117982	+	175	Hypothetical Protein	*Escherichia* phage vB_EcoM_JS09 (8e-123)	100	0	N
180	118045-119040	+	331	Hypothetical Protein	*Escherichia* phage ST0 (0.0)	100	0	N
181	119096-120031	+	311	Thioredoxin	*Escherichia* phage mogra (0.0)	96.1	0	N
182	120094-121038	+	314	Thioredoxin	*Escherichia* phage moha (0.0)	97.8	0	N
183	121038-121343	+	101	Hypothetical Protein	*Escherichia* phage vB_EcoM_JS09 (2e-69)	100	0	N
184	121343-121756	+	137	Hypothetical Protein	*Escherichia* phage vB_EcoM_NBG1 (5e-95)	100	2	N
185	121749-122012	+	87	Thioredoxin	*Escherichia* phage F2 (1e-57)	100	0	N
186	122009-122365	+	118	Hypothetical Protein	*Shigella* phage Shf125875 (1e-79)	100	0	N
187	122494-122664	+	56	Hypothetical Protein	*Escherichia* phage vB_EcoM_JS09 (4e-33)	100	0	N
188	122666-123079	+	137	Protease Inhibitor	*Escherichia* phage vB_EcoM_JS09 (2e-94)	99.3	0	N
189	123089-123268	+	59	Hypothetical Protein	*Escherichia* phage vB_EcoM_JS09 (2e-35)	98.3	0	N
190	123308-123781	+	157	Recombination Endonuclease Vii	*Escherichia* phage APCEc01 (3e-113e)	100	0	N

191	123778-125595	+	605	Putative Anaerobic Ntp Reductase Large Subunit [PF13597; NRDD; Anaerobic ribonucleoside-triphosphate reductase]	*Escherichia* phage SF (0.0)	99.2	0	N
192	125592-126062	+	156	Anaerobic Ntp Reductase Small Subunit	*Escherichia* phage vB_EcoM-ZQ3 (3e-112)	100	0	N
193	126173-126388	+	71	Hypothetical Protein	*Escherichia* phage HX01 (2e-41)	100	1	N
194	126439-126708	+	105	Hypothetical Protein	*Escherichia* phage vB_EcoM_G2469 (3e-70)	99.1	0	N
195	126674-126997	+	107	Putative Glutaredoxin	*Escherichia* phage vB_EcoM_PhAPEC2 (2e-70)	99.1	0	N
196	127164-127346	+	60	Hypothetical Protein	*Escherichia* phage HP3 (2e-35)	98.3	0	N
197	127343-127591	+	82	Hypothetical Protein	*Shigella* phage phi25-307 (4e-50)	97.6	0	N
198	127599-127892	+	97	Hypothetical Protein	*Escherichia* phage vB_EcoM_JS09 (5e-63)	100	0	N
199	127870-128034	+	44	Hypothetical Protein	*Escherichia* phage HX01 (3e-23)	100	0	N
200	128031-128231	+	66	Hypothetical Protein	*Escherichia* phage vB_EcoM_JS09 (1e-41)	100	0	N
201	128295-128534	+	79	Hypothetical Protein	*Escherichia* phage vB_EcoM_JS09 (6e-50)	98.7	0	N
202	128603-128935	+	133	Hypothetical Protein	*Escherichia* phage F2 (3e-82)	100	0	N
203	128932-129159	+	75	Hypothetical Protein	*Escherichia* phage ST0 (3e-44)	98.7	0	N
204	129156-129425	+	89	Hypothetical Protein	*Escherichia* phage HX01 (9e-59)	100	0	N
205	129498-130055	+	185	RNA Polymerase Sigma Factor [IPR046386; T4_sigma-like_factor ; RNA polymerase sigma-like factor]	*Escherichia* phage vB_EcoM_JS09 (9e-135)	100	0	N
206	130045-130254	+	69	Hypothetical Protein	*Escherichia* phage APCEc01 (3e-42)	100	0	N

207	130256-130579	+	107	Hypothetical Protein	*Escherichia* phage HX01 (5e-69)	100	0	N
208	130800-130973	+	57	Hypothetical Protein	*Escherichia* phage APCEc01 (1e-33)	100	0	N
209	131043-132062	+	339	Recombination Endonuclease Subunit [PF00149; Metallophos; Calcineurin-like phosphoesterase]	*Escherichia* phage ST0 (0.0)	99.7	0	N
210	132059-132316	+	85	Hypothetical Protein	*Escherichia* phage vB_EcoM_PhAPEC2 (5e-55)	100	0	N
211	132303-133163	+	286	Mobd-Like Homing Endonuclease	*Escherichia* phage F2 (0.0)	99.3	0	N
212	133150-134838	+	562	Putative Endonuclease Subunit [PF13476; AAA_23; AAA domain]	*Escherichia* phage vB_EcoM_WFL698 (0.0)	99.6	0	N
213	134893-135081	+	62	Hypothetical Protein	*Escherichia* phage RB69 (4e-38)	100	0	N
214	135094-135510	+	138	Rpba RNA Polymerase Binding Protein	*Escherichia* phage RB69 (1e-98)	100	0	N
215	135551-136237	+	228	Sliding Clamp [IPR046388 ; T4_Clamp_Loader_L ; Sliding-clamp-loader large subunit]	*Escherichia* phage RB69 (6e-165)	100	0	N
216	136313-137275	+	320	Clamp Loader, Small Subunit [PF00004; AAA; ATPase family associated with various cellular activities (AAA)]	*Escherichia* phage RB69 (0.0)	100	0	N
217	137277-137840	+	187	Clamp Loader Small Subunit [PF16790; Phage_clamp_A; Bacteriophage clamp loader A subunit]	*Escherichia* phage RB69 (3e-134)	100	0	N
218	137843-138211	+	122	Translation Repressor Protein [PF01818; Translat_reg; Bacteriophage translational regulator]	*Escherichia* phage RB69 (3e-84)	100	0	N
219	138293-141004	+	903	DNA Polymerase [IPR034749 ; DPOL_T4; DNA-directed DNA polymerase T4 type]	*Escherichia* phage RB69 (0.0)	100	0	N
220	141045-141680	+	211	Arabinose 5-Phosphate Isomerase [PF01380; SIS; SIS domain]	*Shigella* phage phi25-307 (4e-153)	99.5	0	N
221	141677-141820	+	47	Hypothetical Protein	*Escherichia* phage F2 (1e-23)	100	1	N
222	141862-143550	+	562	Hypothetical Protein [PF00483; NTP_transferase; Nucleotidyl transferase]	*Escherichia* phage vB_EcoM-ZQ3 (0.0)	99.6	0	N

223	143550-143936	+	128	Hypothetical Protein	*Escherichia* phage vB_EcoM_JS09 (9e-91)	100	0	N
224	143993-145153	+	386	Putative Protease	Enterobacteria phage ATK48 (0.0)	100	0	N
225	145150-145272	+	40	Hypothetical Protein	*Escherichia* phage PHB12 (4e-19)	100	0	N
226	145433-146149	+	238	Putative Thymidylate Synthase [PF00303; Thymidylat_synt; Thymidylate synthase]	*Escherichia* phage PHB12 (2e-178)	98.7	0	N
227	146149-147048	+	299	Hypothetical Protein	*Escherichia* phage APCEc01 (0.0)	100	0	N
228	147050-147598	+	182	Hypothetical Protein	*Escherichia* phage SF (2e-131)	100	0	N
229	147698-148870	+	390	Uvsx Reca-Like Recombination Protein [PF21134; T4_UVSX_C; recA bacterial DNA recombination protein]	*Escherichia* phage RB69 (0.0)	100	0	N
230	148863-149204	+	113	Head Vertex Assembly Initiator	*Escherichia* phage vB_EcoM-ZQ3 (1e-76)	100	0	N
231	149214-150656	+	480	Helicase [PF03796; DNAB_C; DNAB-like helicase C terminal domain]	*Escherichia* phage ST0 (0.0)	100	0	N
232	150745-151119	+	124	Hypothetical Protein	*Escherichia* phage RB69 (5e-86)	99.2	0	N
233	151175-151492	+	105	Hypothetical Protein	*Escherichia* phage RB69 (4e-73)	100	0	N
234	151489-151680	+	63	Discriminator Of Mrna Degradation	*Escherichia* phage vB_EcoM_PhAPEC2 (1e-35)	100	0	N
235	151682-151894	+	70	Hypothetical Protein	*Escherichia* phage RB69 (4e-44)	100	0	N
236	151955-152323	+	122	Immunity Protein	*Escherichia* phage moha (4e-85)	98.4	0	Y
237	152385-152633	+	82	Imm Immunity To Superinfection Membrane Protein	*Escherichia* phage RB69 (1e-48)	98.8	2	N
238	152697-152990	+	97	Sp Spackle Periplasmic Protein [IPR046391; SPACKLE_T4 ; Protein spackle]	*Escherichia* phage RB69 (1e-65)	99.0	0	Y

239	152992-153642	+	216	Hypothetical Protein	*Escherichia* phage PTK (1e-158)	100	0	N
240	153644-153841	+	65	Hypothetical Protein	*Escherichia* phage vB_EcoM_JS09 (1e-39)	100	0	N
241	153861-154328	+	155	Hypothetical Protein	*Escherichia* phage vB_EcoM_JS09 (5e-110)	100	0	N
242	154368-155390	+	340	DNA Primase Subunit [IPR046392 ; PRIMASE_T4 ; DNA primase, bacteriophage T4]	*Escherichia* phage JN02 (0.0)	99.7	0	N
243	155387-155584	-	65	Hypothetical Protein	*Escherichia* phage ST0 (2e-35)	100	1	N
244	155674-156195	+	173	Dctp Pyrophosphatase	*Escherichia* phage vB_EcoM_JS09 (1e-125)	100	0	N
245	156241-156477	+	78	Small Outer Capsid Protein [PF16855; Soc; Small outer capsid protein]	*Escherichia* phage vB_EcoM_JS09 (2e-50)	100	0	N
246	156772-157008	+	78	Hypothetical Protein	*Escherichia* phage S143_2 (5e-46)	100	0	N
247	157005-157184	+	59	Hypothetical Protein	*Escherichia* phage vB_EcoM_JS09 (1e-33)	100	0	N
248	157184-157543	+	119	Transcription Modulator Under Heat Shock	*Escherichia* phage vB_EcoM_PhAPEC2 (2e-76)	96.6	0	N
249	157649-157828	+	59	Hypothetical Protein	*Escherichia* phage vB_EcoM_JS09 (3e-34)	100	0	N
250	157825-157989	+	54	Hypothetical Protein	*Escherichia* phage RB69 (4e-28)	100	0	N
251	158045-158626	+	193	Adp-Ribosylase [IPR043662 ; ModB-like ; NAD-protein ADP-ribosyltransferase ModB-like]	*Escherichia* phage vB_EcoM_JS09 (1e-141)	99.5	0	N
252	158684-159292	+	202	RNA Polymerase Adp-Ribosylase [IPR043662 ; ModB-like ; NAD-protein ADP-ribosyltransferase ModB-like]	*Escherichia* phage vB_EcoM_NBG1 (8e-149)	99.5	0	N
253	159445-160191	+	248	Putative Srd Anti-Sigma Factor	*Escherichia* phage RB69 (7e-179)	100	0	N
254	160194-160505	+	103	Hypothetical Protein	*Escherichia* phage F2 (6e-69)	100	0	N

255	160502-161815	+	437	DNA Helicase [PF18343; SH3_14; Dda helicase SH3 domain]	*Escherichia* phage ST0 (0.0)	100	0	N
256	161825-162502	+	225	Exonuclease [PF16473; DUF5051; 3' exoribonuclease, RNase T-like]	*Escherichia* phage SF (8e-166)	99.6	0	N
257	162568-163062	+	164	Hypothetical Protein	*Escherichia* phage vB_EcoM_JS09 (5e-116)	98.2	0	N
258	163124-163579	+	151	Transcriptional Regulator	*Escherichia* phage vB_EcoM-ZQ3 (2e-107)	99.3	0	N
259	163589-164008	+	139	Transcriptional Regulator [PF17613; motB; Modifier of transcription]	*Escherichia* phage vB_EcoM-ZQ3 (8e-96)	99.3	0	N
260	164068-164592	+	174	Transcriptional Regulator	*Escherichia* phage moha (2e-126)	99.4	0	N
261	164650-164877	+	75	Protein Cef	*Escherichia* phage S143_2 (5e-47)	100	0	N
262	164877-165287	+	136	Putative RNA Metabolism Moderator	*Escherichia* phage vB_EcoM-ZQ3 (6e-97)	100	0	N
263	165290-165469	+	59	Gp39.2 Conserved Hypothetical Protein	*Escherichia* phage RB69 (5e-36)	100	0	N
264	165472-165897	+	141	Hypothetical Protein	*Escherichia* phage ST0 (8e-96)	100	0	N
265	165961-167778	+	605	DNA Gyrase Subunit B [PF00204; DNA_gyraseB; DNA gyrase B]	*Escherichia* phage moskry (0.0)	99.7	0	N
266	167821-168921	+	366	Hypothetical Protein	*Escherichia* phage F2 (0.0)	100	0	N
267	169014-169214	+	66	Hypothetical Protein	*Escherichia* phage HX01 (5e-37)	100	0	N
