# Supplementary material for: Characterization of Five Lytic Bacteriophages as New Members of the Genus Mosigvirus, Infecting Multidrug-Resistant Shiga Toxin-Producing Escherichia coli and Their Antibiofilm Activity
Source: Viruses. 2025 Nov 13;17(11):1501. doi: 10.3390/v17111501 (PMC12656860; doi:10.3390/v17111501)
Supplement: Supplementary file 1 [file viruses-17-01501-s001.zip › Table S5.pdf]

**Table S5**. Features of predicted ORFs and their homology to STEC phage ΦL.

| Gene product | | | | Putative function [Conserved domain] | Best match organism (E-value) | Identity (%) | Predicted TMHHM and signal peptide | |
|---|---|---|---|---|---|---|---|---|
| ORF No. | Range | Strand | Length (AA) | | | | TMHHM | SignalP |
| 1 | 1-2214 | + | 737 | rIIA lysis inhibitor | *Shigella* phage SSE1 (0.0) | 99.8 | 0 | N |
| 2 | 2224-3171 | + | 315 | RIIB lysis inhibitor | *Escherichia* phage p000v (4e-62) | 98.9 | 0 | N |
| 3 | 3213-3500 | + | 95 | Hypothetical protein | *Escherichia* phage RB69 (3e-62) | 100 | 0 | N |
| 4 | 3517-3993 | + | 158 | DenB-like DNA endonuclease IV | *Escherichia* phage phiE142 (1e-112) | 100 | 0 | N |
| 5 | 4062-4325 | + | 87 | Hypothetical protein | *Escherichia* phage vB_EcoM_G53 (1e-56) | 98.8 | 0 | N |
| 6 | 4405-4515 | + | 36 | Hypothetical protein | *Shigella* phage ESh27 (1e-15) | 97.2 | 1 | Y |
| 7 | 4576-4776 | + | 66 | Hypothetical protein | *Escherichia* phage vB_EcoM_JS09 (5e-41) | 100 | 0 | N |
| 8 | 4854-5300 | + | 148 | Nucleoid disruption protein | *Escherichia* phage vB_EcoM_JS09 (2e-103) | 99.3 | 0 | N |
| 9 | 5353-5499 | + | 48 | Hypothetical protein | *Escherichia* coli O157 typing phage 3 (6e-23) | 97.9 | 2 | N |
| 10 | 5644-6969 | + | 441 | DNA topoisomerase II [PF00521; DNA_topoisoIV; DNA gyrase/topoisomerase IV, subunit A] | *Shigella* phage phi25-307 (0.0) | 99.7 | 0 | N |
| 11 | 7158-7373 | + | 71 | Hypothetical protein | *Escherichia* phage vB_EcoM_PhAPEC2 (8e-41) | 97.1 | 0 | N |
| 12 | 7477-8109 | + | 210 | MotA activator of middle period transcription | *Escherichia* phage RB69 (5e-147) | 99.5 | 0 | N |
| 13 | 8120-8449 | + | 109 | Hypothetical protein | *Escherichia* phage p000v (2e-74) | 100 | 0 | N |
| 14 | 8446-8907 | + | 153 | Hypothetical protein | *Escherichia* phage vB_EcoM_JS09 (1e-109) | 100 | 0 | N |

| | | | | | | | | |
|---|---|---|---|---|---|---|---|---|
| 15 | 8907-9188 | + | 93 | Anti-restriction nuclease | *Shigella* phage SHSML-52-1 (6e-61) | 98.9 | 0 | N |
| 16 | 9365-9484 | + | 39 | Hypothetical protein | *Escherichia* phage vB_EcoM_JS09 (2e-17) | 100 | 0 | N |
| 17 | 9474-9773 | + | 99 | Hypothetical protein | *Escherichia* phage vB_EcoM_EP32a (6e-66) | 100 | 0 | N |
| 18 | 9763-9924 | + | 53 | Hypothetical protein | *Escherichia* phage OLB35 (4e-28) | 98.1 | 0 | N |
| 19 | 9971-10243 | + | 90 | anti-sigma factor [PF09010; AsiA; Anti-Sigma Factor A] | *Escherichia* phage AV116 (5e-56) | 100 | 0 | N |
| 20 | 10244-10903 | - | 219 | Putative holin [PF11031; Phage_holin_T; Bacteriophage T holin] | *Escherichia* phage vB_EcoM_G53 (4e-159) | 99.5 | 1 | N |
| 21 | 10913-11464 | - | 183 | Tail fiber assembly | Enterobacteria phage Aplg8 (4e-129) | 99.4 | 0 | N |
| 22 | 11495-14722 | - | 1075 | Long tail fiber distal subunit | *Escherichia* phage vB_EcoM_SA79RD (0.0) | 86.5 | 0 | N |
| 23 | 14731-15396 | - | 221 | Hinge connector of long tail fiber protein distal connector | *Escherichia* phage UGJNEcP1 (5e-158) | 99.5 | 0 | N |
| 24 | 15459-16586 | - | 375 | Long tail fiber protein proximal connector | *Escherichia* phage vB_EcoM_JS09 (0.0) | 99.7 | 0 | N |
| 25 | 16595-20470 | - | 1291 | Long tail fiber proximal subunit | *Escherichia* phage moskry (0.0) | 99.4 | 0 | N |
| 26 | 20574-21491 | + | 305 | Ribonuclease H [PF09293; RNaseH_C; T4 RNase H, C terminal] | *Shigella* phage SHSML-52-1 (0.0) | 100 | 0 | N |
| 27 | 21499-21768 | + | 89 | Transcriptional regulator | *Escherichia* phage RB69 (5e-56) | 100 | 0 | N |
| 28 | 21746-22084 | + | 112 | Late promoter transcription accessory protein | *Escherichia* phage PaulHMueller (3e-74) | 99.1 | 0 | N |
| 29 | 22081-22734 | + | 217 | Hypothetical protein | *Escherichia* phage moskry (4e-156) | 100 | 0 | N |
| 30 | 22852-23751 | + | 299 | Single-stranded DNA binding protein [PF08804; gp32; gp32 DNA binding protein like] | *Escherichia* phage vB_EcoM_G2285 (0.0) | 100 | 0 | N |
| 31 | 23866-24117 | + | 83 | Hypothetical protein | *Escherichia* phage moskry (2e-54) | 100 | 0 | N |

| | | | | | | | | |
|---|---|---|---|---|---|---|---|---|
| 32 | 24128-24520 | + | 130 | Hypothetical protein | Enterobacteria phage ATK47 (2e-88) | 99.2 | 0 | N |
| 33 | 24582-24830 | + | 82 | Hypothetical protein | *Escherichia* phage AV114 (3e-53) | 100 | 0 | N |
| 34 | 24833-25420 | + | 195 | Dihydrofolate reductase | *Escherichia* phage SF (1e-139) | 98.4 | 0 | N |
| 35 | 25417-26277 | + | 286 | DTMP (thymidylate) synthase [PF00303; Thymidylat_synt; Thymidylate synthase] | *Escherichia* phage vB_EcoM-172859UKE1 (0.0) | 100 | 0 | N |
| 36 | 26278-26532 | + | 84 | Hypothetical protein | *Escherichia* phage vB_EcoM_NBG1 (3e-52) | 97.6 | 0 | N |
| 37 | 26620-28875 | + | 751 | NrdA-like aerobic NDP reductase large subunit [IPR039718; Rrm1; Ribonucleoside-diphosphate reductase large subunit] | *Escherichia* phage ST0 (0.0) | 99.7 | 0 | N |
| 38 | 28929-30107 | + | 392 | Ribonucleoside-diphosphate reductase subunit beta [PF00268; Ribonuc_red_sm; Ribonucleotide reductase, small chain] | *Escherichia* phage ST0 (0.0) | 100 | 0 | N |
| 39 | 30134-30544 | + | 136 | Putative endonuclease [PF01541; GIY-YIG; GIY-YIG catalytic domain] | *Escherichia* phage vB_EcoM_MM02 (4e-94) | 99.2 | 0 | N |
| 40 | 30599-31723 | + | 374 | RNA ligase 1 [PF09511; RNA_lig_T4_1; RNA ligase] | *Escherichia* phage S143_2 (0.0) | 99.4 | 0 | N |
| 41 | 31723-32286 | + | 187 | Inhibitor of host transcription [PF17527; ALC; Phage ALC protein] | *Escherichia* phage p000v (3e-114) | 97.3 | 0 | N |
| 42 | 32274-32630 | + | 118 | Hypothetical protein | *Escherichia* phage vB_EcoM_EP32a (7e-75) | 97.4 | 1 | N |
| 43 | 32627-32917 | + | 96 | Hypothetical protein | *Shigella* phage phi25-307 (3e-65) | 100 | 0 | Y |
| 44 | 32914-33132 | + | 72 | Hypothetical protein | *Escherichia* phage phiC120 (8e-45) | 100 | 0 | N |
| 45 | 33190-33489 | + | 99 | Hypothetical protein | *Escherichia* phage vB_EcoM_JS09 (8e-64) | 100 | 0 | N |
| 46 | 33489-33680 | + | 63 | Hypothetical protein | *Escherichia* phage vB_EcoM-172859UKE1 (3e-37) | 98.4 | 0 | N |
| 47 | 33677-34576 | + | 299 | Polynucleotide kinase [IPR044493 ; IPR044493 ; Polynucleotide kinase PNKP, C-terminal phosphatase domain] | *Shigella* phage phi25-307 (0.0) | 100 | 0 | N |

| | | | | | | | | |
|---|---|---|---|---|---|---|---|---|
| 48 | 34576-34767 | + | 63 | Hypothetical protein | *Escherichia* phage ST0 (5e-37) | 100 | 0 | N |
| 49 | 34757-34972 | + | 71 | Hypothetical protein | *Escherichia* phage vB_EcoM_JS09 (4e-44) | 100 | 0 | N |
| 50 | 34980-35255 | + | 91 | Hypothetical protein | *Escherichia* phage vB_EcoM_G2285 (7e-58) | 98.9 | 0 | N |
| 51 | 35316-35552 | + | 78 | Hypothetical protein | *Escherichia* phage vB_EcoM_JS09 (4e-49) | 100 | 0 | N |
| 52 | 35539-35664 | + | 41 | Hypothetical protein | *Shigella* phage SSE1 (4e-19) | 100 | 0 | N |
| 53 | 35673-36665 | + | 330 | Phospho-2-dehydro-3-deoxyheptonate aldolase [PF00793; DAHP_synth_1; DAHP synthetase I family] | *Shigella* phage SSE1 (0.0) | 100 | 0 | N |
| 54 | 36665-37246 | + | 193 | dCMP deaminase [PF00383; dCMP_cyt_deam_1; Cytidine and deoxycytidylate deaminase zinc-binding region] | *Escherichia* phage ST0 (8e-139) | 98.9 | 0 | N |
| 55 | 37248-37544 | + | 98 | Hypothetical protein | *Escherichia* phage ST0 (4e-65) | 100 | 0 | N |
| 56 | 37602-37934 | + | 110 | Head assembly chaperone protein [PF00166; Cpn10; Chaperonin 10 Kd subunit] | *Escherichia* phage RB69 (3e-71) | 99 | 0 | N |
| 57 | 38059-38307 | + | 82 | Lysis inhibition accessory protein [IPR034688; Linr3 ; Lysis inhibition accessory protein rIII] | *Escherichia* phage vB_EcoM_JS09 (1e-52) | 100 | 0 | N |
| 58 | 38471-38650 | + | 59 | Hypothetical protein | *Escherichia* phage AV116 (1e-31) | 100 | 0 | N |
| 59 | 38782-39150 | + | 122 | Hypothetical protein | *Escherichia* phage vB_EcoM_JS09 (1e-84) | 100 | 0 | N |
| 60 | 39225-39590 | + | 121 | Hypothetical protein | *Escherichia* phage p000y (2e-85) | 100 | 0 | N |
| 61 | 39626-40240 | + | 204 | Hypothetical protein | *Escherichia* phage 308Ecol101PP (2e-149) | 99 | 0 | N |
| 62 | 40295-40492 | + | 65 | Hypothetical protein | *Escherichia* phage vB_EcoM_JS09 (1e-38) | 100 | 0 | N |
| 63 | 40482-40694 | + | 70 | Hypothetical protein | *Escherichia* phage 55 (7e-42) | 98.5 | 0 | N |

| | | | | | | | | |
|---|---|---|---|---|---|---|---|---|
| 64 | 40687-41145 | + | 152 | Hypothetical protein | *Shigella* phage Shf125875 (3e-107) | 98.6 | 0 | N |
| 65 | 41142-41978 | + | 278 | Putative phosphatase | *Escherichia* phage 308Ecol101PP (0.0) | 100 | 0 | N |
| 66 | 41981-42250 | + | 89 | Hypothetical protein | *Shigella* phage Shf125875 (1e-58) | 100 | 0 | N |
| 67 | 42247-43740 | + | 497 | DNA ligase [PF01068; DNA_ligase_A_M; ATP dependent DNA ligase domain] | *Escherichia* phage vB_EcoM_JS09 (0.0) | 99.8 | 0 | N |
| 68 | 43740-43928 | + | 62 | Hypothetical protein | *Shigella* phage SSE1 (1e-37) | 100 | 0 | N |
| 69 | 43984-46071 | + | 695 | ADP-ribosyltransferase exoenzyme [PF03496; ADPrib_exo_Tox; ADP-ribosyltransferase exoenzyme] | *Escherichia* coli O157 typing phage 3 (0.0) | 99.8 | 0 | N |
| 70 | 46130-46423 | + | 97 | Hypothetical protein | *Escherichia* phage AV113 (3e-63) | 97.9 | 0 | N |
| 71 | 46456-47418 | - | 320 | Tail tube | *Escherichia* phage vB_EcoM_JS09 (0.0) | 99.6 | 0 | N |
| 72 | 47418-48527 | - | 369 | Baseplate tail tube cap | *Escherichia* phage vB_EcoM_JS09 (0.0) | 100 | 0 | N |
| 73 | 48536-50308 | - | 590 | Hypothetical protein | *Escherichia* phage vB_EcoM_WFbE185 (0.0) | 99.8 | 0 | N |
| 74 | 50305-50775 | - | 156 | Baseplate distal hub subunit | *Shigella* phage Shf125875 (1e-110) | 100 | 0 | N |
| 75 | 50786-51958 | - | 390 | Putative baseplate hub subunit [PF09097; Phage-tail_1; Baseplate structural protein, domain 1] | *Escherichia* phage vB_EcoM_WFbE185 (0.0) | 99.4 | 0 | N |
| 76 | 51955-52707 | - | 250 | Baseplate hub assembly chaperone [PF12322; T4_baseplate; T4 bacteriophage base plate protein] | *Escherichia* phage vB_Eco_F27 (0.0) | 99.6 | 0 | N |
| 77 | 52755-53381 | + | 208 | Baseplate hub assembly chaperone | *Escherichia* phage AV111 (4e-153) | 99.5 | 0 | N |
| 78 | 53381-53779 | + | 132 | Putative baseplate wedge subunit | *Escherichia* phage vB_EcoM_G2469 (6e-90) | 99.2 | 0 | N |
| 79 | 53779-54273 | + | 164 | Recombination, repair and ssDNA binding protein | *Escherichia* phage moskry (3e-116) | 100 | 0 | N |
| 80 | 54273-54497 | + | 74 | Hypothetical protein | *Escherichia* phage vB_EcoM_PhAPEC2 (3e-46) | 100 | 0 | N |

| | | | | | | | | |
|---|---|---|---|---|---|---|---|---|
| 81 | 54530-54697 | + | 55 | Hypothetical protein | *Escherichia* phage vB_EcoM_JS09 (3e-31) | 98.1 | 0 | N |
| 82 | 54755-54985 | - | 76 | DNA helicase | *Escherichia* phage RB69 (6e-45) | 100 | 0 | N |
| 83 | 55011-56525 | - | 504 | ATP-dependent DNA helicase [PF00271 ; Helicase_C ; Helicase conserved C-terminal domain] | *Escherichia* phage PTK (0.0) | 100 | 0 | N |
| 84 | 56576-57244 | + | 222 | Minor head protein inhibitor of protease | *Escherichia* phage vB_EcoM_JS09 (5e-157) | 99.5 | 0 | N |
| 85 | 57254-58672 | + | 472 | Hypothetical protein [PF00801; PKD; PKD domain] | *Escherichia* phage JN02 (0.0) | 97.6 | 0 | N |
| 86 | 58774-58968 | + | 64 | Hypothetical protein | *Escherichia* phage RB69 (2e-36) | 100 | 0 | N |
| 87 | 58965-59216 | + | 83 | Hypothetical protein | *Escherichia* phage vB_EcoM_JS09 (4e-55) | 100 | 0 | N |
| 88 | 59336-60334 | + | 332 | RNA ligase 2 [PF09414; RNA_ligase; RNA ligase] | *Escherichia* phage p000y (0.0) | 99.7 | 0 | N |
| 89 | 60366-61649 | - | 427 | Capsid vertex protein [PF07068; Gp23; Major capsid protein Gp23] | *Escherichia* phage ST0 (0.0) | 100 | 0 | N |
| 90 | 61751-62020 | + | 89 | Hypothetical protein | *Escherichia* phage vB_EcoM_JS09 (3e-57) | 100 | 0 | N |
| 91 | 62073-63641 | - | 522 | Major head protein [PF07068; Gp23; Major capsid protein Gp23] | *Escherichia* phage vB_EcoM_JS09 (0.0) | 100 | 0 | N |
| 92 | 63659-64471 | - | 270 | Head scaffolding protein | *Escherichia* phage vB_EcoM_JS09 (0.0) | 100 | 0 | N |
| 93 | 64505-65146 | - | 213 | Head maturation protease | *Escherichia* phage vB_EcoM_JS09 (1e-152) | 100 | 0 | N |
| 94 | 65146-65571 | - | 141 | Head scaffolding protein | *Escherichia* phage phiC120 (3e-95) | 99.2 | 0 | N |
| 95 | 65571-65807 | - | 78 | Prohead core protein | *Escherichia* phage vB_EcoM_NBG1 (1e-42) | 98.2 | 0 | N |
| 96 | 65807-67378 | - | 523 | Portal protein | *Escherichia* phage vB_EcoM_JS09 (0.0) | 100 | 0 | N |
| 97 | 67463-67954 | - | 163 | Tail protein [PF06841; Phage_T4_gp19; T4-like virus tail tube protein gp19] | *Escherichia* phage RB69 (5e-116) | 99.3 | 0 | N |

| | | | | | | | | |
|---|---|---|---|---|---|---|---|---|
| 98 | 68067-70049 | - | 660 | Tail sheath protein | *Shigella* phage SSE1 (0.0) | 99.5 | 0 | N |
| 99 | 70080-71915 | - | 611 | Terminase large subunit [IPR044267 ; Terminase_large_su_gp17-like ; Terminase, large subunit, gp17-like] | *Escherichia* phage HX01 (0.0) | 100 | 0 | N |
| 100 | 71899-72393 | - | 164 | Terminase small subunit | *Shigella* phage SHSML-52-1 (4e-116) | 100 | 0 | N |
| 101 | 72403-73224 | - | 273 | Tail sheath stabilizer | *Escherichia* phage vB_EcoM_PhAPEC2 (0.0) | 100 | 0 | N |
| 102 | 73277-74041 | - | 254 | Head closure Hc2 | *Escherichia phage* vB_EcoM_JS09 (0.0) | 99.6 | 0 | N |
| 103 | 74043-74969 | - | 308 | Head-tail adaptor Ad2 | *Escherichia* phage vB_EcoM_JS09 (0.0) | 99.6 | 0 | N |
| 104 | 75002-76450 | - | 482 | Fibritin | *Escherichia* phage JN02 (0.0) | 99.7 | 0 | N |
| 105 | 76450-78033 | - | 527 | Straight tail fiber proteion [PF14928; S_tail_recep_bd; Short tail fibre protein receptor-binding domain] | *Shigella* pahge SSE1 | 99.2 | 0 | N |
| 106 | 78030-78689 | - | 219 | Baseplate wedge subunit | *Escherichia* phage vB_EcoM_JS09 (1e-158) | 100 | 0 | N |
| 107 | 78689-80494 | - | 601 | Baseplate wedge subunit [PF07880; T4_gp9_10; Bacteriophage T4 gp9/10-like protein] | *Escherichia* phage vB_EcoM_JS09 (0.0) | 100 | 0 | N |
| 108 | 80494-81366 | - | 290 | Baseplate wedge tail fiber connector [PF07880; T4_gp9_10; Bacteriophage T4 gp9/10-like protein] | *Shigella* phage SSE1 (0.0) | 99.6 | 0 | N |
| 109 | 81439-82443 | - | 334 | Baseplate wedge subunit | *Escherichia* phage vB_Eco_F31 (0.0) | 100 | 0 | N |
| 110 | 82436-85534 | - | 1032 | Baseplate wedge initiator [IPR034697 ; GP7_T4 ; Baseplate wedge protein gp7] | *Escherichia* phage moskry (0.0) | 99.8 | 1 | N |
| 111 | 85531-87504 | - | 657 | Baseplate wedge subunit | *Escherichia* phage ST0 (0.0) | 100 | 0 | N |
| 112 | 87513-87806 | - | 97 | Phospholipase | *Escherichia* phage AV110 (6e-64) | 100 | 0 | N |
| 113 | 87809-88324 | - | 171 | Putative 18.5 kDa protein | *Escherichia* phage S143_2 (3e-121) | 99.4 | 0 | N |

| | | | | | | | | |
|---|---|---|---|---|---|---|---|---|
| 114 | 88328-90061 | - | 577 | Baseplate hub subunit tail lysozyme | *Shigella* phage JK45 (0.0) | 100 | 0 | N |
| 115 | 90061-90636 | - | 191 | Baseplate wedge subunit | *Escherichia* phage vB_EcoM_JS09 (1e-137) | 100 | 0 | N |
| 116 | 90698-91147 | + | 149 | Phage head completion protein [PF08722; Tn7_Tnp_TnsA_N; TnsA endonuclease N terminal] | *Escherichia* phage vB_Eco_NicPhage (6e-106) | 100 | 0 | N |
| 117 | 91150-91971 | + | 273 | DNA end protector protein | *Escherichia* phage vB_EcoM_JS09 (0.0) | 100 | 0 | N |
| 118 | 92074-92658 | + | 194 | Tail tube terminator protein [PF06841; Phage_T4_gp19; T4-like virus tail tube protein gp19] | *Escherichia* phage S143_2 (9e-143) | 100 | 0 | N |
| 119 | 92712-93446 | + | 244 | Deoxynucleotide monophosphate kinase | *Escherichia* phage S143_2 (1e-177) | 100 | 0 | N |
| 120 | 93451-93681 | + | 76 | Tail fiber assembly protein | *Escherichia* phage AV117 (4e-42) | 98.6 | 0 | N |
| 121 | 93681-94136 | + | 151 | RNA ligase | *Escherichia* phage vB_EcoM_JS09 (1e-107) | 100 | 0 | N |
| 122 | 94214-94486 | + | 90 | Hypothetical protein | *Escherichia* phage S143_2 (1e-56) | 100 | 0 | N |
| 123 | 94547-94855 | + | 102 | Hypothetical protein | *Escherichia* phage S143_2 (1e-67) | 100 | 0 | N |
| 124 | 94925-95110 | + | 61 | Hypothetical protein | *Escherichia* phage vB_EcoP_EP32B (3e-33) | 100 | 2 | N |
| 125 | 95112-95492 | + | 126 | Hypothetical protein | *Escherichia* phage p000y(8e-9) | 100 | 0 | N |
| 126 | 95495-95782 | + | 95 | Hypothetical protein | *Escherichia* phage ST0 (5e-63) | 100 | 0 | N |
| 127 | 96730-97074 | + | 114 | Hypothetical protein | *Escherichia* phage S143_2 (1e-79) | 100 | 0 | N |
| 128 | 97450-98076 | + | 208 | Hypothetical protein | *Escherichia* phage ST0 (5e-150) | 100 | 0 | N |
| 129 | 98187-98492 | + | 101 | Hypothetical protein | *Escherichia* phage HX01 (2e-67) | 100 | 0 | N |
| 130 | 98560-98724 | + | 54 | Hypothetical protein | *Escherichia* phage RB69 (1e-30) | 100 | 0 | N |

| | | | | | | | | |
|---|---|---|---|---|---|---|---|---|
| 131 | 98771-98998 | + | 75 | Hypothetical protein | *Shigella* phage phi25-307 (2e-47) | 98.6 | 0 | N |
| 132 | 99069-99662 | + | 197 | Hypothetical protein | *Escherichia* phage SF (2e-135) | 99.4 | 0 | N |
| 133 | 99712-100308 | + | 198 | Hypothetical protein | *Shigella* phage SSE1 (5e-144) | 99.4 | 0 | N |
| 134 | 100651-101013 | + | 120 | Hypothetical protein | *Escherichia* phage HX01 (2e-81) | 98.3 | 2 | N |
| 135 | 101006-101311 | + | 101 | Hypothetical protein | *Escherichia* phage vB_EcoM_JS09 (2e-66) | 99 | 0 | N |
| 136 | 101321-101593 | + | 90 | Hypothetical protein | *Escherichia* phage phiE142 (2e-59) | 98.8 | 0 | N |
| 137 | 101603-101800 | + | 65 | Hypothetical protein | *Escherichia* phage phiC120 (1e-37) | 98.4 | 0 | N |
| 138 | 101863-102102 | + | 79 | Hypothetical protein | *Escherichia* phage AlbertHofmann (2e-51) | 98.7 | 0 | N |
| 139 | 102127-103083 | + | 318 | Hypothetical protein | *Escherichia* phage vB_EcoM_JS09 (0.0) | 99 | 0 | N |
| 140 | 103154-103459 | + | 101 | Hypothetical protein | *Shigella* phage phi25-307 (1e-66) | 98 | 2 | N |
| 141 | 103461-104147 | + | 228 | Hypothetical protein | *Escherichia* phage ChristianSchoenbein (5e-116) | 96.4 | 0 | N |
| 142 | 104147-104638 | + | 163 | Membrane protein | *Shigella* phage Shf125875 (6e-113) | 99.3 | 2 | N |
| 143 | 104635-104871 | + | 78 | Hypothetical protein | *Escherichia* phage vB_EcoM_PhAPEC2 (7e-46) | 98.7 | 0 | N |
| 144 | 104861-105319 | + | 152 | NudE nudix hydrolase [PF00293; NUDIX; NUDIX domain] | *Escherichia* phage RB69 (1e-109) | 99.3 | 0 | N |
| 145 | 105354-105842 | + | 162 | Putative baseplate hub subunit and tail lysozyme [PF00959; Phage_lysozyme; Phage lysozyme] | *Escherichia* phage vB_EcoM_G53 (5e-116) | 98.1 | 0 | N |
| 146 | 105839-106120 | + | 93 | Internal head protein | *Shigella* phage Shf125875 (8e-58) | 100 | 0 | N |
| 147 | 106179-106592 | + | 137 | Endonuclease V N-glycosylase UV repair enzyme | *Escherichia* phage HX01 (1e-96) | 99.2 | 0 | N |

| | | | | | | | | |
|---|---|---|---|---|---|---|---|---|
| 148 | 106605-106859 | + | 84 | Hypothetical protein | *Escherichia* phage PHB12 (2e-51) | 100 | 0 | N |
| 149 | 106923-107237 | + | 104 | Hypothetical protein | *Escherichia* phage vB_EcoM_JS09 (3e-70) | 100 | 0 | N |
| 150 | 107264-107566 | + | 100 | Hypothetical protein | *Escherichia* phage AV117 (2e-64) | 99 | 0 | N |
| 151 | 107680-108219 | + | 179 | Hypothetical protein | *Escherichia* phage ST0 (2e-130) | 99.4 | 0 | Y |
| 152 | 108216-108524 | + | 102 | Hypothetical protein | *Escherichia* phage vB_EcoM_PhAPEC2 (2e-70) | 99 | 0 | N |
| 153 | 108531-108893 | + | 120 | Autonomous glycyl radical cofactor GrcA [PF01228; Gly_radical; Glycine radical] | *Escherichia* phage vB_EcoM_PhAPEC2 (3e-82) | 100 | 0 | N |
| 154 | 108893-109117 | + | 74 | Hypothetical protein | *Escherichia* phage vB_EcoM_JS09 (1e-46) | 98.6 | 0 | N |
| 155 | 109107-109352 | + | 81 | Hypothetical protein | *Escherichia* phage GADS24 (2e-50) | 97.5 | 0 | N |
| 156 | 109352-109651 | + | 99 | Hypothetical protein | *Shigella* phage Shf125875 (8e-67) | 100 | 0 | N |
| 157 | 109708-110166 | + | 152 | Endoribonuclease | *Escherichia* phage ATK47 (8e-108) | 100 | 0 | N |
| 158 | 110175-110717 | + | 180 | Hypothetical protein | *Shigella* phage SSE1 (7e-128) | 99.4 | 0 | Y |
| 159 | 110714-111061 | + | 115 | Vs valyl-tRNA synthetase modifier | *Escherichia* phage RB69 (3e-77) | 99.1 | 0 | Y |
| 160 | 111054-111521 | + | 155 | Phosphatase [PF01661; Macro; Macro domain] | *Escherichia* phage F2 (3e-110) | 100 | 0 | N |
| 161 | 111518-111730 | + | 70 | Hypothetical protein | *Escherichia* phage vB_EcoM_IME537 (5e-45) | 100 | 0 | N |
| 162 | 111727-111933 | + | 68 | Hypothetical protein | *Escherichia* phage F2 (3e-42) | 98.5 | 0 | N |
| 163 | 111930-112112 | + | 60 | Hypothetical protein | *Escherichia* phage APCEc01 (1e-32) | 98.3 | 0 | N |
| 164 | 112122-112703 | + | 193 | Thymidine kinase [PF00265; TK; Thymidine kinase] | *Escherichia coli* O157 typing phage 3 (1e-139) | 98.9 | 0 | N |

| | | | | | | | | |
|---|---|---|---|---|---|---|---|---|
| 165 | 112731-112943 | + | 70 | Hypothetical protein | *Escherichia* phage HX01 (3e-41) | 98.5 | 0 | N |
| 166 | 112956-113258 | + | 100 | Hypothetical protein | *Escherichia* phage PHB12 (1e-67) | 98 | 1 | Y |
| 167 | 113360-113539 | + | 59 | Hypothetical protein | *Escherichia* phage vB_EcoM_PhAPEC2 (1e-34) | 98.3 | 0 | N |
| 168 | 113547-113711 | + | 54 | Hypothetical protein | *Escherichia* phage vB_EcoM-ZQ3 (2e-32) | 100 | 0 | N |
| 169 | 113708-113803 | + | 31 | Hypothetical protein | *Escherichia* phage vB_EcoM_JS09 (1e-12) | 100 | 1 | N |
| 170 | 113803-114018 | + | 71 | Hypothetical protein | *Escherichia* phage PNJ-6 (9e-44) | 100 | 0 | N |
| 171 | 114064-114186 | + | 40 | Hypothetical protein | *Escherichia* phage ST0 (4e-18) | 100 | 1 | N |
| 172 | 114183-114362 | + | 59 | Hypothetical protein | *Shigella* phage SHSML-52-1 (4e-34) | 100 | 0 | N |
| 173 | 114364-114894 | + | 176 | Hypothetical protein | *Shigella* phage phi25-307 (1e-126) | 99.4 | 0 | N |
| 174 | 114904-115377 | + | 157 | Hypothetical protein | *Escherichia* phage PNJ-6 (3e-109) | 100 | 2 | N |
| 175 | 115377-116363 | + | 328 | Nucleotidyltrasnferase | *Shigella* phage SSE1 (0.0) | 100 | 0 | N |
| 176 | 116395-116661 | + | 88 | Hypothetical protein | *Escherichia* phage vB_EcoM_G2285 (5e-57) | 100 | 1 | Y |
| 177 | 116779-117759 | + | 326 | Hypothetical protein [IPR003593 ; AAA+_ATPase ; AAA+ ATPase domain] | *Escherichia* phage vB_EcoM_JS09 (0.0) | 100 | 0 | N |
| 178 | 117898-118185 | + | 95 | Hypothetical protein | *Escherichia* phage APCEc01 (1e-60) | 97.8 | 0 | N |
| 179 | 118244-118771 | + | 175 | Hypothetical protein | *Escherichia* phage vB_EcoM_JS09 (8e-123) | 100 | 0 | N |
| 180 | 118834-119829 | + | 331 | Thioredoxin | *Escherichia* phage PNJ-6 (0.0) | 99.7 | 0 | N |
| 181 | 119885-120820 | + | 311 | Thioredoxin | *Escherichia* phage HX01 (0.0) | 100 | 0 | N |

| | | | | | | | | |
|---|---|---|---|---|---|---|---|---|
| 182 | 120883-121833 | + | 316 | Hypothetical protein | *Shigella* phage SSE1 (0.0) | 98.4 | 0 | N |
| 183 | 121833-122138 | + | 101 | Hypothetical protein | *Escherichia* phage vB_EcoM_JS09 (4e-69) | 100 | 0 | N |
| 184 | 122138-122551 | + | 137 | Hypothetical protein | *Escherichia* phage APCEc01 (1e-95) | 100 | 2 | N |
| 185 | 122544-122807 | + | 87 | Phage-associated thioredoxin | *Escherichia* phage RB69 (1e-57) | 100 | 0 | N |
| 186 | 122804-123160 | + | 118 | Hypothetical protein | *Shigella* phage Shf125875 (2e-79) | 100 | 0 | N |
| 187 | 123289-123459 | + | 56 | Hypothetical protein | *Escherichia* phage p000v (3e-32) | 98.2 | 0 | N |
| 188 | 123461-123874 | + | 137 | Protease inhibitor | *Escherichia* phage GADS24 (6e-95) | 100 | 0 | N |
| 189 | 123884-124063 | + | 59 | Hypothetical protein | *Escherichia* phage vB_EcoM_TU01 (2e-35) | 98.3 | 0 | N |
| 190 | 124102-124575 | + | 157 | Endonuclease VII | *Escherichia* phage vB_EcoM_JS09 (8e-113) | 100 | 0 | N |
| 191 | 124572-126389 | + | 605 | Ribonucleotide reductase of class III [PF13597; NRDD; Anaerobic ribonucleoside-triphosphate reductase] | *Escherichia* phage vB_EcoM-172859UKE1 (0.0) | 99.5 | 0 | N |
| 192 | 126386-126856 | + | 156 | Anaerobic NTP reductase small subunit [IPR012837 ; NrdG; Ribonucleoside-triphosphate reductase activating, anaerobic] | *Escherichia* phage vB_EcoM-ZQ3 (6e-112) | 100 | 0 | N |
| 193 | 126967-127182 | + | 71 | Hypothetical protein | *Escherichia* phage vB_EcoM_JS09 (4e-41) | 100 | 1 | N |
| 194 | 127185-127502 | + | 105 | Hypothetical protein | *Escherichia* coli O157 typing phage 3 (1e-58) | 97.1 | 0 | N |
| 195 | 127468-127791 | + | 107 | Glutaredoxin | *Escherichia* phage vB_EcoM_JS09 (7e-71) | 100 | 0 | N |
| 196 | 127958-128140 | + | 60 | Hypothetical protein | *Escherichia* phage vB_EcoM_JS09 (7e-36) | 100 | 0 | N |
| 197 | 128137-128385 | + | 82 | Hypothetical protein | *Escherichia* phage vB_EcoM-172859UKE1 (2e-50) | 100 | 0 | N |

| | | | | | | | | |
|---|---|---|---|---|---|---|---|---|
| 198 | 128393-128686 | + | 97 | Hypothetical protein | *Escherichia* phage PTK (7e-63) | 98.9 | 0 | N |
| 199 | 128694-128828 | + | 44 | Hypothetical protein | *Shigella* phage Shf125875 (2e-23) | 100 | 0 | N |
| 200 | 128825-129025 | + | 66 | Hypothetical protein | *Escherichia* phage ST0 (9e-42) | 100 | 0 | N |
| 201 | 129089-129328 | + | 79 | Hypothetical protein | *Escherichia* phage p000v (3e-50) | 100 | 0 | N |
| 202 | 129395-129727 | + | 110 | Hypothetical protein | *Shigella* phage phi25-307 (4e-73) | 100 | 0 | N |
| 203 | 129724-129951 | + | 75 | Hypothetical protein | *Escherichia* phage ST0 (1e-44) | 100 | 0 | N |
| 204 | 129948-130217 | + | 89 | Hypothetical protein | *Escherichia* phage moskry (3e-58) | 98.8 | 0 | N |
| 205 | 130290-130847 | + | 185 | Late sigma transcription factor [IPR046386 ; T4_sigma-like_factor ; RNA polymerase sigma-like factor] | *Escherichia* phage UGJNEcP1 (1e-134) | 100 | 0 | N |
| 206 | 130837-131046 | + | 69 | Hypothetical protein | *Escherichia* phage APCEc01 (5e-42) | 100 | 0 | N |
| 207 | 131048-131371 | + | 107 | Hypothetical protein | *Escherichia* phage p000v (1e-68) | 99 | 0 | N |
| 208 | 131592-131768 | + | 58 | Hypothetical protein | *Escherichia* phage HX01 (5e-35) | 100 | 0 | N |
| 209 | 131838-132857 | + | 339 | SbcD-like subunit of palindrome specific endonuclease [PF00149; Metallophos; Calcineurin-like phosphoesterase] | *Escherichia* phage APCEc01 (0.0) | 99.1 | 0 | N |
| 210 | 132854-133111 | + | 85 | Hypothetical protein | *Shigella* phage phiE142 (5e-53) | 97.6 | 0 | N |
| 211 | 133098-133337 | + | 79 | DUF5487 family protein | *Escherichia* phage RB69 (7e-50) | 98.7 | 0 | N |
| 212 | 133334-135022 | + | 562 | Recombination-related endonuclease [PF13476; AAA_23; AAA domain] | *Escherichia* phage FP43 (0.0) | 99.6 | 0 | N |
| 213 | 135077-135265 | + | 62 | Hypothetical protein | *Escherichia* phage RB69 (6e-38) | 100 | 0 | N |

| | | | | | | | | |
|---|---|---|---|---|---|---|---|---|
| 214 | 135278-135694 | + | 138 | RpbA RNA polymerase binding protein | *Escherichia* phage RB69 (1e-97) | 99.2 | 0 | N |
| 215 | 135737-136423 | + | 228 | Putative sliding clamp [PF09116; gp45-slide_C; gp45 sliding clamp, C terminal] | *Escherichia* phage vB_EcoM_WFL6982 (4e-164) | 99.5 | 0 | N |
| 216 | 136499-137461 | + | 320 | Clamp loader of DNA polymerase [PF00004; AAA; ATPase family associated with various cellular activities (AAA)] | *Shigella* phage Shf125875 (0.0) | 99.6 | 0 | N |
| 217 | 137463-138026 | + | 187 | DNApol clamp loader large subunit [PF16790; Phage_clamp_A; Bacteriophage clamp loader A subunit] | *Escherichia* phage AlbertHofmann (1e-133) | 99.4 | 0 | N |
| 218 | 138029-138397 | + | 122 | Translation repressor [PF01818; Translat_reg; Bacteriophage translational regulator] | *Escherichia* phage RB69 (5e-84) | 100 | 0 | N |
| 219 | 138479-141190 | + | 903 | DNA polymerase [PF00136; DNA_pol_B; DNA polymerase family B] | *Escherichia* phage vB_EcoM_JS09 (0.0) | 100 | 0 | N |
| 220 | 141231-141866 | + | 211 | Arabinose 5-phosphate isomerase [PF01380; SIS; SIS domain] | *Escherichia* phage RB69 | 100 | 0 | N |
| 221 | 141863-142006 | + | 47 | Hypothetical protein | *Escherichia* phage F2 (2e-23) | 100 | 1 | N |
| 222 | 142048-143733 | + | 561 | NTP-transferase domain-containing protein [PF00483; NTP_transferase; Nucleotidyl transferase] | *Escherichia* phage JN02 (0.0) | 99.6 | 0 | N |
| 223 | 143733-144119 | + | 128 | Phosphoheptose isomerase | *Escherichia* phage UGJNEcP1 (9e-91) | 100 | 0 | N |
| 224 | 144177-145337 | + | 386 | Peptidase U32 | *Escherichia* phage 308Ecol101PP (0.0) | 100 | 0 | N |
| 225 | 145334-145489 | + | 51 | Hypothetical protein | *Escherichia* phage ChristianSchoenbein (5e-27) | 100 | 0 | N |
| 226 | 145576-146292 | + | 238 | Thymidylate synthase [PF00303; Thymidylat_synt; Thymidylate synthase] | *Escherichia* phage HX01 (2e-179) | 99.5 | 0 | N |
| 227 | 146292-147191 | + | 299 | Hypothetical protein | *Escherichia* phage APCEc01 (0.0) | 100 | 0 | N |
| 228 | 147193-147741 | + | 182 | Thymidylate kinase | *Shigella* phage Shf125875 (9e-131) | 99.4 | 0 | N |
| 229 | 147842-149014 | + | 390 | RecA-like recombination protein [IPR013765 ; DNA_recomb/repair_RecA ; DNA recombination and | *Escherichia* phage mogra (0.0) | 99.7 | 0 | N |

| | | | | | | | | |
|---|---|---|---|---|---|---|---|---|
| | | | | repair protein RecA] | | | | |
| 230 | 149007-149348 | + | 113 | Head vertex assembly chaperone | *Shigella* phage Shf125875 (3e-76) | 99.1 | 0 | N |
| 231 | 149358-150800 | + | 480 | AAA family ATPase [PF03796; DnaB_C; DnaB-like helicase C terminal domain] | *Escherichia* phage APCEc01(0.0) | 100 | 0 | N |
| 232 | 150888-151262 | + | 124 | Hypothetical protein | *Escherichia* phage RB69 (6e-86) | 100 | 0 | N |
| 233 | 151318-151635 | + | 105 | Hypothetical protein | *Escherichia* phage OLB35 (2e-72) | 99 | 0 | N |
| 234 | 151632-151823 | + | 63 | Dmd discriminator of mRNA degradation | *Escherichia* phage F2 (1e-35) | 98.4 | 0 | N |
| 235 | 151825-152037 | + | 70 | Hypothetical protein | *Escherichia* phage RB69 (6e-44) | 100 | 0 | N |
| 236 | 152098-152466 | + | 122 | Immunity protein | *Escherichia* phage SF (1e-85) | 100 | 0 | Y |
| 237 | 152528-152776 | + | 82 | Immunity to superinfection | *Escherichia* coli O157 typing phage 3 (2e-48) | 100 | 2 | N |
| 238 | 152840-153133 | + | 97 | Spackle periplasmic protein [IPR046391 ; SPACKLE_T4 ; Protein spackle] | *Escherichia* phage RB69 (2e-68) | 98.9 | 0 | Y |
| 239 | 153135-153797 | + | 220 | Hypothetical protein | *Escherichia* phage vB_EcoM_JS09 (2e-155) | 98.1 | 0 | N |
| 240 | 153799-153996 | + | 65 | Hypothetical protein | *Escherichia* phage vB_EcoM_JS09 (6e-39) | 98.4 | 0 | N |
| 241 | 154016-154483 | + | 155 | Hypothetical protein | *Escherichia* phage vB_EcoM_JS09 (8e-110) | 100 | 0 | N |
| 242 | 154523-155545 | + | 340 | DNA primase subunit [IPR046392 ; PRIMASE_T4 ; DNA primase, bacteriophage T4] | *Escherichia* phage PNJ-6 (0.0) | 99.7 | 0 | N |
| 243 | 155542-155739 | - | 65 | Hypothetical protein | *Escherichia* phage GADS24 (3e-34) | 98.4 | 1 | N |
| 244 | 155828-156349 | + | 173 | dCTP pyrophosphatase | *Escherichia* phage AV117 (3e-125) | 99.4 | 0 | N |
| 245 | 156395-156631 | + | 78 | Virion structural protein [PF16855; Soc; Small outer capsid protein] | *Escherichia* phage vB_EcoM_JS09 (3e-50) | 100 | 0 | N |

| | | | | | | | | |
|---|---|---|---|---|---|---|---|---|
| 246 | 156926-157162 | + | 78 | Hypothetical protein | *Shigella* phage SSE1 (3e-48) | 100 | 0 | N |
| 247 | 157159-157338 | + | 59 | Hypothetical protein | *Escherichia* phage vB_EcoM_JS09 (2e-33) | 100 | 0 | N |
| 248 | 157338-157802 | + | 154 | Hypothetical protein | *Escherichia* phage AV117 (8e-109) | 100 | 0 | N |
| 249 | 157819-157983 | + | 54 | Hypothetical protein | *Escherichia* phage 348Ecol098PP (2e-30) | 100 | 0 | N |
| 250 | 157980-158144 | + | 54 | Hypothetical protein | *Escherichia* phage ST0 (5e-28) | 100 | 0 | N |
| 251 | 158200-158781 | + | 193 | Hypothetical protein [IPR043662 ; ModB-like ; NAD-protein ADP-ribosyltransferase ModB-like] | *Escherichia* phage vB_EcoM_JS09 (8e-142) | 100 | 0 | N |
| 252 | 158839-159447 | + | 202 | NAD--protein ADP-ribosyltransferase [IPR043662 ; ModB-like ; NAD-protein ADP-ribosyltransferase ModB-like] | *Escherichia* phage PTK (4e-149) | 99.5 | 0 | N |
| 253 | 159600-160346 | + | 248 | Srd anti-sigma factor | *Escherichia* phage RB69 (1e-178) | 100 | 0 | N |
| 254 | 160349-160660 | + | 103 | Hypothetical protein | *Escherichia* phage F2 (1e-68) | 100 | 0 | N |
| 255 | 160657-161970 | + | 437 | Dda-like helicase [PF18343; SH3_14; Dda helicase SH3 domain] | *Escherichia* phage ST0 (0.0) | 100 | 0 | N |
| 256 | 161980-162657 | + | 225 | Exonuclease [PF16473; DUF5051; 3' exoribonuclease, RNase T-like] | *Shigella* phage SSE1 (3e-166) | 100 | 0 | N |
| 257 | 162723-163217 | + | 164 | Transcriptional regulator | *Escherichia* phage vB_EcoM-172859UKE1 (2e-117) | 100 | 0 | N |
| 258 | 163279-163734 | + | 151 | Modifier of transcription | *Escherichia* phage vB_EcoM_SQ17 (8e-108) | 100 | 0 | N |
| 259 | 163744-164163 | + | 139 | Hypothetical protein | *Shigella* phage SSE1 (7e-95) | 99.2 | 0 | N |
| 260 | 164223-164747 | + | 174 | Hypothetical protein | *Escherichia* phage HX01 (3e-126) | 99.4 | 0 | N |
| 261 | 164805-165032 | + | 75 | Modifier of suppressor tRNAs | *Escherichia* phage AV112 (7e-47) | 100 | 0 | N |
| 262 | 165032-165442 | + | 136 | Hypothetical protein | *Escherichia* phage vB_EcoP_EP32B (5e-96) | 99.2 | 0 | N |

| | | | | | | | | |
|---|---|---|---|---|---|---|---|---|
| 263 | 165445-165624 | + | 59 | Hypothetical protein | *Escherichia* phage ChristianSchoenbein (1e-35) | 98.3 | 0 | N |
| 264 | 165627-166052 | + | 141 | Hypothetical protein | *Escherichia* phage ST0 (1e-95) | 100 | 0 | N |
| 265 | 166116-167933 | + | 605 | DNA topoisomerase II large subunit [PF02518; HATPase_c; Histidine kinase-, DNA gyrase B-, and HSP90-like ATPase] | *Escherichia* phage vB_EcoM-RPN187 (0.0) | 99.8 | 0 | N |
| 266 | 167976-169076 | + | 366 | Hypothetical protein | *Shigella* phage SSE1 (0.0) | 99.7 | 0 | N |
| 267 | 169169-169369 | + | 66 | rIIA lysis inhibitor | *Escherichia* phage AV109 (2e-36) | 98.4 | 0 | N |
