# Supplementary material for: Characterization of Five Lytic Bacteriophages as New Members of the Genus Mosigvirus, Infecting Multidrug-Resistant Shiga Toxin-Producing Escherichia coli and Their Antibiofilm Activity
Source: Viruses. 2025 Nov 13;17(11):1501. doi: 10.3390/v17111501 (PMC12656860; doi:10.3390/v17111501)
Supplement: Supplementary file 1 [file viruses-17-01501-s001.zip › Table S6.pdf]

Table S6. Genomic features of the *Mosigvirus* genus, as determined based on data extracted from the GenBank database.

Phage name (accession number)	Bacterial host	Genome size (bp)	G+C content (%)	Number of tRNAs	Number of ORFs	References
0157tp3 (KP869101)	*E. coli* O157:H7	168,733	37.5	2 (Met, Arg)	270	Cowley et al., 2015
Phi25-307 (MG589383)	*Shigella sonnei*	167,544	37.5	2 (Met, Arg)	266	-
ATK (KT184309)	*E. coli* O26:H11	170,020	37.5	2 (Met, Arg)	290	-
phiC120 (KY703222)	*E. coli* O157:H7	186,570	37.5	0	281	Amarillas et al., 2021
HX01 (JX536493)	APEC*	169,158	37.5	2	269	Tang et al., 2012
vB_EcoM_JS09 (KF582788)	APEC, genetically engineered *E. coli* B and K strain	169,148	37.5	2 (Met, Arg)	273	-
vB_EcoM_mar005P1 (LR027390)	*E. coli*	168,771	37.7	2 (Met, Arg)	267	Michniewski et al., 2019
APCEc01 (KR422352)	*E. coli, S. sonnei*	167,773	37.5	2 (Met, Arg)	272	Dalmasso et al., 2016
p000v (MK047717)	STEC	167,803	37.5	1 (Met)	263	Howard-Varona et al., 2018
vB_EcoM_PhAPEC2 (KF562341)	APEC	167,318	37.5	3 (Met, Arg, AsX)	254	Tsonos et al., 2014
RB69 (AY303349)	*E. coli* CAJ70	167,560	37.5	2 (Met, Arg)	273	-
Sf (MH359124)	*E. coli* O157:H7	168,695	37.5	0	262	-
Shf125875 (utam) (KM407600)	*Shigella flexneri* ATCC 2587	169,062	37.5	2 (Met, Arg)	267	-
SHSML-52-1 (KX130865)	*Shigella sonnei*	169,621	37.6	2 (Met, Arg)	285	Soffer et al., 2017

*APEC, Avian pathogenic *Escherichia coli*

References

Cowley, L. A., Beckett, S. J., Chase-Topping, M., Perry, N., Dallman, T. J., Gally, D. L., & Jenkins, C. (2015). Analysis of whole genome sequencing for the *Escherichia coli* O157: H7 typing phages. BMC genomics, 16, 1-13.

Amarillas, L., Villicaña, C., Lightbourn-Rojas, L., González-Robles, A., & León-Félix, J. (2021). The complete genome and comparative analysis of the phage phiC120 infecting multidrug-resistant *Escherichia coli* and *Salmonella* strains. G3, 11(2), jkab014.

Tang, F., Li, Y., Zhang, W., & Lu, C. (2012). Complete genome sequence of T4-Like *Escherichia coli* bacteriophage HX01.

Michniewski, S., Redgwell, T., Grigonyte, A., Rihtman, B., Aguilo-Ferretjans, M., Christie-Oleza, J., ... & Millard, A. D. (2019). Riding the wave of genomics to investigate aquatic coliphage diversity and activity. Environmental microbiology, 21(6), 2112-2128.

Dalmasso, M., Strain, R., Neve, H., Franz, C. M., Cousin, F. J., Ross, R. P., & Hill, C. (2016). Three new *Escherichia coli* phages from the human gut show promising potential for phage therapy. PloS one, 11(6), e0156773.

Howard-Varona, C., Vik, D. R., Solonenko, N. E., Gazitua, M. C., Hobbs, Z., Honaker, R. W., ... & Sullivan, M. B. (2018). Whole-genome sequences of phages P000v and P000y, which infect the bacterial pathogen *Shiga-toxigenic Escherichia coli*. Microbiology Resource Announcements, 7(20), 10-1128.

Howard-Varona, C., Vik, D. R., Solonenko, N. E., Li, Y. F., Gazitua, M. C., Chittick, L., ... & Sullivan, M. B. (2018). Fighting fire with fire: phage potential for the treatment of *E. coli* O157 infection. Antibiotics (Basel) 7: 101.

Tsonos, J., Oosterik, L. H., Tuntufye, H. N., Klumpp, J., Butaye, P., De Greve, H., ... & Goddeeris, B. M. (2014). A cocktail of in vitro efficient phages is not a guarantee for in vivo therapeutic results against avian colibacillosis. Veterinary microbiology, 171(3-4), 470-479.

Soffer, N., Woolston, J., Li, M., Das, C., & Sulakvelidze, A. (2017). Bacteriophage preparation lytic for *Shigella* significantly reduces *Shigella sonnei* contamination in various foods. PLoS One, 12(3), e0175256.
